# ﻿The Coleoptera of the Province of Prince Edward Island, Canada: 295 new records from Lindgren funnel traps and a checklist to species

**DOI:** 10.3897/zookeys.1107.82976

**Published:** 2022-06-22

**Authors:** Reginald P. Webster, Cory Hughes, Jon D. Sweeney

**Affiliations:** 1 24 Mill Stream Dr., Charters Settlement, New Brunswick, E3C1X1, Canada Unaffiliated New Brunswick Canada; 2 Natural Resources Canada, Canadian Forest Service, Atlantic Forestry Centre, 1350 Regent St., P.O. Box 4000, Fredericton, NB, E3B 5P7, Canada Natural Resources Canada, Canadian Forest Service, Atlantic Forestry Centre Fredericton Canada

**Keywords:** Beetles, Canada, checklist, Lindgren funnel traps, new records, Prince Edward Island, trapping surveys

## Abstract

The Coleoptera fauna of the province of Prince Edward Island has long been one of the most poorly known jurisdictions in Canada, with fewer than half the number of species recorded in the neighbouring provinces of New Brunswick and Nova Scotia. If much of the difference in species richness was due to less intensive sampling of the province compared to other parts of Atlantic Canada it was predicted that surveys with semiochemical-baited traps would detect many previously undetected species. Lindgren funnel traps were baited with longhorn beetle pheromones and host volatiles and placed in the canopy and understory of coniferous and deciduous trees at the Valleyfield, New Harmony, Auburn, and Brookvale Demonstration Woodlots during the summers of 2018 and 2019. Two hundred and ninety-five species of Coleoptera are newly recorded from Prince Edward Island from 53 families. One of these, the Palaearctic *Pityophagusferrugineus* (Linnaeus, 1760) is reported for the first time from North America and Canada. The families Lycidae, Derodontidae, Lymexylidae, Sphindidae, Cucujidae, Ripiphoridae, Salpingidae, and Nemonychidae are newly recorded for the province. A checklist of the Coleoptera of Prince Edward Island is provided.

## ﻿Introduction

**Figure 1.**
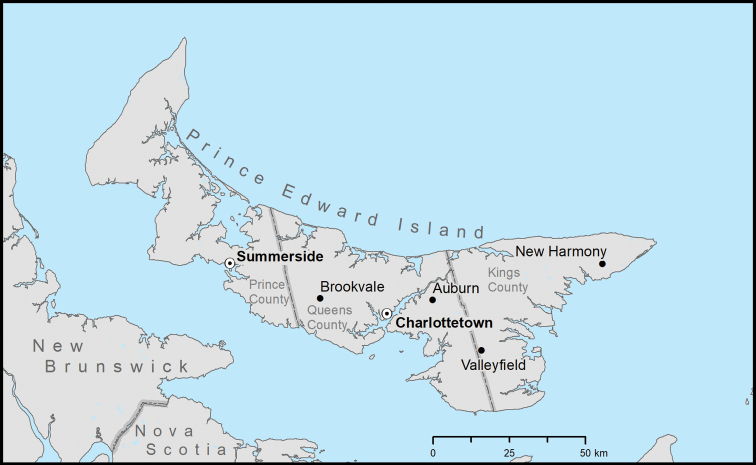
Map of Prince Edward Island, Canada showing location of the Valleyfield, New Harmony, Auburn, and Brookvale Demonstration Woodlots.

Prince Edward Island (PE) is one of the Maritime Provinces, a region of eastern Canada on the Atlantic coast that also includes New Brunswick (NB) and Nova Scotia (NS). It lies within the Atlantic Maritime Ecozone (McAlpine and Smith 2010). The Atlantic Ocean strongly influences the climate of the region resulting in cooler summers and warmer winters than in the interior. This is especially true for PE. This island province is located at 46 degrees latitude, 63 degrees longitude in the Gulf of St. Lawrence, and is separated from NB and NS by the Northumberland Straight. (Fig. 1). This crescent-shaped island is 224 km long and ranges in width from 6 km to 64 km. With a total area of 5,656 km^2^PE is the smallest province in Canada. Prior to European settlement of PE in the 1700s, 98% of the province was covered in Acadian Forest with American beech, yellow birch, sugar maple, white pine, eastern hemlock, red oak, and white ash on rich sites and species such as white spruce, black spruce, eastern larch, poplar, and white birch on poorer sites (Loo and Ives 2003; MacQuarrie and Lacroix 2003; Loo et al. 2010). Much of PE’s forest cover has since been replaced with agricultural fields and residential development, although some of the farmland has reverted to forest (Anon 2013). Currently, only about 44% of the island is covered with forest (Loo and Ives 2003; MacQuarrie and Lacroix 2003; Anon 2013). Much that remains is impoverished (re-growth) and only a few small relict old forests and areas of original vegetation are left (Loo and Ives 2003). The island has extensive salt and freshwater wetlands, often associated with small rivers and streams of which many experience a tidal influence, and extensive coastal sand dunes.

The Coleoptera fauna of PE has long been one of the most poorly known jurisdictions in Canada. The first checklist of the beetles of Canada by Bousquet (1991) listed only 340 species. However, since that checklist was published there has been a significant increase in the knowledge of the Coleoptera fauna through the contributions of several people and via various taxonomic revisions and general treatments of the Canadian fauna. The most significant contributions were made by Christopher G. Majka and collaborators through the examination of collections such as the Agriculture and Agri-Food collection, the University of Prince Edward Island collection, his own sampling, and sampling by others on the island. Many of these publications treated the fauna of the Maritime Provinces or Atlantic Canada as a whole and provided many new records for PE. Fifty-six families were treated in these publications and are listed here in the phylogenetic order used in Bousquet et al. (2013), followed by the number of new records in parentheses for PE: Gyrinidae (5) (Majka 2008c), Carabidae (18) (Majka 2005b, Majka et al. 2006a, 2008b), Haliplidae (1), Dytiscidae (6), Helophoridae (2), Hydrophilidae (12), (Majka 2008c), Histeridae (3) (Majka 2008a), Ptiliidae (3) (Majka and Sörensson 2007), Leiodidae (2) (Majka and Langor 2008), Silphidae (4) (Majka 2011g), Staphylinidae (24) (Majka 2012b, Klimaszewski and Majka 2007, 2008a,b, c, 2010), Eucinetidae (1) (Majka 2010a), Byrrhidae (4) (Majka et al. 2006b, Majka and Langor 2011b), Eucnemidae (4) (Majka 2007c), Throscidae (2) (Majka 2011b), Elateridae (27) (Majka and Johnson 2008), Lampyridae (5) (Majka 2012a), Dermestidae (9), Bostrichidae (5), Ptinidae (12) (Majka 2007b), Trogossitidae (1) (Majka 2011c), Cleridae (7) (Majka 2006b), Melyridae (1) (Majka 2005a), Erotylidae (2) (Majka 2007a), Monotomidae (2) (Majka and Bousquet 2010), Cryptophagidae (2) (Majka et al. 2010; Majka and Langor 2010), Silvanidae (2) (Majka 2008b), Phalacrididae (2) (Majka et al. 2008c), Laemophloeidae (1) Majka 2008b), Nitidulidae (9) (Majka and Cline 2006a), Cerylonidae (1) (Majka 2011d), Endomychidae (1) (Majka 2007a), Coccinellidae (14) (Majka and McCorquodale 2006), Corylophidae (1) (Majka and Cline 2006b), Latridiidae (13) (Majka et al. 2009), Mycetophagidae (1) (Majka 2010d), Ciidae (2) (Majka 2007d), Tetratomidae (2), Melandryidae (4) (Majka and Pollock 2006), Mordellidae (11) (Majka and Jackman 2006), Tenebrionidae (15) (Majka et al. 2008a), Synchroidae (1) Majka and Pollock 2006), Stenotracheliae (1) (Majka 2011a), Oedemeridae (1) (Majka and Langor 2011a), Pythidae (1), Pyrochroidae (3) (Majka 2006a), Anthicidae (6) (Majka 2011e), Aderidae (1) (Majka 2011f), Scraptiidae (3) (Majka and Pollock 2006), Cerambycidae (28) (Majka et al. 2007c), Chrysomelidae (10) (LeSage et al. 2007; Majka and LeSage 2007, 2008, 2010; LeSage and Majka 2010; Majka and Langor 2011c), Cerambycidae (28) (Majka et al. 2007c), Anthribidae (1), Attelabidae (1) (Majka et al. 2007b), Brentidae (2) (Majka et al. 2007a,b), Dryophthoridae (1) Brachyceridae (1) Curculionidae (54) (Majka et al. 2007a). Majka (2010b) obtained 11 new records from the families Carabidae (1), Staphylinidae (5), Ptinidae (1), Chrysomelidae (1), Brentidae (1), and Curculionidae (2) during a brief one-day survey of the Coleoptera of the Townshend Woodlot, using sweep netting, manually searching under rocks, in rotten wood, and on gill fungi. In another study, using maple sap as an attractant, eight new Coleoptera records were obtained from the families Staphylinidae (2), Scirtidae (2), Lampyridae (1), Nitidulidae (1), and Curculionidae (2) (Majka 2010c). One new tribe of Aleocharinae (Boreocypha) and a new species, *Boreocyphawebsteri* Klimaszewski & Langor, was described, in part, based on a specimen collected by Majka in St. Patricks, PE (Klimaszewski et al. 2011). Other publications by Jan Klimaszewski and collaborators included new staphylinid (Aleocharinae) records either as new species descriptions or new provincial records (Klimaszewski et. al. 2004 (2), 2007 (2)). Brunke et al. (2011) provided two new Staphylinidae (Staphylininae) records for the island and Webster et al. (2012a) reported two new species of Curculionidae. As a result of these and other publications, 599 species were added to the faunal list since Bousquet (1991) bringing the number of species known to occur in the province to 899 (Bousquet et al. 2013)

The impetus for this study was the low number of beetle species reported from PE (899) compared to those recorded from the neighbouring provinces of NB (2,703) and NS (2,286) in Bousquet et al. (2013). Since the publication of the Bousquet et al. (2013) checklist, 42 species have been added to the faunal list of PE in publications by Alarie (2016), Pentinsaari et al. (2019), Hammond and Chambers (2020), Webster et al. (2020, 2022) bringing the total known from the province to 941. However, many additional Coleoptera species have also been recorded in NB and NS (Webster 2016; Webster et al. 2016a, b, c, f, 2020, 2022; Hammond and Chambers 2020) during this same period, widening the gap even further. Currently, there are 3,152 and 2,338 species known from NB and NS, respectively.

Many of the new species records for NB and NS in the last decade resulted from specimens of target taxa and bycatch collected in numerous field experiments testing effects of semiochemical lures, trap height, and trap color on detection of Cerambycidae, Buprestidae, and Curculionidae in traps (e.g., Webster et al. 2016a, Flaherty et al. 2019; Rassati et al. 2019; Sweeney et al. 2020). We reasoned that much of the difference in species richness was due to less intensive sampling of PE compared to other parts of Atlantic Canada, as suggested by Majka et al. (2007b). Thus, we predicted that surveys with Lindgren funnel traps baited with semiochemicals placed in the canopy and understory of forests in different areas of PE would attract many previously undetected species that had already been collected using similar methods in NB and NS. However, other factors undoubtedly also explain the apparent lower beetle species richness on PE such as its smaller total land area (5,656 km^2^) and proportion of land with forest cover (44%) (Anon 2013) relative to NB (72,908 km^2^, 85%) (Nadeau et al. 2007) and NS (55,284 km^2^, 75%) (Anon 2017). PE is 7.8% and 9.8% of the land area of NB and NS, respectively.

## ﻿Methods and methods

### ﻿Collection methods

The purpose of this two-year study was to improve our knowledge of Coleoptera species composition on PE using relatively low maintenance survey methods, i.e., Lindgren funnel traps and a small number of flight intercept panel traps serviced about once per month. We placed 5–16 traps in each of three demonstration woodlots in 2018 and in the same three woodlots plus a fourth demonstration woodlot in 2019. Most of the traps were 12-unit Lindgren funnel traps. Black Lindgren traps are visually similar to tree trunks and are often effective for sampling species of Coleoptera that live in microhabitats associated with standing trees (Lindgren 1983). Green Lindgren traps are more effective than black traps for collecting certain genera of jewel beetles, e.g., *Agrilus* spp., especially when placed in the mid-upper canopy of trees (Rassati et al. 2019). When baited with various combinations of lures, these traps have been very effective at providing new species records and species new to science in the Maritime Provinces (Anderson and Klimaszewski 2012; Lopes-Andrade et al. 2016; Webster et al. 2016b, 2020, 2022) and Maine (DiGirolomo et al. 2021). In 2019, we also used one or two flight intercept panel traps (Alpha-Scents Inc., Canby, OR) customized by substituting white Coroplast panels (Coroplast, Vanceburg, KY) for the black panels.

Traps were baited with either: 1) a hardwood blend that consisted of five synthetic longhorn beetle pheromone lures (racemic 3-hydroxyhexan-2-one, racemic 3-hydroxyoctan-2-one, *syn*-2,3-hexanediols, fuscumol, and fuscumol acetate) plus an ultra-high release rate (UHR) ethanol lure; 2) a softwood blend that consisted of four pheromones (monochamol, ipsenol, fuscumol, and fuscumol acetate) plus UHR alpha-pinene and UHR ethanol; or 3) UHR ethanol by itself. The latter lure was used only in 2019, and only for the first month (7–8 May to 4–5 June), after which traps were baited with either the hardwood or softwood blend. These and similar lure combinations have proven effective at catching many species of bark- and wood-boring beetles (e.g., Miller et al. 2016; Flaherty et al. 2019). Traps were placed at two heights: 1) low traps were suspended from a rope tied between two trees that were separated by at least 2.0 m with the trap collecting cup 30–50 cm above ground; 2) canopy traps were suspended from a rope over a branch in the mid- or upper canopy using methods described in Hughes et al. (2014). We recorded the tree species in which each trap was placed (canopy traps) or nearest (low traps). All traps contained a saturated solution of table salt (NaCl) in water as a killing agent and preservative, with a drop of liquid dish detergent to reduce surface tension. Traps were sampled at approximately one-month intervals. At least one specimen of each species was vouchered at each study site each year. The number of traps used at each of the four study sites and site-specific details are described below.

### ﻿Study sites

We selected four of the six demonstration woodlots on PE (Fig. 1). These woodlots were created by the Department of Agriculture and Forestry as a means of providing woodlot owners, forestry contractors, and the public with examples of forest management to increase public awareness of forestry issues on the island, and provide recreational areas for hiking, bird watching, etc. The woodlots are a mixture of reclaimed farmland and older forest, and each contains a variety of forest cover types, including hardwoods, conifers, and young plantations.

#### Valleyfield Demonstration Woodlot

Located in Valleyfield in Kings Co., at 46.1356°N, 62.7198°W. This mature stand is dominated by red maple and eastern hemlock with patches of balsam fir, white and yellow birch, and some poplar. The site had some downed poplar and eastern hemlock. Twelve Lindgren funnel traps (five green traps placed 10–16 m high in the canopy, one low green and six low black traps) were deployed 12 June–13 September 2018. Six low black funnel traps, six canopy green funnel traps, and one low white panel trap were deployed 7 May–17 September 2019.

#### New Harmony Demonstration Woodlot

Located in Kings Co., at 46.3914°N, 62.2021°W. This mature stand is dominated with red maple, sugar maple, and red spruce with patches of white pine and poplar. Downed material is mostly poplar and spruce. Six low black funnel traps, six canopy green funnel traps (10–12 m in height), and one low white panel trap were deployed 8 May–17 September 2019.

#### Auburn Demonstration Woodlot

Located in Auburn in Queens Co., at 46.2882°N, 62.9267°W. This mature stand is dominated with white spruce and balsam fir with large tooth aspen with some snags present. Wild black raspberry is common in understory with downed woody material (mostly poplar). Twelve funnel traps (six green traps 12–14 m high in the canopy, three low green and three low black traps) were deployed 13 June–13 September 2018. Four funnel traps (two low black traps, two canopy green traps) and one low white panel trap were deployed 7 May–17 September 2019.

#### Brookvale Demonstration Woodlot

Located in Brookvale in Queens Co., at 46.2920°N, 63.4051°W. This stand is a mix of mature and young trees dominated by white spruce and white birch with a few white pine, Douglas fir, and poplar also present. Twelve Lindgren funnel traps (six green, six black, all low) were deployed 13 June–13 September 2018. Twelve funnel traps (eight low, four canopy (8–10 m in height), equal numbers of green and black) were deployed 7 May–17 September 2019, and an additional six low traps (two green funnel, two black funnel, two white panel traps) were deployed 4 June–17 September 2019.

#### Specimen preparation, determination, and photography

For each specimen the following data was provided on two labels: first label with province, county, township, name of demonstration woodlot (Auburn Demonstration Woodlot), GPS data presented in decimal degrees for the center of the study site, collection date (range of dates between sample collections), collector (all specimens/samples were collected by Cory Hughes); second label with forest type, Lindgren funnel trap, its color and height (1 m high or in canopy), tree species trap was deployed under (low traps) or in (canopy traps), trap number, and lure blend (hardwood blend or conifer blend).

Males of some species were dissected to confirm their identities. The genital structures were removed and dehydrated in absolute alcohol and either mounted in Canada balsam on celluloid micro slides or glued onto cards that were then pinned with the specimens from which they originated. Most specimens were determined by the senior author. Donald Bright (Colorado State University) determined the *Pityophthorus* and some other Scolytinae. It least one voucher specimen of each species from each site was deposited in either the Atlantic Forestry Centre (**AFC**) collection or in the Reginald Webster Collection (**RWC**) or both as specified in the species accounts below.

Images of the dorsal habitus of two specimens of *Pityophagusferrugineus* (Linnaeus) (Nitidulidae) were taken using an image processing system (Nikon SMZ 1500 stereoscopic microscope, Nikon DS-L3 Digital Camera, NIS Elements, and Adobe Photoshop software).

#### Data reported

Since much of the data on the labels is the same for each site (see site descriptions above), only the following data is provided for each new record: County, township, GPS data, collection date, low or canopy trap (if a canopy trap, also the tree species in which it was deployed), number of voucher specimens, and the collection in which they are deposited.

#### Distribution

All species are cited with their known distributions in Canada and Alaska using abbreviations for the states, provinces, and territories. New provincial records are indicated in **bold** under Distribution in Canada and Alaska. The following abbreviations are used in the text:

**AB** Alberta;

**AK** Alaska;

**BC** British Columbia;

**MB** Manitoba;

**NB** New Brunswick;

**NL & LB** Newfoundland and Labrador^*^;

**NS** Nova Scotia;

**NT** Northwest Territories;

**NU** Nunavut;

**ON** Ontario;

**PE** Prince Edward Island;

**QC** Quebec;

**SK** Saskatchewan;

**YT** Yukon Territory.

## ﻿Results and discussion

In this two-year study, 549 species in 70 families were recorded from the Valleyfield, New Harmony, Auburn, and Brookvale Woodlots (Table 1). Among these species were 300 species new to PE in 53 families. This represents 54.6% of the species collected during this study. Five of these new records [the adventive *Anthribusnebulosus* Forster, *Polydrususimpressifrons* Gyllenhal, *Hylastesopacus* Erichson, *Xyloborinussaxeseni* (Ratzeburg), and *X.germanus* (Blandford)] were previously reported by Webster et al. (2020). The families Lycidae, Derodontidae, Lymexylidae, Sphindidae, Cucujidae, Ripiphoridae, Salpingidae, and Nemonychidae are newly recorded for the province. With these additions, there are currently 1,236 Coleoptera species in 81 families in PE (Table 1).

**Table 1.** Number of new records by family from the Valleyfield, New Harmony, Auburn, and Brookvale Demonstration Woodlots collected during 2018–2019 with a comparison of species per family in the checklist in this publication to those recorded for PE in Bousquet et al. (2013). Families with ** are newly recorded from PE.

Taxa	Valleyfield	New Harmony	Auburn	Brookvale	Total	New Records	2013 Checklist	Additions	2022 Checklist
**Adephaga**									
Gyrinidae	0	0	0	0	0	0	9	3	12
Carabidae	4	6	9	5	16	2	174	2	176
Haliplidae	0	0	0	0	0	0	6	1	7
Dytiscidae	0	0	0	0	0	0	38	27	65
**Polyphaga**									
**Hydrophiloidea**									
Helophoridae	0	0	0	1	1	0	2	0	2
Hydrophilidae	0	1	0	1	2	1	19	1	20
Histeridae	3	1	4	3	5	4	14	4	18
**Staphylinoidea**									
Ptiliidae	0	0	0	0	0	0	3	0	3
Leiodidae	4	1	1	1	5	4	2	4	6
Silphidae	1	2	4	5	5	0	11	0	11
Staphylinidae	29	18	21	18	51	36	86	38	124
**Scarabaeoidea**									
Geotrupidae	0	1	0	0	1	1	1	1	2
Trogidae	0	0	1	1	1	0	2	0	2
Lucanidae	2	2	0	1	2	2	1	2	3
Scarabaeidae	3	2	6	6	10	6	13	6	19
**Scirtoidea**									
Eucinetidae	1	1	1	1	1	1	1	1	2
Scirtidae	0	1	2	1	3	2	7	2	9
**Buprestoidea**									
Buprestidae	3	0	2	1	5	3	12	3	15
**Byrrhoidea**									
Byrrhidae	0	0	0	1	1	0	4	0	4
Elmidae	0	0	0	0	0	0	4	0	4
Heteroceridae	0	0	0	0	0	0	1	0	1
Ptilodactylidae	0	0	0	0	0	0	1	0	1
**Elateroidea**									
Eucnemidae	9	3	8	10	13	9	4	9	13
Throscidae	2	0	2	2	2	0	2	0	2
Elateridae	36	17	28	29	45	18	49	18	67
Lycidae**	7	1	3	5	8	8	0	8	8
Lampyridae	4	1	4	3	5	1	6	1	7
Cantharidae	12	7	10	7	16	14	1	15	16
**Derodontoidea**									
Derodontidae**	0	0	1	0	1	1	0	1	1
**Bostrichoidea**									
Dermestidae	0	0	0	1	1	0	11	0	11
Bostrichidae	0	0	0	0	0	0	5	0	5
Ptinidae	8	3	7	4	11	6	15	6	21
**Lymexyloidea**									
Lymexylidae**	1	0	1	0	1	1	0	1	1
**Cleroidea**									
Trogossitidae	3	1	1	1	3	2	2	2	4
Cleridae	7	3	8	5	8	5	7	5	12
Melyridae	0	0	0	1	1	1	1	1	2
**Cucujoidea**									
Byturidae	1	0	1	1	1	0	1	0	1
Sphindidae**	3	0	2	2	3	3	0	3	3
Erotylidae	4	1	3	3	4	2	2	2	4
Monotomidae	3	1	3	2	4	3	2	3	5
Cryptophagidae	3	4	3	4	10	6	7	6	13
Silvanidae	2	2	1	2	2	2	2	2	4
Cucujidae**	1	1	1	0	1	1	0	1	1
Phalacrididae	1	0	0	0	1	0	2	0	2
Laemophloeidae	4	2	4	2	5	3	2	3	5
Kateretidae	0	0	1	2	2	2	1	2	3
Nitidulidae	14	8	11	17	23	13	12	13	25
Cerylonidae	2	1	1	1	2	1	1	1	2
Endomychidae	4	2	2	2	4	3	2	3	5
Coccinellidae	8	2	6	6	9	3	21	3	24
Corylophidae	1	0	1	0	1	1	1	1	2
Latridiidae	5	3	4	5	7	2	17	3	20
**Tenebrionoidea**									
Mycetophagidae	3	2	2	2	3	2	2	2	4
Ciidae	9	4	8	6	14	12	2	12	14
Tetratomidae	4	0	2	4	5	4	2	4	6
Melandryidae	15	6	14	14	17	12	5	12	17
Mordellidae	7	3	6	7	11	5	14	5	19
Ripiphoridae**	0	0	0	1	1	1	0	1	1
Zopheridae	1	0	1	1	2	2	1	2	3
Tenebrionidae	13	9	11	10	15	8	17	8	25
Synchroidae	0	0	1	1	1	0	1	0	1
Stenotrachelidae	1	0	1	1	1	0	2	0	2
Oedemeridae	2	1	0	2	3	3	1	3	4
Meloidae	0	0	0	0	0	0	0	1	1
Pythidae	1	0	1	0	1	0	2	0	2
Pyrochroidae	2	0	1	2	3	0	3	0	3
Salpingidae**	1	1	1	1	1	1	0	1	1
Anthicidae	0	0	0	0	0	0	7	0	7
Aderidae	0	0	1	0	1	0	1	0	1
Scraptiidae	3	2	3	3	3	0	3	0	3
**Chrysomeloidea**									
Cerambycidae	32	19	33	35	50	24	43	24	67
Megalopodidae	2	0	4	2	4	3	1	3	4
Orsodacnidae	1	0	1	1	1	0	1	0	1
Chrysomelidae	15	4	9	13	20	3	94	5	99
**Curculionoidea**									
Nemonychidae**	1	0	1	2	2	2	0	2	2
Anthribidae ^1^	0	0	1	0	1	1	1	1	2
Attelabidae	1	0	0	1	1	1	2	1	3
Brentidae	1	1	1	1	1	1	3	1	4
Dryophthoridae	1	1	1	1	1	1	4	1	5
Brachyceridae	0	0	0	0	0	0	1	0	1
Curculionidae ^2^	63	32	44	62	84	40	99	40	139
**Total**	**374**	**184**	**316**	**336**	**550**	**300**	**899**	**337**	**1236**

^1^One adventive species from this study was previously reported by Webster et al. (2020). ^2^Four adventive species from this study were previously reported by Webster et al. (2020).

The large number of new records clearly demonstrate the utility of Lindgren funnel traps for improving our knowledge of Coleoptera species distributions and supports our contention that less intensive sampling on PE relative to NB was partially responsible for the lower number of species previously known from the province. Although this study resulted in a significant increase in Coleoptera species known from PE, the 1,236 known species is still only 39.2% of the number known from NB (3,152). Other factors, such as the smaller total land area of PE (5,656 km^2^) compared to NB (72,908 km^2^), the lower proportion of land with forest cover on PE (44%) (Anon 2013) relative to NB (85%) due to agriculture and residential development (Nadeau et al. 2007), the lower range in elevation on PE (0–142 m) compared to NB (0–817 m), and the lower diversity of forest and habitat types on PE*vs.*NB, influence species richness in these two provinces. NB has significantly more forest and wetland types than PE (McAlpine and Smith 2010) and the number of Coleoptera species in each of these two provinces will clearly reflect this diversity difference with more species expected to occur in NB as a result. However, sampling has still been inadequate on the island. Many species live in specialized habitats and require more specialized and habitat targeted sampling. There were often families and species that were generally not well represented in Lindgren funnel trap collections in this study.

Among the families well represented in the funnel traps were the Eucnemidae, Elateridae, Lycidae, Cantharidae, Cleridae, Nitidulidae, Ciidae, Melandryidae, Cerambycidae, Nemonychidae, and Curculionidae (Table 1). Families not well represented were the Gyrinidae, Carabidae, Haliplidae, Dytiscidae, Hydrophilidae, Leiodidae, Staphylinidae, Buprestidae, Coccinellidae, and Chrysomelidae) (Table 1). The aquatic Coleoptera (Gyrinidae, Haliplidae, Dytiscidae, Hydrophilidae) require more specialized sampling methods such as dip netting in various kinds of aquatic habitats, as noted by Alarie (2016). These families are reasonably well known for PE as a result of his targeted sampling on the island. The low proportion (9.1%) of known Carabidae from PE captured in Lindgren funnel traps in this study (Table 1) was also not unexpected considering that most carabids are associated with habitats on the ground, as their common name, ground beetles, implies (Larochelle and Larivière 2003). Pitfall trapping, hand collecting under rocks and logs, litter sifting, treading wetland habitats, splashing pond and stream margins, and ultraviolet light trapping provided many new records for this family for NB (Webster and Bousquet 2008). Majka (2008b) added 14 species using primarily pitfall trapping. Continued use of these methods will undoubtedly result in more species records in this large family. In the current study 36 species of Staphylinidae were added to the faunal list of the province, bringing the total number of species known to 124 (Table 1.). Compared to NB, with 767 species (Webster 2016), this number is very low and a number of genera that are well represented in NB (*Lordithon*, *Tachinus*, *Tachyporus*, *Atheta*, *Philhygra*, *Gyrophaena*, *Oxypoda*, *Stenus*, *Philonthus*; Webster 2016) are poorly represented in PE, or absent (*Philhygra*, *Oxypoda*). For example, 31 species of *Gyrophaena* are known from NB (Webster 2016), only two are known from PE (Bousquet et al. 2013). Members of this genus are often abundant in fungi on the forest floor or on standing trees (Klimaszewski et al. 2009). There are undoubtedly many more than two species of this genus on PE. Other species of Staphylinidae are associated with wetland habitats (freshwater and salt marshes, vernal ponds, stream, pond and lake margins, intertidal habitats), forest floor habitats (leaf litter, moss, rotten logs, fungi), animal nests, and standing tree habitats (subcortical, tree holes) (Newton et al. 2001; Klimaszewski et al. 2018). Sampling of such habitats will undoubtedly result in a significant number of new records of this family for PE, as has been the case in NB (Webster et al. 2016d, e). The Staphylinidae will undoubtedly become the largest family of Coleoptera on PE once this family is adequately sampled. Sampling these habitats should also result in the discovery of many other Coleoptera species on PE. Sweeping and beating foliage in a variety of open and forested habitats should be productive for Coccinellidae, Chrysomelidae, and Curculionidae.

### ﻿Species accounts

Species with a † are adventive to Canada, species with a ‡ are either Holarctic or adventive to Canada, species with an * are Holarctic. The determination that a species was a new was based on absence from Bousquet et al. (2013), Webster et al. (2020, 2022), and other publications since Bousquet et al. (2013). The classification used below follows Bousquet et al. (2013) except for the Hydrophiloidea, which follows Short and Fikáĉek (2013). Below we report 295 species new to this island province and include a brief synopsis of the 81 families known from PE.

#### Family GYRINIDAE Latreille, 1810

Bousquet et al. (2013) listed nine species of Gyrinidae for PE. Alarie (2016) added another three species for a total of 12 species for the province. In this study, no members of this aquatic family of beetles were captured in Lindgren funnel traps.

#### Family CARABIDAE Latreille, 1802

Bousquet et al. (2013) reported 174 species of Carabidae from PE. A significant proportion of these records were from a two-year survey in 1987 and 1988 by Larochelle and Larivière (1990) who added 108 species. In this study, 16 species were captured in Lindgren funnel traps. Only two of these are new to PE. Interestingly, these two species are associated with trees (under bark of fallen or standing trees; Larochelle and Larivière 2003), and are species that one would expect to be captured in Lindgren funnel traps (Lindgren 1983).

##### Subfamily TRECHINAE Bonelli, 1810

**Tachyta (Tachyta) angulata Casey, 1918**

**New record. Kings Co.**, Valleyfield, 46.1356°N, 62.7198°W, 4.VI–3.VII.2019, low trap (1, AFC).

**Distribution in Canada and Alaska.**YT, BC, AB, SK, MB, ON, QC, NB, NS, **PE** (Bousquet et al. 2013).

##### Subfamily HARPALINAE Bonelli, 1810

**Cymindus (Pinacodera) limbata Dejean, 1831**

**New records. Kings Co.**, Valleyfield, 46.1356°N, 62.7198°W, 12.VI–3.VII.2018 (2), 3.VII–2.VIII.2018 (3), 2.VIII–13.IX.2018 (1), 4.VI–3.VII.2019 (1), canopy traps in hemlock (1), poplar (1), red maple (3), white spruce (1), low trap (1) (7, AFC); New Harmony, 46.3914°N, 62.2021°W, 3.VII–13.VIII.2019, low trap (1, AFC). **Queens Co.**, Auburn, 46.2882°N, 62.9267°W, 13.VI–3.VII.2018 (2), 3.VII–2.VIII.2018 (1), 3.VII–14.VIII.2019 (1), canopy traps in poplar (4, AFC); Brookvale, 46.2920°N, 63.4051°W, 3.VII–2.VIII.2018 (2), 3.VII–13.VIII.2019 (1), canopy trap in white pine (1), low traps (2) (3, AFC).

**Distribution in Canada and Alaska.** ON, QC, NB, **PE** (Bousquet et al. 2013).

#### Family HALIPLIDAE Aubé, 1836

Bousquet et al. (2013) listed six species of Haliplidae for PE. Alarie (2016) added *Haliplusconnexus* Matheson. No members of the aquatic family of beetles were captured in Lindgren funnel traps in this study.

#### Family DYTISCIDAE Leach, 1815

Bousquet et al. (2013) listed 38 species of Dytiscidae for PE. Alarie (2016) added another 27 species bringing the total number of known species for PE to 65. No members of this aquatic beetle family were captured in Lindgren funnel traps in this study.

#### Family HELOPHORIDAE Leach, 1815

Two species of Helophoridae are known from PE (Bousquet et al. 2013). One member of this aquatic group of beetles was captured in a Lindgren funnel trap at the Brookvale Demonstration Woodlot.

#### Family HYDROPHILIDAE Latreille, 1802

Nineteen species of Hydrophilidae are known from PE (Bousquet et al. 2013). Only two species of this family of mostly aquatic species was captured in Lindgren funnel traps. One is a new record and is reported below.

##### Subfamily ENOCHRINAE Short & Fikáček, 2013

***Cymbiodytavindicata* Fall, 1924**

**New record. Kings Co.**, New Harmony, 46.3914°N, 62.2021°W, 8.V–5.VI.2019, low trap (1, AFC).

**Distribution in Canada and Alaska.**NT, BC, AB, SK, MB, ON, QC, NB, NS, **PE**, LB, NF (Bousquet et al. 2013).

#### Family HISTERIDAE Gyllenhal, 1808

Bousquet et al. (2013) reported 14 species of this family for PE. In this study five species were captured in Lindgren funnel traps, four of which are new records for the province and are reported below.

##### Subfamily SAPRININAE Blanchard, 1845

***Gnathoncusbarbatus* Bousquet & Laplante, 1999**

**New record. Kings Co.**, Valleyfield, 46.1356°N, 62.7198°W, 12.VI–3.VII.2018, low trap (1, AFC). **Queens Co.**, Auburn, 46.2882°N, 62.9267°W, 3.VII–2.VIII.2018, low trap (1, AFC).

**Distribution in Canada and Alaska.**AK, BC, AB, SK, ON, QC, NB, NS, **PE** (Bousquet et al. 2013).

##### Subfamily HISTERINAE Gyllenhal, 1808

***Histercurtatus* J.E. LeConte, 1844**

**New record. Queens Co.**, Brookvale, 46.2920°N, 63.4051°W, 2.VIII–13.IX.2018, low trap (1, AFC).

**Distribution in Canada and Alaska.**MB, ON, QC, NB, NS, **PE** (Bousquet et al. 2013).

**Platysoma (Cylister) coarctatu m J.E. LeConte, 1844**

**New records. Kings Co.**, Valleyfield, 46.1356°N, 62.7198°W, 7.V–4.VII.2019 (1), 4.VI–3.VII.2019 (2), low traps (3, AFC). **Queens Co.**, Auburn, 46.2882°N, 62.9267°W, 3.VII–14.VIII.2019, low trap (1, AFC); Brookvale, 46.2920°N, 63.4051°W, 13.VI–3.VII.2018, low traps (3, AFC).

**Distribution in Canada and Alaska.**NT, BC, AB, SK, MB, ON, QC, NB, NS, **PE** (Bousquet et al. 2013).

**Platysoma (Cylistus) deficiens (Casey, 1924)**

**New record. Queens Co.**, Auburn, 46.2882°N, 62.9267°W, 13.VI–3.VII.2018 (1), 3.VII–2.VIII.2018 (2), 2.VIII–13.IX.2018 (1), low traps (4, AFC).

**Distribution in Canada and Alaska.**AB, SK, MB, ON, QC, NB, NS, **PE** (Bousquet et al. 2013).

#### Family PTILIIDAE Erichson, 1845

Only three species of this family of minute beetles are known from PE (Bousquet et al. 2013). None were captured in Lindgren funnel traps in this study.

#### Family LEIODIDAE Fleming, 1821

Only two species of Leiodidae were reported for PE by Bousquet et al. (2013). In this study, five species were recorded. Four of these are newly recorded for PE.

##### Subfamily LEIODINAE Fleming, 1821

***Anistomaglobososa* Hatch, 1829**

**New records. Kings Co.**, Valleyfield, 46.1356°N, 62.7198°W, 4.VI–3.VII.2019, 3.VII–13.VIII.2019, low traps (2, AFC). **Queens Co.**, Auburn, 46.2882°N, 62.9267°W, 13.VI–3.VII.2018, 4.VI–3.VII.2019, low traps (2, AFC); Brookvale, 46.2920°N, 63.4051°W, 4.VI–3.VII.2019, low trap (1, AFC).

**Distribution in Canada and Alaska.**BC, AB, SK, MB, ON, QC, NB, **PE**, NF (Bousquet et al. 2013).

##### Subfamily CHOLEVINAE Kirby, 1837

**Nemadus (Laferius) brachyderus (LeConte, 1863)**

**New record. Kings Co.**, Valleyfield, 46.1356°N, 62.7198°W, 3.VII–2.VIII.2018, 4.VI–3.VII.2019, low traps (2, AFC).

**Distribution in Canada and Alaska.**MB, ON, QC, NB, NS, **PE** (Bousquet et al. 2013).

**Nemadus (Nemadus) horni Hatch, 1933**

**New record. Kings Co.**, Valleyfield, 46.1356°N, 62.7198°W, 7.V–4.VI.2019, low trap (1, AFC).

**Distribution in Canada and Alaska.** ON, QC, NB, **PE** (Bousquet et al. 2013).

**Nemadus (Nemadus) triangulum Jeannel, 1936**

**New record. Kings Co.**, New Harmony, 46.3914°N, 62.2021°W, 3.VII–13.VIII.2019, low trap (1, AFC).

**Distribution in Canada and Alaska.** ON, QC, NB, NS, **PE** (Bousquet et al. 2013).

#### Family SILPHIDAE Latreille, 1806

Eleven species of Silphidae have been recorded from PE (Bousquet et al. 2013). Five of these were recorded in this study.

#### Family STAPHYLINIDAE Latreille, 1802

Fifty-one species of Staphylinidae were collected in Lindgren funnel traps in this study (Table 1). Thirty-six of these (70.6%) are new records for PE, bringing the total number of Staphylinidae known from PE to 124, demonstrating the effectiveness of these traps for detecting new members of this family. However, compared to NB with its 767 species (Webster 2016), this number is low and many additional species are likely to be found in the province with more habitat-targeted sampling.

##### Subfamily OMALIINAE MacLeay, 1825

***Acidotasubcarinata* Erichson, 1840**

**New record. Queens Co.**, Brookvale, 46.2920°N, 63.4051°W, 13.VI–3.VII.2018, low trap (1, AFC).

**Distribution in Canada and Alaska.** ON, QC, NB, NS, **PE**, NF (Bousquet et al. 2013).

**Eusphalerum (Eusphalerum) orientale (Bernhauer, 1912)**

**New record. Kings Co.**, New Harmony, 46.3914°N, 62.2021°W, 5.VI–3.VII.2019, 3.VII–13.VIII.2019, canopy trap in red maple (1), low trap (2, AFC).

**Distribution in Canada and Alaska.**BC, AB, MB, ON, QC, NB, NS, **PE**, NF (Bousquet et al. 2013).

**Eusphalerum (Eusphalerum) pothos (Mannerheim, 1843)**

**New record. Queens Co.**, Auburn, 46.2882°N, 62.9267°W, 13.VI–3.VII.2018, low trap (1, AFC).

**Distribution in Canada and Alaska.**AK, BC, AB, SK, ON, QC, NB, NS, **PE**, NF (Bousquet et al. 2013).

***Phloeonomuslaesicollis* (Mäklin, 1852)**

**New records. Kings Co.**, Valleyfield, 46.1356°N, 62.7198°W, 4.VI–3.VII.2019, low trap (1, AFC); New Harmony, 46.3914°N, 62.2021°W, 5.VI–3.VII.2019, low trap (1, AFC). **Queens Co.**, Brookvale, 46.2920°N, 63.4051°W, 13.VI–3.VII.2018 (2), 2.VIII–13.IX.2018 (1), 3.VI–13.VIII.2019 (1) low traps (4, AFC).

**Distribution in Canada and Alaska.**AK, BC, AB, SK, ON, QC, NB, NS, **PE**, NF (Bousquet et al. 2013).

##### Subfamily PSELAPHINAE Latreille, 1802

**Batrisodes (Excavodes) frontalis (LeConte, 1849)**

**New record. Queens Co.**, Brookvale, 46.2920°N, 63.4051°W, 3.VII–2.VIII.2018, low trap (1, AFC).

**Distribution in Canada and Alaska.**AB, MB, ON, QC, NB, **PE** (Bousquet et al. 2013).

**Batrisodes (Excavodes) lineaticollis (Aubé, 1833)**

**New record. Queens Co.**, Auburn, 46.2882°N, 62.9267°W, 13.VI–3.VII.2018, low trap (1, AFC).

**Distribution in Canada and Alaska.**BC, AB, MB, ON, QC, NB, NS, **PE** (Bousquet et al. 2013).

**Euplectus (Euplectus) duryi Casey, 1908)**

**New record. Queens Co.**, Auburn, 46.2882°N, 62.9267°W, 13.VI–3.VII.2018, canopy trap in poplar (1 ♂ (dissected), AFC).

**Distribution in Canada and Alaska.**BC, ON, QC, NB, NS, **PE** (Bousquet et al. 2013).

**Euplectus (Euplectus) elongatus Brendel, 1893**

**New record. Kings Co.**, Valleyfield, 46.1356°N, 62.7198°W, 7.V–4.VII.2019, low trap (1, AFC).

**Distribution in Canada and Alaska.** ON, QC, NB, NS, **PE** (Bousquet et al. 2013).

***Bibloporusbicanalis* (Casey, 1884)**

**New record. Kings Co.**, Valleyfield, 46.1356°N, 62.7198°W, 7.V–4.VII.2019, low trap (1, AFC).

**Distribution in Canada and Alaska.**QC, NB, NS, **PE** (Bousquet et al. 2013).

##### Subfamily PHLOEOCHARINAE Erichson, 1839

***Charhyphuspicipennis* (LeConte, 1863)**

**New records. Kings Co.**, Valleyfield, 46.1356°N, 62.7198°W, 2.VIII–13.IX.2018, 7.V–4.VI.2019, canopy trap in poplar (1), low trap (1) (2, AFC); New Harmony, 46.3914°N, 62.2021°W, 5.VI–3.VII.2019, canopy trap in white pine (1, AFC). **Queens Co.**, Auburn, 46.2882°N, 62.9267°W, 13.VI–3.VII.2018 (2), 7.V–4.VI.2019 (1), canopy trap in poplar (1), low traps (2) (3, AFC); Brookvale, 46.2920°N, 63.4051°W, 4.VI–3.VII.2013, canopy trap in white pine (1, AFC).

**Distribution in Canada and Alaska.**SK, ON, QC, NB, NS, **PE** (Bousquet et al. 2013).

##### Subfamily TACHYPORINAE MacLeay, 1825

***Bryoporusrufescens* LeConte, 1863**

**New records. Queens Co.**, Auburn, 46.2882°N, 62.9267°W, 3.VII–14.VIII.2019, canopy trap in poplar (1, AFC); Brookvale, 46.2920°N, 63.4051°W, 3.VII–13.VIII.2019, low trap (1, AFC).

**Distribution in Canada and Alaska.**BC, AB, SK, MB, ON, QC, NB, NS, **PE** (Bousquet et al. 2013).

***Carphacisnepigonensis* (Bernhauer, 1912)**

**New records. Kings Co.**, Valleyfield, 46.1356°N, 62.7198°W, 7.V–4.VI.2019, low trap (1, AFC); New Harmony, 46.3914°N, 62.2021°W, 13.VIII–17.IX.2019, low trap (1, AFC). **Queens Co.**, Auburn, 46.2882°N, 62.9267°W, 13.VI–3.VII.2018 (1), 3.VII–2.VIII.2018 (1), 4.VI–3.VII.2019 (1), low traps (3, AFC); Brookvale, 46.2920°N, 63.4051°W, 13.VI–3.VII.2018 (2), 7.V–4.VI.2019 (1), low traps (3, AFC).

**Distribution in Canada and Alaska.**BC, AB, SK, MB, ON, QC, NB, NS, **PE**, NF (Bousquet et al. 2013).

***Coproporusventriculus* (Say, 1832)**

**New records. Kings Co.**, Valleyfield, 46.1356°N, 62.7198°W, 3.VII–13.VIII.2019, low trap (1, AFC). **Queens Co.**, Auburn, 46.2882°N, 62.9267°W, 4.VI–3.VII.2019, low trap (1, AFC).

**Distribution in Canada and Alaska.**AK, NT, BC, AB, SK, MB, ON, QC, NB, NS, **PE** (Bousquet et al. 2013).

***Sepedophiluscinctulus* (Erichson, 1839)**

**New record. Kings Co.**, Valleyfield, 46.1356°N, 62.7198°W, 12.VI–3.VII.2018 (1), 3.VI–13.VIII.2019 (1), 7.V–4.VI.2019 (1), low traps (3, AFC).

**Distribution in Canada and Alaska.** ON, QC, NB, NS, **PE** (Bousquet et al. 2013).

***Sepedophiluslittoreus* (Linnaeus, 1758)**†

**New record. Kings Co.**, Valleyfield, 46.1356°N, 62.7198°W, 3.VII–2.VIII.2018, low trap (1, AFC).

**Distribution in Canada and Alaska.**BC, AB, SK, MB, ON, QC, NB, NS, **PE**, LF (Bousquet et al. 2013).

##### Subfamily ALEOCHARINAE Fleming, 1821

Most individuals of this subfamily were dissected to confirm their identity.

**Atheta (Dimetrota) fanatica Casey, 1910**

**New records. Kings Co.**, Valleyfield, 46.1356°N, 62.7198°W, 3.VII–2.VIII.2018 (1), 4.VI–3.VII.2019 (1), 3.VII–13.VIII.2019 (2), canopy traps in poplar (2), low traps (2) (2 ♂♂, 2 ♀♀, AFC). **Queens Co.**, Auburn, 46.2882°N, 62.9267°W, 3.VII–14.VIII.2019, canopy trap in poplar (1 ♀, AFC); Brookvale, 46.2920°N, 63.4051°W, 13.VI–3.VII.2018 (1), 3.VII–2.VIII.2018 (1), 3.VII–13.VIII.2019 (1), low traps (1 ♂, 2 ♀♀, AFC).

**Distribution in Canada and Alaska.**AK, YT, BC, QC, NB, NS, **PE**, LB (Bousquet et al. 2013).

***Peliopterathujae* (Klimaszewski & Webster, 2016)**

**New record. Kings Co.**, Valleyfield, 46.1356°N, 62.7198°W, 4.VI–3.VII.2019, low trap (1 ♂, AFC).

**Distribution in Canada and Alaska.**NB, **PE** (Webster et al. 2016e; Klimaszewski et al. 2018).

**Note.**Klimaszewski et al. (2018) transferred this species from the genus *Atheta* in which it was originally described to the genus *Pelioptera*.

***Cypheacurtula* (Erichson, 1837)**‡

**New record. Kings Co.**, Valleyfield, 46.1356°N, 62.7198°W, 7.V–4.VII.2019, low trap (1 ♂, RWC).

**Distribution in Canada and Alaska.**AB, QC, NB, **PE** (Klimaszewski et al. 2018).

**Note.** This species was originally described as a new species, *Agaricomorphavincenti* Klimaszewski & Webster in Webster et al. (2016e). However, Klimaszewski et al. (2018) later synonymized it with *Cypheacurtula* (Erichson, 1837). It is unclear if this is an adventive or a Holarctic species.

***Homolotaplana* (Gyllenhal, 1810)**†

**New record. Kings Co.**, Valleyfield, 46.1356°N, 62.7198°W, 7.V–4.VII.2019, low trap (1 ♂, AFC).

**Distribution in Canada and Alaska.**AB, MB, ON, NB, NS, **PE**, NF (Klimaszewski et al. 2018).

***Hylotaochracea* Casey, 1906**

**New records. Kings Co.**, New Harmony, 46.3914°N, 62.2021°W, 3.VII–13.VIII.2019, canopy trap in red maple (1 ♀, AFC). **Queens Co.**, Auburn, 46.2882°N, 62.9267°W, 3.VII–14.VIII.2019, canopy trap in poplar (1 ♂, AFC).

**Distribution in Canada and Alaska.**NT, SK, ON, QC, NB, NS, **PE** (Klimaszewski et al. 2018).

***Hylotacryptica* Klimaszewski & Webster, 2016**

**New records. Kings Co.**, Valleyfield, 46.1356°N, 62.7198°W, 4.VI–3.VII.2019, low trap (1 ♂, AFC). **Queens Co.**, Auburn, 46.2882°N, 62.9267°W, 3.VII–2.VIII.2018, low trap (1 ♀, AFC).

**Distribution in Canada and Alaska.**AB, NB, **PE** (Klimaszewski et al. 2016; Webster et al. 2016e, 2022).

***Mniusayukonensis* (Klimaszewski & Godin, 2012)**

**New record. Queens Co.**, Brookvale, 46.2920°N, 63.4051°W, 13.VI–3.VII.2018 (1), 3.VII–2.VIII.2018 (1), 7.V–4.VI.2019 (1), 4.VI–3.VII.2019 (1), 3.VII–13.VIII.2019 (1), low traps (3 ♂♂, 2 ♀♀, AFC).

**Distribution in Canada and Alaska.**YT, BC, ON, QC, NB, NS, **PE** (Klimaszewski et al. 2014, 2021).

***Phloeoporaoregona* Casey, 1906**

**New records. Kings Co.**, Valleyfield, 46.1356°N, 62.7198°W, 4.VI–3.VII.2019, low trap (1 ♀, AFC). **Queens Co.**, Brookvale, 46.2920°N, 63.4051°W, 13.VI–3.VII.2018, 4.VI–3.VII.2019, low traps (1 ♀, 1 not dissected, AFC).

**Distribution in Canada and Alaska.**NB, **PE** (Bousquet et al. 2013).

***Placusatachyporoides* (Walt, 1838)**†

**New record. Kings Co.**, New Harmony, 46.3914°N, 62.2021°W, 8.V–5.VI.2019, low trap (1 ♂, AFC).

**Distribution in Canada and Alaska.**BC, AB, SK, ON, QC, NB, NS, **PE** (Klimaszewski et al. 2018).

##### Subfamily PIESTINAE Erichson, 1839

***Siagoniumpunctatum* (LeConte, 1866)**

**New record. Kings Co.**, Valleyfield, 46.1356°N, 62.7198°W, 7.V–4.VI.2019, 3.VII–13.VIII.2019, low traps (2, AFC).

**Distribution in Canada and Alaska.** ON, QC, NB, NS, **PE** (Bousquet et al. 2013).

##### Subfamily OSORIINAE Erichson, 1839

***Clavilispinusprolixus* (LeConte, 1877)**

**New record. Queens Co.**, Auburn, 46.2882°N, 62.9267°W, 4.VI–3.VII.2019, white panel trap (1, RWC).

**Distribution in Canada and Alaska.**MB, QC, NB, **PE** (Bousquet et al. 2013).

##### Subfamily OXYTELINAE Fleming, 1821

***Coprophilusstriatulus* (Fabricius, 1792)**†

**New record. Kings Co.**, Valleyfield, 46.1356°N, 62.7198°W, 7.V–4.VI.2019, low trap (1, AFC).

**Distribution in Canada and Alaska.** ON, QC, NB, NS, **PE** (Bousquet et al. 2013).

##### Subfamily SCYDMAENINAE Leach, 1815

***Parascydmuscorpusculus* (Casey, 1897)**

**New record. Kings Co.**, Valleyfield, 46.1356°N, 62.7198°W, 12.VI–3.VII.2018, 7.V–4.VI.2019, low traps (2, AFC).

**Distribution in Canada and Alaska.** ON, QC, NB, NS, **PE** (Bousquet et al. 2013).

##### Subfamily STAPHYLININAE Latreille, 1802

***Tympanophoruspuncticollis* (Erichson, 1840)**

**New record. Queens Co.**, Auburn, 46.2882°N, 62.9267°W, 3.VII–14.VIII.2019, low trap (1, RWC).

**Distribution in Canada and Alaska.**BC, AB, SK, ON, QC, NB, NS, **PE**, NF (Bousquet et al. 2013).

***Bisniusblandus* (Gravenhorst, 1806)**

**New record. Queens Co.**, Brookvale, 46.2920°N, 63.4051°W, 4.VI–3.VII.2019, low trap (1, AFC).

**Distribution in Canada and Alaska.** ON, QC, NB, NS, **PE** (Bousquet et al. 2013).

***Bisniusquediinus* (Horn, 1884)**

**New record. Kings Co.**, New Harmony, 46.3914°N, 62.2021°W, 3.VII–13.VIII.2019, canopy trap in red maple (1, AFC).

**Distribution in Canada and Alaska.**QC, NB, **PE** (Bousquet et al. 2013).

***Philonthuscaeruleipenniscaeruleipennis* (Mannerheim, 1830)**

**New record. Queens Co.**, Auburn, 46.2882°N, 62.9267°W, 3.VII–2.VIII.2018 (1), 2.VIII–13.IX.2018 (1), canopy traps in poplar (2, AFC).

**Distribution in Canada and Alaska.**BC, AB, SK, MB, ON, QC, NB, NS, **PE** (Bousquet et al. 2013).

**Quedius (Microsaurus) bicoloris Smetana & Webster, 2011**

**New records. Kings Co.**, Valleyfield, 46.1356°N, 62.7198°W, 12.VI–3.VII.2018, 7.V–4.VI.2019, low traps (2, AFC); New Harmony, 46.3914°N, 62.2021°W, 5.VI–3.VII.2019, low trap (1, AFC).

**Distribution in Canada and Alaska.** ON, QC, NB, **PE** (Bousquet et al. 2013).

**Quedius (Microsaurus) canadensis (Casey, 1915)**

**New record. Kings Co.**, Valleyfield, 46.1356°N, 62.7198°W, 2.VIII–13.IX.2018, low trap (1, AFC).

**Distribution in Canada and Alaska.** ON, QC, NB, NS, **PE** (Bousquet et al. 2013).

**Quedius (Microsaurus) mesomelinus mesomelinus (Marsham, 1802)**†

**New records. Kings Co.**, Valleyfield, 46.1356°N, 62.7198°W, 3.VII–2.VIII.2018 (1) 2.VIII–13.IX.2018 (2), 4.VI–3.VII.2019 (1), canopy trap in red maple (1), low traps (3) (4 ♂♂ dissected, AFC). **Queens Co.**, Auburn, 46.2882°N, 62.9267°W, 3.VII–2.VIII.2018 (1), 3.VII–14.VIII.2019 (2), 14.VIII–17.IX.2019 (1), canopy traps in poplar (2) low traps (3) (5, (3 ♂♂ dissected), AFC); Brookvale, 46.2920°N, 63.4051°W, 13.VIII–17.IX.2019, low trap (1 ♂ (dissected), AFC).

**Distribution in Canada and Alaska.**AK, BC, AB, SK, MB, ON, QC, NB, NS, **PE**, LB, NF (Bousquet et al. 2013).

***Platydracusviridanus* (Horn, 1879)**

**New record. Kings Co.**, New Harmony, 46.3914°N, 62.2021°W, 3.VII–13.VIII.2019, canopy trap in sugar maple (1, AFC).

**Distribution in Canada and Alaska.** ON, QC, NB, NS, **PE** (Bousquet et al. 2013).

***Neohypnusbeckeri* Smetana, 1982**

**New records. Kings Co.**, New Harmony, 46.3914°N, 62.2021°W, 8.V–5.VI.2019, low trap (1, AFC). **Queens Co.**, Brookvale, 4.VI–3.VII.2019, white panel trap (1, AFC).

**Distribution in Canada and Alaska.** ON, QC, NB, **PE** (Bousquet et al. 2013).

#### Family GEOTRUPIDAE Latreille, 1802

One species of this family is newly recorded for PE. Only one other species, the adventive *Geotrupusstercorarius* (Linnaeus), was previously known from PE (Bousquet et al. 2013).

##### Subfamily GEOTRUPINAE Latreille, 1802

**Geotrupus (Anoplotrupes) balyi Jekel, 1865**

**New record. Kings Co.**, New Harmony, 46.3914°N, 62.2021°W, 3.VII–13.VIII.2019, canopy trap in sugar maple (1, AFC).

**Distribution in Canada and Alaska.**MB, ON, QC, NB, NS, **PE** (Bousquet et al. 2013).

#### Family TROGIDAE MacKeay, 1819

One of the two species of this family known from PE was captured in Lindgren funnel traps during this study.

#### Family LUCANIDAE Latreille, 1804

Two species of Lucanidae were recorded in this study. Both are new records for PE. Only one species, *Platycerusdepressus* LeConte, was previously known from the province (Bousquet et al. 2013).

##### Subfamily SYNDESINAE MacLeay, 1819

***Ceruchuspiceus* (Weber)**

**New records. Kings Co.**, Valleyfield, 46.1356°N, 62.7198°W, 3.VII–2.VIII.2018, 3.VII–13.VIII.2019, low traps (2, AFC); New Harmony, 46.3914°N, 62.2021°W, 3.VII–13.VIII.2019, low traps (2, AFC).

**Distribution in Canada and Alaska.**MB, ON, QC, NB, NS, **PE** (Bousquet et al. 2013).

##### Subfamily LUCANINAE Latreille, 1804

***Platycerusvirescens* (Fabricius, 1775)**

**New records. Kings Co.**, Valleyfield, 46.1356°N, 62.7198°W, 12.VI–3.VII.2018, low trap (1, AFC); New Harmony, 46.3914°N, 62.2021°W, 8.V–5.VI.2019, canopy trap in sugar maple (1, AFC). **Queens Co.**, Brookvale, 46.2920°N, 63.4051°W, 7.V–4.VI.2019, low traps (2, AFC).

**Distribution in Canada and Alaska.** ON, QC, NB, NS, **PE** (Bousquet et al. 2013).

#### Family SCARABAEIDAE Latreille, 1802

Thirteen species of Scarabaeidae were listed by Bousquet et al. (2013) from PE. In this study we recorded ten species, six being new to the province, bringing the total number of known species in PE to 19.

##### Subfamily APHODIINAE Leach, 1815

***Dialytesstriatulus* (Say, 1825)**

**New record. Queens Co.**, Brookvale, 46.2920°N, 63.4051°W, 3.VII–13.VIII.2019, low trap (1, AFC).

**Distribution in Canada and Alaska.**MB, ON, QC, NB, NS, **PE** (Bousquet et al. 2013).

##### Subfamily AEGIALIINAE Laporte, 1840

***Caeliusrufescens* (Horn, 1887)**

**New record. Queens Co.**, Brookvale, 46.2920°N, 63.4051°W, 13.VI–3.VII.2018 (2), 4.VI–3.VII.2019 (1), low traps (3, AFC).

**Distribution in Canada and Alaska.**AK, BC, AB, SK, MB, ON, QC, NB, NS, **PE** (Bousquet et al. 2013).

##### Subfamily MELOLONTHINAE Leach, 1819

***Dichelonyxalbicollis* Burmeister, 1855**

**New record. Kings Co.**, Valleyfield, 46.1356°N, 62.7198°W, 4.VI–3.VII.2019, canopy traps in poplar (1), white spruce (1) (2, AFC).

**Distribution in Canada and Alaska.** ON, QC, NB, NS, **PE** (Bousquet et al. 2013).

***Dichelonyxsubvittata* LeConte, 1856**

**New records. Queens Co.**, Auburn, 46.2882°N, 62.9267°W, 13.VI–3.VII.2018 (2), 4.VI–3.VII.2019, canopy traps in poplar (3, AFC); Brookvale, 46.2920°N, 63.4051°W, 13.VI–3.VII.2018 (1), 3.VII–2.VIII.2018 (1), 4.VI–3.VII.2019 (1), low traps (3, AFC).

**Distribution in Canada and Alaska.**SK, MB, ON, QC, NB, NS, **PE** (Bousquet et al. 2013).

***Sericaatracapilla* (Kirby, 1837)**

**New records. Kings Co.**, New Harmony, 46.3914°N, 62.2021°W, 3.VII–13.VIII.2019, canopy trap in red maple (1 ♂ (dissected), AFC). **Queens Co.**, Brookvale, 46.2920°N, 63.4051°W, 3.VII–2.VIII.2018, 4.VI–3.VII.2019, low traps (2 ♂♂ (dissected), AFC).

**Distribution in Canada and Alaska.**SK, MB, ON, QC, NB, NS, **PE** (Bousquet et al. 2013).

##### Subfamily CETONIINAE Leach, 1815

***Osmodermascabra* (Palisot de Beauvois, 1805)**

**New record. Queens Co.**, Auburn, 46.2882°N, 62.9267°W, 3.VII–2.VIII.2018, low trap (1, AFC).

**Distribution in Canada and Alaska.** ON, QC, NB, NS, **PE** (Bousquet et al. 2013).

#### Family EUCINETIDAE Lacordaire, 1857

One species of this family was recorded (at all four study sites) and it is a new record for PE. Only one species was previously known from PE (Bousquet et al. 2013).

***Eucinetusmorio* LeConte, 1853**

**New records. Kings Co.**, Valleyfield, 46.1356°N, 62.7198°W, 12.VI–3.VII.2018 (2), 4.VI–3.VII.2019 (1) low traps (3, AFC); New Harmony, 46.3914°N, 62.2021°W, 3.VII–13.VIII.2019, low trap (1, AFC). **Queens Co.**, Auburn, 46.2882°N, 62.9267°W, 13.VI–3.VII.2018, 4.VI–3.VII.2019, canopy trap in poplar (1), low trap (1) (2, AFC); Brookvale, 46.2920°N, 63.4051°W, 13.VI–3.VII.2018 (2), 4.VI–3.VII.2019 (1), low traps (3, AFC).

**Distribution in Canada and Alaska.** ON, QC, NB, NS, **PE** (Bousquet et al. 2013).

#### Family SCIRTIDAE Fleming, 1821

Bousquet et al. (2013) included seven species of Scirtidae for PE. In this study three species were recorded; two are new to the province.

##### Subfamily SCIRTINAE Fleming, 1821

***Contacyphonpadi* (Linnaeus, 1758)**†

**New record. Queens Co.**, Auburn, 46.2882°N, 62.9267°W, 14.VIII–17.IX.2019, canopy trap in poplar (1, AFC).

**Distribution in Canada and Alaska.**NB, NS, **PE** (Bousquet et al. 2013).

***Sacodespulchella* (Guérin-Méneville, 1843)**

**New record. Queens Co.**, Auburn, 46.2882°N, 62.9267°W, 3.VII–2.VIII.2018, canopy trap in poplar (1, AFC).

**Distribution in Canada and Alaska.** ON, QC, NB, NS, **PE** (Bousquet et al. 2013).

#### Family BUPRESTIDAE Leach, 1815

Twelve species of Buprestidae were recorded for PE by Bousquet et al. (2013). In this study we recorded five species. Three of these, all in genus *Agrilus*, are new to PE.

##### Subfamily AGRILINAE Laporte, 1835

***Agrilusgranulatusliragus* Barter & W.J. Brown, 1950**

**New records. Kings Co.**, Valleyfield, 46.1356°N, 62.7198°W, 2.VIII–13.IX.2018, 3.VII–13.VIII.2019, canopy traps in poplar (2, AFC). **Queens Co.**, Auburn, 46.2882°N, 62.9267°W, 3.VII–2.VIII.2018, canopy trap in poplar (1), low trap (1) (2, AFC)

**Distribution in Canada and Alaska.**AK, BC, AB, SK, MB, ON, QC, NB, NS, **PE**, NF (Bousquet et al. 2013).

***Agrilusmasculinus* Horn, 1891**

**New record. Kings Co.**, Valleyfield, 46.1356°N, 62.7198°W, 3.VII–2.VIII.2018 (2), 3.VII–13.VIII.2019 (1), canopy trap in red maple (2), in white spruce (1) (3, AFC).

**Distribution in Canada and Alaska.**SK, MB, ON, QC, NB, **PE** (Bousquet et al. 2013; Webster et al. 2016e).

***Agriluspolitus* (Say, 1825)**

**New record. Queens Co.**, Brookvale, 46.2920°N, 63.4051°W, 13.VI–3.VII.2018, low trap (1, RWC).

**Distribution in Canada and Alaska.**AK, YT, NT, BC, AB, SK, MB, ON, QC, NB, NS, **PE** (Bousquet et al. 2013).

#### Family BYRRHIDAE Latreille, 1804

Four species of this family are known from PE (Bousquet et al. 2013). We recorded one of these species, the adventive *Simplocariasemistriata* (Fabricius) at the Brookvale Woodlot.

#### Family ELMIDAE Curtis, 1830

Four members of this family have been recorded from PE (Bousquet et al. (2013). We did not record any of these aquatic beetles in this study.

#### Family HETEROCERIDAE MacLeay, 1825

One species is known from PE (Bousquet et al. 2013). None were recorded in our study.

#### Family PTILODACTYLIDAE Laporte, 1836

One species is known from PE (Bousquet et al. 2013). None were recorded in our study.

#### Family EUCNEMIDAE Eschscholtz, 1829

Lindgren funnel traps have proven very effective for detecting Eucnemidae in NB (Webster et al. 2012b, 2016e). The same was true for PE in this study, where 13 species were detected, nine of which are new records for the province (Table 1). The four other species were species previously known from PE (Bousquet et al. 2013), and thus all known species from PE were detected in this study using Lindgren funnel traps.

##### Subfamily MELASINAE Fleming, 1821

***Entomophthalmusrufiolus* (LeConte, 1866)**

**New record. Queens Co.**, Brookvale, 46.2920°N, 63.4051°W, 3.VII–13.VIII.2019, low trap (1, AFC).

**Distribution in Canada and Alaska.** ON, QC, NB, **PE** (Bousquet et al. 2013).

***Microrhaguspectinatus* LeConte, 1866**

**New records. Queens Co.**, Auburn, 46.2882°N, 62.9267°W, 13.VI–3.VII.2018, low traps (2, AFC); Brookvale, 46.2920°N, 63.4051°W, 13.VI–3.VII.2018, low trap (1, AFC).

**Distribution in Canada and Alaska.**BC, ON, QC, NB, NS, **PE** (Bousquet et al. 2013).

***Microrhagustriangularis* (Say, 1823)**

**New records. Kings Co.**, New Harmony, 46.3914°N, 62.2021°W, 3.VII–13.VIII.2019, low trap (1, AFC). **Queens Co.**, Auburn, 46.2882°N, 62.9267°W, 2.VIII–13.IX.2018, canopy trap in poplar (1, AFC); Brookvale, 46.2920°N, 63.4051°W, 3.VII–2.VIII.2018, low trap (1, AFC).

**Distribution in Canada and Alaska.** ON, QC, NB, NS, **PE** (Bousquet et al. 2013).

***Epiphaniscornutus* Eschscholtz, 1829**

**New record. Kings Co.**, Valleyfield, 46.1356°N, 62.7198°W, 3.VII–2.VIII.2018, 3.VII–13.VIII.2019, low traps (2, AFC).

**Distribution in Canada and Alaska.**AK, BC, SK, ON, QC, NB, NS, **PE**, NF (Bousquet et al. 2013).

***Dirrhagofarsusernae* Otto, Muona & McClarin, 2014**†

**New records. Kings Co.**, Valleyfield, 46.1356°N, 62.7198°W, 3.VII–2.VIII.2018 (17), 2.VIII–13.IX.2018 (1), 3.VII–13.VIII.2019 (1), canopy traps in poplar (8), in red maple (1), low traps (10) (19, AFC); New Harmony, 46.3914°N, 62.2021°W, 13.VIII–17.IX.2019, low trap (1, AFC). **Queens Co.**, Auburn, 46.2882°N, 62.9267°W, 3.VII–2.VIII.2018, 3.VII–14.VIII.2019, canopy trap in poplar (1), low trap (1) (2, AFC); Brookvale, 46.2920°N, 63.4051°W, 3.VII–2.VIII.2018 (3), 2.VIII–13.IX.2018 (1), 3.VII–13.VIII.2019 (1), low traps (5, AFC).

**Distribution in Canada and Alaska.**NB, NS, **PE** (Webster et al. 2016e, 2020).

**Note.**Otto et al. (2014) described *Dirrhagofarsusernae* from Ohio in the USA but considered it to be an introduction of a previously unknown species from Asia. It has become widespread in the northeastern USA (Otto et al. (2014). Webster et al. (2016e) first reported this species from Canada in NB and later reported it from NS (Webster et al. 2020). This appears to be the most common (many more specimens were collected than were vouchered) eucnemid on PE based on the records above.

***Isorhipisobliqua* (Say, 1839)**

**New records. Kings Co.**, Valleyfield, 46.1356°N, 62.7198°W, 12.VI–3.VII.2018 (1), 3.VII–2.VIII.2018 (1), 3.VII–13.VIII.2019 (1), canopy traps in poplar (1), in white spruce (1), low trap (1) (3, AFC); New Harmony, 46.3914°N, 62.2021°W, 3.VII–3.VIII.2019, canopy trap in red maple (1, AFC). **Queens Co.**, Auburn, 46.2882°N, 62.9267°W, 3.VII–2.VIII.2018, low trap (black) (1, AFC); Brookvale, 46.2920°N, 63.4051°W, 3.VII–2.VIII.2018 (2), 3.VII–13.VIII.2019 (1), low traps (3, AFC).

**Distribution in Canada and Alaska.**MB, ON, QC, NB, NS, **PE** (Bousquet et al. 2013).

***Isorhipisruficornis* (Say, 1823)**

**New record. Queens Co.**, Brookvale, 46.2920°N, 63.4051°W, 3.VII–13.VIII.2019, white panel trap (1, RWC).

**Distribution in Canada and Alaska.** ON, QC, NB, NS, **PE** (Bousquet et al. 2013).

##### Subfamily MACRAULACINAE Fleutiaux, 1923

***Dromaeolusharringtoni* Horn, 1886**

**New record. Kings Co.**, Valleyfield, 46.1356°N, 62.7198°W, 3.VII–2.VIII.2018, low trap (1, AFC).

**Distribution in Canada and Alaska.**MB, ON, QC, NB, **PE** (Bousquet et al. 2013).

***Onichodonorchesides* Newman, 1838**

**New records. Kings Co.**, Valleyfield, 46.1356°N, 62.7198°W, 3.VII–13.VIII.2019, low traps (2, AFC). **Queens Co.**, Auburn, 46.2882°N, 62.9267°W, 3.VII–2.VIII.2018 (3), 3.VII–14.VIII.2019 (1), canopy trap in red maple (1), low traps (3) (4, AFC); Brookvale, 46.2920°N, 63.4051°W, 2.VIII–13.IX.2018, low trap (1, AFC).

**Distribution in Canada and Alaska.** ON, QC, NB, NS, **PE** (Bousquet et al. 2013; Webster et al. 2022).

#### Family THROSCIDAE Laporte, 1840

Both known species of this family from PE listed by Bousquet et al. (2013) were captured in Lindgren funnel traps in this study.

#### Family ELATERIDAE Leach, 1815

Bousquet listed 49 species for PE. In this study, 45 species of Elateridae were captured in Lindgren funnel traps, 18 (40%) being new provincial records. Additional records will likely be detected with further sampling using these traps.

##### Subfamily AGRYPNINAE Candèze, 1857

***Laconauroratus* (Say, 1839)**

**New record. Queens Co.**, Auburn, 46.2882°N, 62.9267°W, 13.VI–3.VII.2018, 3.VII–14.VIII.2019, low traps (2, AFC); Brookvale, 46.2920°N, 63.4051°W, 3.VII–2.VIII.2018, low trap (1, AFC).

**Distribution in Canada and Alaska.**AB, MB, ON, QC, NB, NS, **PE** (Bousquet et al. 2013).

##### Subfamily DENDROMETRINAE Gistel, 1848

***Athousacanthus* (Say, 1839)**

**New records. Kings Co.**, Valleyfield, 46.1356°N, 62.7198°W, 3.VII–2.VIII.2018 (2), 2.VIII–13.IX.2018 (1), 3.VII–13.VIII.2019 (2), canopy trap in poplar (1), low traps (4) (5, AFC); New Harmony, 46.3914°N, 62.2021°W, 3.VII–13.VIII.2019, 13.VIII–17.IX.2019, low traps (2, AFC). **Queens Co.**, Brookvale, 46.2920°N, 63.4051°W, 3.VII–2.VIII.2018, 3.VII–13.VIII.2019, low traps (2, AFC).

**Distribution in Canada and Alaska.** ON, QC, NB, NS, **PE** (Bousquet et al. 2013).

***Athousbrightwelli* (Kirby, 1837)**

**New records. Kings Co.**, Valleyfield, 46.1356°N, 62.7198°W, 3.VII–2.VIII.2018 (7), 3.VII–13.VIII.2019 (2), canopy traps in hemlock (1), in white spruce (1), low traps (7) (9, AFC); New Harmony, 46.3914°N, 62.2021°W, 3.VII–13.VIII.2019, canopy trap in red maple (2, AFC). **Queens Co.**, Auburn, 46.2882°N, 62.9267°W, 3.VII–2.VIII.2018 (1), 3.VII–14.VIII.2019 (2), canopy traps in poplar (3, AFC); Brookvale, 46.2920°N, 63.4051°W, 3.VII–2.VIII.2018 (4), 3.VII–13.VIII.2019 (1), low traps (5, AFC).

**Distribution in Canada and Alaska.** ON, QC, NB, NS, **PE** (Bousquet et al. 2013).

***Athousposticus* (Melsheimer, 1845)**

**New record. Kings Co.**, Valleyfield, 46.1356°N, 62.7198°W, 3.VII–2.VIII.2018, low trap (1, RWC).

**Distribution in Canada and Alaska.** ON, QC, NB, NS, **PE** (Bousquet et al. 2013).

***Athousscapularis* (Say, 1839)**

**New record. Kings Co.**, Valleyfield, 46.1356°N, 62.7198°W, 3.VII–2.VIII.2018, canopy trap in red maple (2, AFC).

**Distribution in Canada and Alaska.** ON, QC, NB, NS, **PE** (Bousquet et al. 2013).

***Limoniusconfusus* LeConte, 1853**

**New records. Kings Co.**, Valleyfield, 46.1356°N, 62.7198°W, 12.VI–3.VII.2018 (1), 3.VII–2.VIII.2018 (1), 2.VIII–13.IX.2018 (1), 4.VI–3.VII.2019 (3), 3.VII–13.VIII.2019 (2), canopy traps in poplar (4), low traps (4) (8, AFC).

**Distribution in Canada and Alaska.** ON, QC, NB, NS, **PE** (Bousquet et al. 2013).

***Hypoganussulcicollis* (Say, 1833)**

**New record. Kings Co.**, Valleyfield, 46.1356°N, 62.7198°W, 3.VII–2.VIII.2018, low trap (1, AFC).

**Distribution in Canada and Alaska.**SK, MB, ON, QC, NB, NS, **PE** (Bousquet et al. 2013).

***Oxygonusmontanus* C. Schaeffer, 1917**

**New records. Kings Co.**, Valleyfield, 46.1356°N, 62.7198°W, 7.V–4.VI.2019, canopy trap in poplar (1, AFC); New Harmony, 46.3914°N, 62.2021°W, 8.V–5.VI.2019, low trap (1, AFC).

**Distribution in Canada and Alaska.**QC, NB, NS, **PE** (Bousquet et al. 2013).

***Pseudanostirushamatus* (Say, 1834)**

**New records. Kings Co.**, Valleyfield, 46.1356°N, 62.7198°W, 12.VI–3.VII.2018, canopy trap in red maple (1, AFC). **Queens Co.**, Brookvale, 46.2920°N, 63.4051°W, 13.VI–3.VII.2018, 4.VI–3.VII.2019, low traps (2, AFC).

**Distribution in Canada and Alaska.** ON, QC, NB, NS, **PE** (Bousquet et al. 2013).

##### Subfamily NEGASTRIINAE Nakane & Kishii, 1956

***Neohypdonustumescens* (LeConte, 1853)**

**New record. Kings Co.**, New Harmony, 46.3914°N, 62.2021°W, 5.VI–3.VII.2019, canopy trap in sugar maple (1, AFC).

**Distribution in Canada and Alaska.**AK, YT, NT, BC, AB, SK, MB, ON, QC, NB, NS, **PE** (Bousquet et al. 2013).

##### Subfamily ELATERINAE Leach, 1815

***Agriotesfucosus* (LeConte, 1853)**

**New record. Queens Co.**, Brookvale, 46.2920°N, 63.4051°W, 13.VI–3.VII.2018, low trap (1, AFC).

**Distribution in Canada and Alaska.**AB, SK, MB, ON, QC, NB, NS, **PE**, NF (Bousquet et al. 2013).

***Dalopiuscognatus* W.J. Brown, 1934**

**New records. Kings Co.**, Valleyfield, 46.1356°N, 62.7198°W, 4.VI–3.VII.2019, low trap (1 ♂ (dissected), AFC); New Harmony, 46.3914°N, 62.2021°W, 3.VII–13.VIII.2019, canopy trap in red maple (1 ♂ dissected), AFC). **Queens Co.**, Auburn, 46.2882°N, 62.9267°W, 13.VI–3.VII.2018, canopy trap in poplar (1, AFC); Brookvale, 46.2920°N, 63.4051°W, 13.VI–3.VII.2018 (3), 4.VI–3.VII.2019 (2), 3.VII–13.VIII.2019 (2), low traps (7 ♂♂ (dissected), AFC).

**Distribution in Canada and Alaska.** ON, QC, NB, NS, **PE** (Bousquet et al. 2013).

***Dalopiusfuscipes* W.J. Brown, 1934**

**New records. Kings Co.**, Valleyfield, 46.1356°N, 62.7198°W, 12.VI–3.VII.2018 (2), 7.V–4.VI.2019 (1), 4.VI–3.VII.2019 (1), canopy traps in red maple (2), in poplar (2) (4 ♂♂ dissected), AFC); New Harmony, 46.3914°N, 62.2021°W, 5.V–5.VI.2019 (1), 5.VI–3.VII.2019 (1), 3.VII–13.VIII.2019 (1), canopy trap in sugar maple (1), low traps (2) (3, (2 ♂♂ dissected), AFC).

**Distribution in Canada and Alaska.**QC, NB, NS, **PE** (Bousquet et al. 2013).

***Ampedusoblessus* (Say, 1833)**

**New records. Kings Co.**, Valleyfield, 46.1356°N, 62.7198°W, 4.VI–3.VII.2019 (2), 3.VII–13.VIII.2019 (1), canopy traps in poplar (1), in white pine (1), low trap (1) (3, AFC). **Queens Co.**, Auburn, 46.2882°N, 62.9267°W, 13.VI–3.VII.2018, canopy trap in poplar (1, AFC).

**Distribution in Canada and Alaska.**AB, SK, MB, ON, QC, NB, NS, **PE** (Bousquet et al. 2013; Webster et al. 2022).

***Ampedusprotervus* (LeConte, 1853)**

**New records. Kings Co.**, Valleyfield, 46.1356°N, 62.7198°W, 12.VI–3.VII.2018 (1), 7.V–4.VI.2019 (1), 3.VII–13.VIII.2019 (1), canopy traps in poplar (2), in white spruce (1) (3, AFC); New Harmony, 46.3914°N, 62.2021°W, 5.VI–3.VII.2019, canopy traps in red maple (1), in white pine (1) (2, AFC). **Queens Co.**, Auburn, 46.2882°N, 62.9267°W, 13.VI–3.VII.2018 (1), 3.VII–2.VIII.2018 (1), 3.VII–14.VIII.2014 (1), canopy traps in poplar (2), low trap (1) (3, AFC); Brookvale, 46.2920°N, 63.4051°W, 13.VI–3.VII.2018 (1), 3.VII–2.VIII.2018 (1), 3.VII–13.VIII.2019 (1), low traps (3, AFC).

**Distribution in Canada and Alaska.** ON, QC, NB, NS, **PE** (Bousquet et al. 2013).

***Ampedusvitiosus* (LeConte, 1853)**

**New record. Kings Co.**, Valleyfield, 46.1356°N, 62.7198°W, 4.VI–3.VII.2019, 3.VII–13.VIII.2019, low traps (2, AFC).

**Distribution in Canada and Alaska.** ON, QC, NB, NS, **PE** (Bousquet et al. 2013; Webster et al. 2022).

***Megapenthesrogersi* Horn, 1871**

**New record. Kings Co.**, Valleyfield, 46.1356°N, 62.7198°W, 3.VII–13.VIII.2019, canopy trap in poplar (1, AFC).

**Distribution in Canada and Alaska.** ON, QC, NB, NS, **PE** (Bousquet et al. 2013).

***Idolusdebilis* (LeConte, 1884)**

**New records. Kings Co.**, Valleyfield, 46.1356°N, 62.7198°W, 3.VII–2.VIII.2018 (2), 3.VII–13.VIII.2019 (1), canopy traps in hemlock (1), in red maple (1), low trap (1) (3, AFC). **Queens Co.**, Brookvale, 46.2920°N, 63.4051°W, 13.VI–3.VII.2018 (1), 3.VII–2.VIII.2018 (1), 3.VII–13.VIII.2019 (1), canopy trap in hemlock (1) low traps (2) (3, AFC).

**Distribution in Canada and Alaska.**AK, YT, BC, AB, SK, MB, ON, QC, NB, NS, **PE**, LB, NF (Bousquet et al. 2013).

#### Family LYCIDAE Laporte, 1836

Members of this family were not previously known from PE. Here we report eight species of this family from the province.

##### Subfamily DICTYOPTERINAE Houlbert, 1922

*Dictyopteraaurora* (Herbst, 1784)*

**New records. Kings Co.**, Valleyfield, 46.1356°N, 62.7198°W, 7.V–4.VI.2019, 4.VI–3.VII.2019, low trap (2, AFC); New Harmony, 46.3914°N, 62.2021°W, 8.V–5.VI.2019, low trap (1, AFC). **Queens Co.**, Brookvale, 46.2920°N, 63.4051°W, 13.VI–3.VII.2018, 7.V–4.VI.2019, low traps (2, AFC).

**Distribution in Canada and Alaska.**AK, YT, NT, BC, AB, SK, MB, ON, QC, NB, NS, **PE**, LB, NF (Bousquet et al. 2013).

##### Subfamily LYCINAE Laporte, 1836

***Caeniadimidiata* (Fabricius, 1801)**

**New record. Kings Co.**, Valleyfield, 46.1356°N, 62.7198°W, 3.VII–13.VIII.2019, canopy traps in poplar (1), in white spruce (1) (2, AFC).

**Distribution in Canada and Alaska.**MB, ON, QC, NB, NS, **PE** (Bousquet et al. 2013).

***Leptoceletesbasalis* (LeConte, 1847)**

**New records. Kings Co.**, Valleyfield, 46.1356°N, 62.7198°W, 3.VII–13.VIII.2019, low trap (1, AFC). **Queens Co.**, Auburn, 46.2882°N, 62.9267°W, 3.VII–2.VIII.2018, canopy trap in poplar (1, AFC); Brookvale, 46.2920°N, 63.4051°W, 3.VII–2.VIII.2018, low traps (4, AFC).

**Distribution in Canada and Alaska.**SK, MB, ON, QC, NB, NS, **PE** (Bousquet et al. 2013).

***Eropterusarculus* Green, 1951**

**New record. Kings Co.**, Valleyfield, 46.1356°N, 62.7198°W, 3.VII–13.VIII.2019, low trap (1, AFC).

**Distribution in Canada and Alaska.** ON, QC, NB, **PE** (Bousquet et al. 2013).

***Eroshumeralis* (Fabricius, 1801)**

**New record. Kings Co.**, Valleyfield, 46.1356°N, 62.7198°W, 2.VIII–13.IX.2018, low trap (1, AFC).

**Distribution in Canada and Alaska.** ON, QC, NB, NS, **PE** (Bousquet et al. 2013).

**Erotides (Erotides) sculptilis (Say, 1835)**

**New record. Kings Co.**, Valleyfield, 46.1356°N, 62.7198°W, 3.VII–13.VIII.2019, low trap (1, AFC).

**Distribution in Canada and Alaska.** ON, QC, NB, **PE** (Bousquet et al. 2013).

***Platerosflavoscutellatus* Blatchley, 1914**

**New record. Queens Co.**, Brookvale, 46.2920°N, 63.4051°W, 3.VII–2.VIII.2018, low trap (1, AFC).

**Distribution in Canada and Alaska.** ON, QC, NB, NS, **PE** (Bousquet et al. 2013).

***Plateroalictor* (Newman, 1838)**

**New records. Kings Co.**, Valleyfield, 46.1356°N, 62.7198°W, 3.VII–2.VIII.2018, canopy trap in red maple (1, AFC). **Queens Co.**, Auburn, 46.2882°N, 62.9267°W, 3.VII–2.VIII.2018, 3.VII–`4.VIII.2019, canopy traps in poplar (2, AFC); Brookvale, 46.2920°N, 63.4051°W, 13.VI–3.VII.2018, low trap (1, AFC).

**Distribution in Canada and Alaska.**BC, AB, SK, MB, ON, QC, NB, NS, **PE** (Bousquet et al. 2013).

#### Family LAMPYRIDAE Rafinesque, 1815

Bousquet et al. (2013) listed six species of Lampyridae from PE. In our study we recorded five species, one being a new record for the province.

##### Subfamily LAMPYRINAE Rafinesque, 1815

***Pyractomenaborealis* (Randall, 1838)**

**New record. Kings Co.**, Valleyfield, 46.1356°N, 62.7198°W, 12.VI–3.VII.2018 (2), 3.VII–2.VIII.2018 (1), 4.VI–3.VII.2019 (1), 3.VII–13.VIII.2019 (1), canopy traps in poplar (3) in hemlock (2), (5, AFC).

**Distribution in Canada and Alaska.**AB, SK, MB, ON, QC, NB, NS, **PE** (Bousquet et al. 2013).

#### Family CANTHARIDAE Imhoff, 1856

Only one species, the adventive *Cantharisrufa* Linnaeus was previously known from PE (Bousquet et al. 2013). Pelletier and Hébert (2014) did not provide any additional PE records in their treatment of this family. Pentinsaari et al. (2019) subsequently added the adventive *Malthodespumilus* (Brébisson) to the provincial list. Here, we provide 14 new records for PE, bringing the total number of species known to the province to 16.

##### Subfamily CANTHARINAE Imhoff, 1856

***Pacificanthiarotundicollis* (Say, 1825)**

**New records. Kings Co.**, Valleyfield, 46.1356°N, 62.7198°W, 12.VI–3.VII.2018 (1), 4.VI–3.VII.2019 (1), canopy traps in red maple (1), in white spruce (1) (2 AFC); New Harmony, 5.VI–3.VII.2019, low trap (1, AFC). **Queens Co.**, Auburn, 46.2882°N, 62.9267°W, 13.VI–3.VII.2018 (2), 3.VII–14.VIII.2019 (1), canopy traps in poplar (3, AFC); Brookvale, 46.2920°N, 63.4051°W, 3.VII–2.VIII.2018 (1), 3.VII–13.VIII.2019, low traps (2, AFC).

**Distribution in Canada and Alaska.** ON, QC, NB, NS, **PE** (Bousquet et al. 2013; Pelletier and Hébert 2014).

***Rhagonychafraxini* (Say, 1823)**

**New records. Kings Co.**, Valleyfield, 46.1356°N, 62.7198°W, 12.VI–3.VII.2018 (1), 3.VII–2.VIII.2018 (1), canopy trap in hemlock (1), low trap (1) (2 AFC). **Queens Co.**, Auburn, 46.2882°N, 62.9267°W, 13.VI–3.VII.2018, canopy traps in poplar (2, AFC); Brookvale, 46.2920°N, 63.4051°W, 13.VI–3.VII.2018 (4), 3.VII–2.VIII.2018 (1), low traps (4, AFC).

**Distribution in Canada and Alaska.**BC, AB, MB, ON, QC, NB, NS, **PE**, NF (Bousquet et al. 2013; Pelletier and Hébert 2014).

***Rhagonychaimbecillis* (LeConte, 1851)**

**New records. Kings Co.**, Valleyfield, 46.1356°N, 62.7198°W, 3.VII–2.VIII.2018, canopy trap in red maple (1 AFC); New Harmony, 46.3914°N, 62.2021°W, 3.VII–13.VIII.2019, canopy traps in red maple (3), sugar maple (1), white pine (2) (6, AFC).

**Distribution in Canada and Alaska.**MB, ON, QC, NB, **PE** (Bousquet et al. 2013; Pelletier and Hébert 2014).

***Rhagonycharecta* (Melsheimer, 1846)**

**New records. Kings Co.**, Valleyfield, 46.1356°N, 62.7198°W, 12.VI–3.VII.2018 (2), 3.VII–2.VIII.2018 (7), 4.VI–3.VII.2019 (2), 3.VII–13.VIII.2019 (3), canopy traps in red maple (3), in poplar (3), in hemlock (6), in white spruce (2) (14, AFC); New Harmony, 46.3914°N, 62.2021°W, 5.VI–3.VII.2019 (1), 3.VII–13.VIII.2019 (2), canopy traps in white pine (2), in sugar maple (1) (3, AFC). **Queens Co.**, Auburn, 46.2882°N, 62.9267°W, 3.VII–2.VIII.2018 (5), 4.VI–3.VII.2019 (1), 3.VII–14.VIII.2019 (2), canopy traps in poplar (7), low trap (1) (8, AFC); Brookvale, 46.2920°N, 63.4051°W, 3.VII–2.VIII.2018 (4), 4.VI–3.VII.2019 (2), 3.VII–13.VIII.2019 (1), low traps (7, AFC).

**Distribution in Canada and Alaska.**BC, AB, SK, MB, ON, QC, NB, NS, **PE**, NF (Bousquet et al. 2013; Pelletier and Hébert 2014).

***Dichelotarsuspiniphilus* (Eschscholtz, 1830)**

**New records. Kings Co.**, Valleyfield, 46.1356°N, 62.7198°W, 12.VI–3.VII.2018, canopy traps in red maple (1), in poplar (1) (2, AFC). **Queens Co.**, Auburn, 46.2882°N, 62.9267°W, 13.VI–3.VII.2018, canopy trap in red maple (1, AFC).

**Distribution in Canada and Alaska.**AK, YT, NT, NU, BC, AB, SK, MB, ON, QC, NB, NS, **PE**, NF (Bousquet et al. 2013; Pelletier and Hébert 2014).

***Dichelotarsuspuberulus* (LeConte, 1850)**

**New records. Kings Co.**, Valleyfield, 46.1356°N, 62.7198°W, 12.VI–3.VII.2018, canopy traps in red maples (2, AFC). **Queens Co.**, Auburn, 46.2882°N, 62.9267°W, 13.VI–3.VII.2018, canopy trap in poplar (1, AFC).

**Distribution in Canada and Alaska.**AB, SK, MB, ON, QC, NB, NS, **PE**, LB, NF (Bousquet et al. 2013; Pelletier and Hébert 2014).

***Dichelotarsuspunctatus* (LeConte, 1850)**

**New records. Kings Co.**, Valleyfield, 46.1356°N, 62.7198°W, 12.VI–3.VII.2018, canopy traps in red maple (1), in poplar (1) (2, AFC); New Harmony, 46.3914°N, 62.2021°W, 5.VI–3.VII.2019, canopy trap in red maple (1, AFC). **Queens Co.**, Brookvale, 46.2920°N, 63.4051°W, 13.VI–3.VII.2018, low trap (1, AFC).

**Distribution in Canada and Alaska.** ON, QC, NB, NS, **PE** (Bousquet et al. 2013; Pelletier and Hébert 2014).

***Dichelotarsussimplex* (Couper, 1865)**

**New records. Kings Co.**, Valleyfield, 46.1356°N, 62.7198°W, 12.VI–3.VII.2018, canopy trap in red maple (1, AFC). **Queens Co.**, Auburn, 46.2882°N, 62.9267°W, 13.VI–3.VII.2018, canopy trap in poplar (1, AFC).

**Distribution in Canada and Alaska.**MB, ON, QC, NB, NS, **PE** (Bousquet et al. 2013; Pelletier and Hébert 2014).

***Podabrusdiadema* (Fabricius, 1798)**

**New records. Kings Co.**, Valleyfield, 46.1356°N, 62.7198°W, 12.VI–3.VII.2018 (1), 4.VI–3.VII.2019 (2) canopy traps in red maple (1), in hemlock (1), white spruce (1), low traps (2) (5, AFC); New Harmony, 46.3914°N, 62.2021°W, 5.VI–3.VII.2019 (2), 3.VII–13.VIII.2019 (1), canopy traps in sugar maple (2), white pine (1) (3, AFC). **Queens Co.**, Auburn, 46.2882°N, 62.9267°W, 4.VI–3.VII.2019, canopy trap in poplar (1, AFC); Brookvale, 46.2920°N, 63.4051°W, 13.VI–3.VII.2018 (2), 3.VII–2.VIII.2018 (3), 4.VI–3.VII.2019 (1), low traps (6, AFC).

**Distribution in Canada and Alaska.**AB, ON, QC, NB, NS, **PE** (Bousquet et al. 2013; Pelletier and Hébert 2014).

***Podabrusintrusus* Green, 1947**

**New record. Queens Co.**, Auburn, 46.2882°N, 62.9267°W, 3.VII–2.VIII.2018, canopy trap in poplar snag (1, AFC).

**Distribution in Canada and Alaska.** ON, QC, NB, NS, **PE**, NF (Bousquet et al. 2013; Pelletier and Hébert 2014).

***Podabrusmodestus* (Say, 1823)**

**New record. Kings Co.**, Valleyfield, 46.1356°N, 62.7198°W, 3.VII–2.VIII.2018 (1), 4.VI–3.VII.2019 (1), 3.VII–13.VII.2019 (1), canopy traps in red maple (1), in white spruce (2) (3, AFC).

**Distribution in Canada and Alaska.** ON, QC, NB, NS, **PE**, NF (Bousquet et al. 2013; Pelletier and Hébert 2014).

##### Subfamily SILINAE Mulsant, 1862

***Silispercomis* (Say, 1835)**

**New records. Kings Co.**, Valleyfield, 46.1356°N, 62.7198°W, 12.VI–3.VII.2018 (2), 4.VI–3.VII.2019 (2), canopy traps in red maple (2), poplar (1), in white spruce (1) (4, AFC); New Harmony, 46.3914°N, 62.2021°W, 5.VI–3.VII.2019, canopy trap in white pine (1, AFC). **Queens Co.**, Auburn, 46.2882°N, 62.9267°W, 13.VI–3.VII.2018 (1), 4.VI–3.VII.2019 (1), canopy traps in poplar (2, AFC); Brookvale, 46.2920°N, 63.4051°W, 13.VI–3.VII.2018, low traps (2, AFC).

**Distribution in Canada and Alaska.** ON, QC, NB, NS, **PE** (Bousquet et al. 2013; Pelletier and Hébert 2014).

##### Subfamily MALTHININAE Kiesenwetter, 1852

***Malthodesfragilis* (LeConte, 1851)**

**New record. Kings Co.**, New Harmony, 46.3914°N, 62.2021°W, 3.VII–13.VIII.2019, canopy traps in red maple (1, AFC; 2, RWC).

**Distribution in Canada and Alaska.** YK, ON, QC, NB, NS, **PE** (Bousquet et al. 2013; Pelletier and Hébert 2014).

***Malthodesniger* (LeConte, 1851)**

**New record. Queens Co.**, Brookvale, 46.2920°N, 63.4051°W, 3.VII–2.VIII.2018, low trap (1, RWC).

**Distribution in Canada and Alaska.** ON, QC, NB, NS, **PE** (Bousquet et al. 2013; Pelletier and Hébert 2014).

#### Family DERODONTIDAE LeConte, 1861

The records below represent the first report of this beetle family for PE. Two species are known from adjacent NB, and four are known from NS (Bousquet et al. 2013).

##### Subfamily LARICOBIINAE Mulsant & Rey, 1864

***Laricobiusrubidus* LeConte, 1861**

**New records. Queens Co.**, Auburn, 46.2882°N, 62.9267°W, 7.V–4.VI.2019, canopy trap in poplar (1, AFC); Brookvale, 46.2920°N, 63.4051°W, 7.V–4.VI.2019, canopy trap in hemlock (1, AFC).

**Distribution in Canada and Alaska.** ON, QC, NB, NS, **PE** (Bousquet et al. 2013).

#### Family DERMESTIDAE Latreille, 1804

Eleven species are known from PE (Bousquet et al. 2013). In this study we recorded only one of these species, the adventive *Anthrenusmuseorum* (Linnaeus).

#### Family BOSTRICHIDAE Latreille, 1802

Five species are known from PE (Bousquet et al. 2013). We did not record any of these species in our study.

#### Family PTINIDAE Latreille, 1892

Bousquet et al. (2013) recorded fifteen species of Ptinidae from PE. Eleven species were recorded during this study, six of which are new to PE. This brings the total number of species known from the province to 21.

##### Subfamily ANOBIINAE Fleming, 1821

***Oligomerusobtusus* LeConte, 1865**

**New records. Kings Co.**, Valleyfield, 46.1356°N, 62.7198°W, 3.VII–2.VIII.2018, canopy trap in red maple (1, AFC).

**Distribution in Canada and Alaska.** ON, QC, NB, **PE** (Bousquet et al. 2013; Webster et al. 2016e).

##### Subfamily PTILININAE Shuckard, 1839

***Ptilinuslobatus* Casey, 1898**

**New record. Queens Co.**, Brookvale, 46.2920°N, 63.4051°W, 3.VII–2.VIII.2018, 3.VII–13.VIII.2019, canopy trap in white pine (1), low trap (1, AFC; 1, RWC).

**Distribution in Canada and Alaska.**AK, YT, BC, AB, MB, ON, NB, NS, **PE** (Bousquet et al. 2013).

***Ptilinuspruinosus* Casey, 1898**

**New records. Kings Co.**, Valleyfield, 46.1356°N, 62.7198°W, 3.VII–13.VIII.2019, canopy trap in poplar (1), in white spruce (1) (2, AFC). **Queens Co.**, Auburn, 46.2882°N, 62.9267°W, 3.VII–2.VIII.2018, 3.VII–14.VIII.2019, canopy trap in red maple (1), low trap (1) (2, AFC).

**Distribution in Canada and Alaska.**MB, ON, QC, NB, NS, **PE** (Bousquet et al. 2013; Webster et al. 2016e).

***Ptilinusruficornis* Say, 1823**

**New records. Kings Co.**, Valleyfield, 46.1356°N, 62.7198°W, 3.VII–2.VIII.2018, 3.VII–13.VIII.2019, canopy traps in red maple (1), in poplar (1) (2, AFC); New Harmony, 46.3914°N, 62.2021°W, 3.VII–13.VIII.2019, canopy trap in sugar maple (1, AFC). **Queens Co.**, Auburn, 46.2882°N, 62.9267°W, 13.VI–3.VII.2018 (1), 3.VII–2.VIII.2018 (2), canopy traps in poplar (3, AFC).

**Distribution in Canada and Alaska.**AB, SK, ON, QC, NB, NS, **PE** (Bousquet et al. 2013).

##### Subfamily XYLETININAE Gistel, 1848

***Xyletinuslugubris* LeConte, 1878**

**New record. Queens Co.**, Auburn, 46.2882°N, 62.9267°W, 3.VII–2.VIII.2018, canopy trap in poplar (1, AFC).

**Distribution in Canada and Alaska.**AB, SK, ON, QC, NB, NS, **PE** (Bousquet et al. 2013; Webster et al. 2016e).

##### Subfamily DORCATOMINAE C.G. Thomson, 1859

***Sculptothecapuberula* (LeConte, 1865)**

**New record. Kings Co.**, Valleyfield, 46.1356°N, 62.7198°W, 3.VII–13.VIII.2019, low trap (1, AFC).

**Distribution in Canada and Alaska.** ON, QC, NB, NS, **PE** (Bousquet et al. 2013; Webster et al. 2016e).

#### Family LYMEXYLIDAE Fleming, 1821

This is the first record of this family for the province of PE. *Elateroideslugubris* (Say) is the only member of this family known from Canada (Bousquet et al. 2013).

##### Subfamily HYLECOETINAE Germar, 1818

***Elateroideslugubris* (Say, 1835)**

**New records. Kings Co.**, Valleyfield, 46.1356°N, 62.7198°W, 7.V–4.VI.2019, 4.VI–3.VII.2019, low traps (2, AFC). **Queens Co.**, Auburn, 46.2882°N, 62.9267°W, 7.V–4.VI.2019, low trap (1, AFC).

**Distribution in Canada and Alaska.**AB, MB, ON, QC, NB, NS, **PE**, NF (Bousquet et al. 2013).

#### Family TROGOSSITIDAE Latreille, 1802

Bousquet et al. (2013 reported two members of this family for PE. In this study three species were found at the four study sites, two of which are new for PE.

##### Subfamily TROGOSSITINAE Latrielle, 1802

*Calitysscabra* (Thunberg, 1784)*

**New record. Kings Co.**, Valleyfield, 46.1356°N, 62.7198°W, 4.VI–3.VII.2019, low traps (2, AFC).

**Distribution in Canada and Alaska.**AK, NT, BC, AB, SK, ON, QC, NB, NS, **PE** (Bousquet et al. 2013).

***Tenebriodescorticalis* (Melsheimer, 1844)**

**New record. Kings Co.**, Valleyfield, 46.1356°N, 62.7198°W, 12.VI–3.VII.2018 (1), 4.VI–3.VIII.2019 (1), 3.VII–13.VIII.2019 (1), canopy traps in poplar (2) and in hemlock (1) (3, AFC).

**Distribution in Canada and Alaska.**AK, YT, NT, BC, SK, MB, ON, QC, NB, NS, **PE** (Bousquet et al. 2013).

#### Family CLERIDAE Latreille, 1802

Eight species of Cleridae were recorded from the four woodlots, five being new records. Bousquet et al. (2013) listed seven species of Cleridae for PE. With these new records there are currently 12 species known from PE.

##### Subfamily TILLINAE Fischer von Waldheim, 1813

***Cymatoderabicolor* (Say, 1825)**

**New records. Kings Co.**, Valleyfield, 46.1356°N, 62.7198°W, 3.VII–13.VIII.2019, canopy trap in poplar (1, AFC). **Queens Co.**, Auburn, 46.2882°N, 62.9267°W, 3.VII–2.VIII.2018 (2), 3.VII–14.VIII.2019 (1) canopy traps in red maple (2), in poplar (1) (3, AFC).

**Distribution in Canada and Alaska.**MB, ON, QC, NB, NS, **PE** (Bousquet et al. 2013).

##### Subfamily CLERINAE Latreille, 1802

***Enoclerusnigripesrufiventris* (Spinola, 1844)**

**New records. Kings Co.**, Valleyfield, 46.1356°N, 62.7198°W, 12.VI–3.VII.2018 (2), 3.VII–2.VIII.2018 (1), 7.V–4.VI.2019 (1), canopy traps in poplar (1), in hemlock (3) (4, AFC). **Queens Co.**, Auburn, 46.2882°N, 62.9267°W, 13.VI–3.VII.2018 (1), 3.VII–2.VIII.2018 (1), 4.VI–3.VII.2019 (1), canopy traps in poplar (2), low trap (1) (3, AFC).

**Distribution in Canada and Alaska.** ON, QC, NB, NS, **PE** (Bousquet et al. 2013).

***Thanasimusdubius* (Fabricius, 1777)**

**New records. Kings Co.**, Valleyfield, 46.1356°N, 62.7198°W, 12.VI–3.VII.2018, 4.VI–3.VII.2019, canopy trap in white spruce (1) low trap (1) (2, AFC); New Harmony, 46.3914°N, 62.2021°W, 5.VI–3.VII.2019, canopy trap in red maple (1, AFC). **Queens Co.**, Auburn, 46.2882°N, 62.9267°W, 13.VI–3.VII.2018, 4.VI–3.VII.2019, low traps (2, AFC); Brookvale, 46.2920°N, 63.4051°W, 13.VI–3.VII.2018, 7.V–4.VI.2019, low traps (2, AFC).

**Distribution in Canada and Alaska.**AK, YT, NT, BC, AB, AK, MB, ON, QC, NB, NS, **PE**, NF (Bousquet et al. 2013).

***Thanasimusundatulusnubilus* (Klug, 1842)**

**New record. Queens Co.**, Auburn, 46.2882°N, 62.9267°W, 13.VI–3.VII.2018 (1), 4.VI–3.VII.2019, 3.VII–14.VIII.2019 (1), canopy traps in poplar (2), in red maple (1) (3, AFC).

**Distribution in Canada and Alaska.**YT, NT, BC, AB, MB, ON, QC, NB, NS, **PE**, NF (Bousquet et al. 2013).

***Thanasimusundatulusundatulus* (Say, 1835)**

**New records. Kings Co.**, Valleyfield, 46.1356°N, 62.7198°W, 12.VI–3.VII.2018 (2), 4.VI–3.VII.2019 (1), canopy trap in white spruce (1), low traps (2) (3, AFC). **Queens Co.**, Auburn, 46.2882°N, 62.9267°W, 13.VI–3.VII.2018 (2), 4.VI–3.VII.2019 (1), canopy trap in poplar (1), low traps (2) (3, AFC); Brookvale, 46.2920°N, 63.4051°W, 13.VI–3.VII.2018 (2), 7.V–4.VI.2019 (1), canopy trap in white pine (1), low traps (2) (3, AFC).

**Distribution in Canada and Alaska.**AK, NT, BC, AB, ON, QC, NB, NS, **PE** (Bousquet et al. 2013; Webster et al. 2016e).

**Comments.**Webster et al. (2016e) commented that these two subspecies co-occur at many sites in NB without any intermediate specimens. The same situation occurs in PE. More studies are required to establish the status of these two co-occurring subspecies.

#### Family MELYRIDAE Leach, 1815

Only one species of Melyridae, the adventive *Malachiusaeneus* (Linnaeus), was previously known from PE (Bousquet et al. (2013). Here we report another species new to the province.

##### Subfamily MALACHIINAE Fleming, 1821

***Nodopusflavilabris* (Say, 1825)**

**New record. Queens Co.**, Brookvale, 46.2920°N, 63.4051°W, 13.VI–3.VIII.2018, low trap (1, AFC).

**Distribution in Canada and Alaska.** ON, QC, NB, **PE** (Bousquet et al. 2013).

#### Family BYTURIDAE Gistel, 1848

*Byturusunicolor* Say is the only member of this family in Canada and was previously recorded from PE by Bousquet et al. (2013). We found this species at three of the four study sites.

#### Family SPHINDIDAE Jacquelin du Val, 1860

These are the first records of this family for the province of PE. Only four species are known from the Maritime Provinces (Bousquet et al. 2013).

##### Subfamily ODONTOSPHINDINAE Sen Gupta & Crowson, 1979

***Odontosphindusdenticollis* LeConte, 1878**

**New records. Kings Co.**, Valleyfield, 46.1356°N, 62.7198°W, 3.VII–2.VIII.2018, low traps (3, AFC). **Queens Co.**, Auburn, 46.2882°N, 62.9267°W, 3.VII–2.VIII.2018 (2), 2.VIII–13.IX.2018 (2), canopy traps in poplar (4, AFC); Brookvale, 46.2920°N, 63.4051°W, 2.VIII–13.IX.2018, low trap (1, AFC).

**Distribution in Canada and Alaska.**MB, ON, QC, NB, NS, **PE** (Bousquet et al. 2013).

##### Subfamily SPHINDINAE Jacquelin du Val, 1860

***Sphindusamericanus* LeConte, 1866**

**New record. Kings Co.**, Valleyfield, 46.1356°N, 62.7198°W, 4.VI–3.VII.2019, low trap (1, AFC).

**Distribution in Canada and Alaska.**BC, AB, MB, ON, QC, NB, **PE** (Bousquet et al. 2013).

***Sphindustrinifer* Casey, 1898**

**New records. Kings Co.**, Valleyfield, 46.1356°N, 62.7198°W, 4.VI–3.VII.2019 (2), 3.VII–13.VIII.2019 (1), low traps (3, AFC). **Queens Co.**, Auburn, 46.2882°N, 62.9267°W, 2.VIII–13.IX.2018, 4.VI–3.VIII.2019, low traps (2, AFC); Brookvale, 46.2920°N, 63.4051°W, 13.VI–3.VII.2018, low trap (1, AFC).

**Distribution in Canada and Alaska.**MB, ON, QC, NB, NS, **PE** (Bousquet et al. 2013).

#### Family EROTYLIDAE Latreille, 1802

Two species of this family were previously known from PE (Bousquet et al. 2013). Here we report another two species. Both known PE species were also recorded in this study.

##### Subfamily EROTYLINAE Latreille, 1802

***Triplaxfrosti* Casey, 1824**

**New records. Kings Co.**, Valleyfield, 46.1356°N, 62.7198°W, 12.VI–3.VII.2018 (1), 3.VII–2.VIII.2018 (2), 4.VI–3.VII.2019 (1), 3.VII–13.VIII.2019 (1), canopy traps in red maple (1), in poplar (1), in white spruce (1), in hemlock (1), low trap (1) (5, AFC). **Queens Co.**, Auburn, 46.2882°N, 62.9267°W, 13.VI–3.VII.2018, canopy trap in poplar (1, AFC); Brookvale, 46.2920°N, 63.4051°W, 13.VI–3.VII.2018, low trap (1, AFC).

**Distribution in Canada and Alaska.**AB, SK, MB, ON, QC, NB, NS, **PE** (Bousquet et al. 2013).

***Tritomapulchra* Say, 1826**

**New record. Kings Co.**, Valleyfield, 46.1356°N, 62.7198°W, 3.VII–2.VIII.2018 (1), 4.VI–3.VIII.2019 (1), low traps (3, AFC).

**Distribution in Canada and Alaska.**MB, ON, QC, NB, NS, **PE** (Bousquet et al. 2013).

#### Family MONOTOMIDAE Laporte, 1840

Four species of Monotomidae were recorded from the four woodlots; three are new records for PE. Two species of this family were previously known from PE (Bousquet et al. 2013).

##### Subfamily RHIZOPHAGINAE Redtenbacher, 1845

**Rhizophagus (Anomophagus) brunneus brunneus Horn, 1879**

**New records. Kings Co.**, Valleyfield, 46.1356°N, 62.7198°W, 4.VI–3.VIII.2019, low trap (1, AFC). **Queens Co.**, Auburn, 46.2882°N, 62.9267°W, 13.VI–3.VII.2018, low traps (2, AFC).

**Distribution in Canada and Alaska.**AB, ON, QC, NB, NS, **PE** (Bousquet et al. 2013).

**Rhizophagus (Rhizophagus) dimidiatus Mannerheim, 1843**

**New records. Kings Co.**, Valleyfield, 46.1356°N, 62.7198°W, 12.VI–3.VII.2018 (1), 3.VII–2.VIII.2018 (1), 4.VI–3.VII.2019 (1), low traps (3, AFC); New Harmony, 46.3914°N, 62.2021°W, 5.VI–3.VII.2019, low trap (1, AFC). **Queens Co.**, Brookvale, 46.2920°N, 63.4051°W, 13.VI–3.VII.2018 (1), 3.VII–2.VIII.2018 (1), 4.VI–3.VII.2019 (1), low traps (3, AFC).

**Distribution in Canada and Alaska.**AK, YT, BC, AB, SK, ON, QC, NB, NS, **PE**, NF (Bousquet et al. 2013).

**Rhizophagus (Rhizophagus) remotus LeConte, 1866**

**New records. Kings Co.**, Valleyfield, 46.1356°N, 62.7198°W, 7.V–4.VI.2019, low trap (1, AFC). **Queens Co.**, Auburn, 46.2882°N, 62.9267°W, 13.VI–3.VII.2018, 7.V–4.VI.2019, low traps (2, AFC); Brookvale, 46.2920°N, 63.4051°W, 13.VI–3.VII.2018, low trap (1, AFC).

**Distribution in Canada and Alaska.**AK, BC, AB, SK, MB, ON, QC, NB, NS, **PE** (Bousquet et al. 2013).

#### Family CRYPTOPHAGIDAE Kirby, 1826

Ten species of Cryptophagidae were found at the four woodlots in this study. Among these six are new to PE. Seven species were previously known from the province (Bousquet et al. 2013). These new additions bring the total number species of this family known from PE to 13.

##### Subfamily CRYPTOPHAGINAE Kirby, 1826

*Henotideruscentromaculatus* Reitter, 1877*

**New record. Queens Co.**, Auburn, 46.2882°N, 62.9267°W, 13.VI–3.VII.2018, canopy trap in poplar (1, AFC).

**Distribution in Canada and Alaska.**AK, NT, AB, SK, ON, QC, NB, NS, **PE** (Bousquet et al. 2013).

##### Subfamily ATOMARIINAE LeConte, 1861

**Atomaria (Anchicera) lewisi Reitter, 1877**†

**New record. Queens Co.**, Brookvale, 46.2920°N, 63.4051°W, 13.VI–3.VII.2018, low trap (1, AFC).

**Distribution in Canada and Alaska.** ON, QC, NB, NS, **PE** (Bousquet et al. 2013).

Atomaria (Atomaria) affinis R.F. Sahlberg, 1834*

**New record. Kings Co.**, New Harmony, 46.3914°N, 62.2021°W, 5.VI–3.VII.2019, low trap (1, AFC).

**Distribution in Canada and Alaska.**AK, BC, AB, QC, NB, NS, **PE** (Pelletier and Hébert 2019).

**Atomaria (Atomaria) alpina Heer, 1841**†

**New record. Queens Co.**, Brookvale, 46.2920°N, 63.4051°W, 4.VI–3.VII.2019, low trap (1, AFC).

**Distribution in Canada and Alaska.**BC, AB, ON, QC, NB, NS, **PE** (Pelletier and Hébert 2019).

**Atomaria (Atomaria) constricta (Casey, 1900)**

**New record. Queens Co.**, Brookvale, 46.2920°N, 63.4051°W, 13.VI–3.VII.2018, low trap (1, RWC).

**Distribution in Canada and Alaska.**BC, AB, QC, NB, **PE** (Pelletier and Hébert 2019).

**Atomaria (Atomaria) pinicola Pelletier, 2019**

**New record. Kings Co.**, Valleyfield, 46.1356°N, 62.7198°W, 4.VI–3.VII.2019, low trap (1, AFC).

**Distribution in Canada and Alaska.**QC, NB, **PE** (Pelletier and Hébert 2019).

#### Family SILVANIDAE Kirby, 1837

Two species of Silvanidae were recorded from the four woodlots. Both are new records for PE. Two other species were previously known from PE (Bousquet et al. 2013).

##### Subfamily BRONTINAE Blanchard, 1845

***Dendrophaguscygnaei* Mannerheim, 1846**

**New records. Kings Co.**, Valleyfield, 46.1356°N, 62.7198°W, 7.V–4.VI.2019, low trap (1, AFC); New Harmony, 46.3914°N, 62.2021°W, 5.VI–3.VII.2019, low trap (1, AFC). **Queens Co.**, Brookvale, 46.2920°N, 63.4051°W, 13.VI–3.VII.2018, 7.V–4.VI.2019, low traps (2, AFC).

**Distribution in Canada and Alaska.**AK, BC, AB, SK, MB, ON, QC, NB, NS, **PE**, NF (Bousquet et al. 2013).

##### Subfamily SILVANINAE Kirby, 1837

***Silvanusbidentatus* (Fabricius, 1792)**†

**New records. Kings Co.**, Valleyfield, 46.1356°N, 62.7198°W, 12.VI–3.VII.2018, low traps (1, AFC); New Harmony, 46.3914°N, 62.2021°W, 8.V–5.VI.2019, low trap (1, AFC). **Queens Co.**, Auburn, 46.2882°N, 62.9267°W, 13.VI–3.VII.2018, low trap (1, AFC); Brookvale, 46.2920°N, 63.4051°W, 13.VI–3.VII.2018 (2), 4.VI–3.VII.2019 (1), low traps (3, AFC).

**Distribution in Canada and Alaska.**BC, ON, QC, NB, NS, **PE** (Bousquet et al. 2013).

#### Family CUCUJIDAE Latreille, 1802

This represents the first record of this family for PE. Only two species of this family occur in the Maritime Provinces (Bousquet et al. 2013).

***Cucujusclavipesclavipes* Fabricius, 1777**

**New records. Kings Co.**, Valleyfield, 46.1356°N, 62.7198°W, 3.VII–2.VIII.2018 (3), 7.V–4.VI.2019 (1), low traps (4, AFC); New Harmony, 46.3914°N, 62.2021°W, 5.VI–3.VII.2019, low trap (1, AFC). **Queens Co.**, Auburn, 46.2882°N, 62.9267°W, 13.VI–3.VII.2018, 7.V–4.VI.2019, low traps (2, AFC).

**Distribution in Canada and Alaska.**MB, ON, QC, NB, NS, **PE**, NF (Bousquet et al. 2013).

#### Family PHALACRIDAE Leach, 1815

One (*Olibrussemistriatus*, LeConte) of the two known species of this family reported by Bousquet et al. (2013) from PE was found at the Valleyfield Woodlot.

#### Family LAEMOPHLOEIDAE Ganglbauer, 1899

Five species of Laemophloeidae were recorded from the four woodlots; three of these are new to PE. Bousquet et al. (2013) previously reported the other two species and thus all known species from PE were detected in this study using Lindgren funnel traps.

***Charaphloeusconvexulus* (LeConte, 1879)**

**New records. Kings Co.**, Valleyfield, 46.1356°N, 62.7198°W, 12.VI–3.VII.2018, 7.V–4.VI.2019, low trap (1), canopy trap (1) (2, AFC); New Harmony, 46.3914°N, 62.2021°W, 8.V–5.VI.2019, canopy trap (1, AFC).

**Distribution in Canada and Alaska.** ON, QC, NB, NS, **PE** (Bousquet et al. 2013).

***Cryptolestesturicicus* (Grouvelle, 1876)**†

**New records. Kings Co.**, Valleyfield, 46.1356°N, 62.7198°W, 3.VII–2.VIII.2018, 3.VII–13.VIII.2019, canopy traps in red maple (1), in poplar (1) (2, AFC). **Queens Co.**, Auburn, 46.2882°N, 62.9267°W, 3.VII–14.VII.2019, canopy trap in poplar (1), low trap (1) (2 AFC).

**Distribution in Canada and Alaska.**BC, AB, SK, MB, ON, QC, NB, NS, **PE** (Bousquet et al. 2013; Webster et al. 2016e).

***Laemophloeusfasciatus* Melsheimer, 1844**

**New records. Kings Co.**, Valleyfield, 46.1356°N, 62.7198°W, 12.VI–3.VII.2018 (1), 2.VIII–13.IX.2018 (1), 3.VII–13.VIII.2019 (1), low traps (3, AFC); New Harmony, 46.3914°N, 62.2021°W, 5.VI–3.VII.2019, low trap (1, AFC). **Queens Co.**, Auburn, 46.2882°N, 62.9267°W, 13.VI–3.VII.2018 (1), 3.VII–2.VIII.2018 (2), low traps (3, AFC); Brookvale, 46.2920°N, 63.4051°W, 13.VI–3.VII.2018 (2), 3.VII–2.VIII.2018 (1), 2.VIII–13.IX.2018 (1), 7.V–4.VI.20–19 (1) low traps (4), canopy trap (1) (5, AFC).

**Distribution in Canada and Alaska.**SK, MB, ON, QC, NB, NS, **PE** (Bousquet et al. 2013).

#### Family KATERETIDAE Kirby, 1837

Bousquet et al. (2013) listed only the adventive *Brachypteroluspulicarius* (Linnaeus) from PE. Here we report two additional species.

***Brachypterusurticae* (Fabricius, 1792)**†

**New record. Queens Co.**, Brookvale, 46.2920°N, 63.4051°W, 4.VI–3.VII.2019, low trap (1, AFC).

**Distribution in Canada and Alaska.**AK, NT, BC, AB, SK, MB, ON, QC, NB, NS, **PE**, NF (Bousquet et al. 2013).

***Heterhelussericanssericans* (LeConte, 1859)**

**New records. Queens Co.**, Auburn, 46.2882°N, 62.9267°W, 13.VI–3.VII.2018, 7.V–4.VI.2019, canopy trap in poplar (1), low traps (2) (3, AFC); Brookvale, 46.2920°N, 63.4051°W, 7.V–4.VI.2019, low trap (1, AFC).

**Distribution in Canada and Alaska.**BC, ON, QC, NB, NS, **PE**, NF (Bousquet et al. 2013).

#### Family NITIDULIDAE Latreille, 1802

Bousquet et al. (2013) listed twelve species of Nitidulidae for PE. In this study, twenty-two species were captured in Lindgren funnel traps at the four woodlots. Thirteen of these are new to PE, including *Pityophagusferrugineus* (Linnaeus, 1760) which is a new North American record. Only two species previously known from PE were not captured in these traps.

##### Subfamily EPURAEINAE Kirejtshuk, 1986

Epuraea (Epuraea) linearis Mäklin, 1853*

**New records. Kings Co.**, Valleyfield, 46.1356°N, 62.7198°W, 2.VIII–13.IX.2018, low trap (1, AFC); New Harmony, 46.3914°N, 62.2021°W, 8.V–5.VI.2019, 5.VI–3.VII.2019, low traps (2, AFC). **Queens Co.**, Brookvale, 46.2920°N, 63.4051°W, 7.V–4.VI.2019, 3.VI–13.VII.2019, low traps (2, AFC).

**Distribution in Canada and Alaska.**AK, YT, NT, BC, AB, QC, NB, **PE** (Bousquet et al. 2013; Webster et al. 2016e).

**Epuraea (Epuraea) pallescens labilis Erichson, 1843**

**New records. Queens Co.**, Brookvale, 46.2920°N, 63.4051°W, 3.VII–13.VIII.2019, low trap (1, AFC).

**Distribution in Canada and Alaska.**AK, BC, AB, SK, MB, ON, QC, NB, NS, **PE**, NF (Bousquet et al. 2013).

**Epuraea (Epuraea) planulata Erichson, 1843**

**New records. Kings Co.**, New Harmony, 46.3914°N, 62.2021°W, 5.VI–3.VII.2019, 3.VII–13.VIII.2019, low traps (2, AFC). **Queens Co.**, Brookvale, 46.2920°N, 63.4051°W, 3.VI–13.VII.2019, low trap (1, AFC).

**Distribution in Canada and Alaska.**AK, BC, AB, SK, MB, ON, QC, NB, NS, **PE** (Bousquet et al. 2013).

Epuraea (Epuraea) rufomarginata (Stephens, 1830)*

**New records. Kings Co.**, Valleyfield, 46.1356°N, 62.7198°W, 7.V–4.VI.2019, 4.VI–3.VII.2019, low traps (2, AFC); New Harmony, 46.3914°N, 62.2021°W, 5.VI–3.VII.2019, 3.VII–13.VIII.2019, canopy trap in sugar maple, low trap (2, AFC). **Queens Co.**, Auburn, 46.2882°N, 62.9267°W, 4.VI–3.VII.2019, low trap (1, AFC); Brookvale, 46.2920°N, 63.4051°W, 7.V–4.VI.2019 (2), 3.VI–13.VII.2019 (1), low traps (3, AFC).

**Distribution in Canada and Alaska.**BC, MB, ON, QC, NB, NS, **PE** (Bousquet et al. 2013).

**Epuraea (Epuraea) truncatella (Mannerheim, 1846)**

**New records. Queens Co.**, Auburn, 46.2882°N, 62.9267°W, 7.V–4.VI.2019, low trap (1, AFC); Brookvale, 46.2920°N, 63.4051°W, 13.VI–3.VII.2018, 7.V–4.VI.2019, low traps (2, AFC).

**Distribution in Canada and Alaska.**AK, YT, NT, BC, AB, SK, MB, ON, QC, NB, NS, **PE**, LB, NF (Bousquet et al. 2013).

***Epuraeaavara* (Randall, 1838)**

**New records. Kings Co.**, Valleyfield, 46.1356°N, 62.7198°W, 12.VI–3.VII.2018, low trap (1, AFC). **Queens Co.**, Auburn, 46.2882°N, 62.9267°W, 13.VI–3.VII.2018 (1), 3.VII–2.VIII.2018 (1), 4.VI–3.VII.2019 (1), canopy trap in poplar (1), low traps (2) (3, AFC); Brookvale, 46.2920°N, 63.4051°W, 13.VI–3.VII.2018 (8), 3.VII–2.VIII.2018 (2), 4.VI–3.VII.2019 (2), 3.VII–13.VIII.2019 (1), canopy trap in white pine (1), low traps (12) (13, AFC).

**Distribution in Canada and Alaska.**AK, YT, BC, AB, MB, ON, QC, NB, NS, **PE**, NF (Bousquet et al. 2013).

##### Subfamily CARPOPHILINAE Erichson, 1842

**Carpophilus (Ecnomorphus) brachypterus (Say, 1825)**

**New record. Kings Co.**, Valleyfield, 46.1356°N, 62.7198°W, 7.V–4.VI.2019, low trap (1, AFC).

**Distribution in Canada and Alaska.**MB, ON, QC, NB, NS, **PE** (Bousquet et al. 2013).

##### Subfamily NITIDULINAE Latreille, 1802

***Omositanearctica* Kirejtshuk, 1987**

**New record. Queens Co.**, Brookvale, 46.2920°N, 63.4051°W, 4.VI–3.VII.2019, low trap (1, AFC).

**Distribution in Canada and Alaska.** ON, QC, NB, NS, **PE** (Bousquet et al. 2013).

***Cychramusadustus* Erichson, 1843**

**New record. Queens Co.**, Brookvale, 46.2920°N, 63.4051°W, 3.VII–2.VIII.2018, 3.VII–13.VIII.2019, low traps (2, AFC).

**Distribution in Canada and Alaska.** ON, QC, NB, **PE** (Bousquet et al. 2013).

##### Subfamily CILLAEINAE Kirejtshuk & Audisio, 1986

***Colopterustruncatus* (Randall, 1838)**

**New record. Kings Co.**, Valleyfield, 46.1356°N, 62.7198°W, 12.VI–3.VII.2018, 13.VIII–17.IX.2019, canopy traps in red maple (1), in poplar (1) (2, AFC).

**Distribution in Canada and Alaska.**YT, BC, AB, SK, MB, ON, QC, NB, NS, **PE** (Bousquet et al. 2013).

##### Subfamily CRYPTARCHINAE C.G. Thomson, 1859

**Glischrochilus (Glischrochilus) moratus W.J. Brown, 1932**

**New records. Kings Co.**, Valleyfield, 46.1356°N, 62.7198°W, 4.VI–3.VII.2018, canopy trap in white spruce (1, AFC). **Queens Co.**, Auburn, 46.2882°N, 62.9267°W, 7.V–4.VI.2019, canopy trap in poplar (1, AFC)

**Distribution in Canada and Alaska.**BC, AB, SK, MB, ON, QC, NB, NS, **PE** (Bousquet et al. 2013).

**Glischrochilus (Glischrochilus) vittatus (Say, 1835)**

**New record. Queens Co.**, Brookvale, 46.2920°N, 63.4051°W, 7.V–4.VI.2019, low trap (1, AFC).

**Distribution in Canada and Alaska.**AK, BC, AB, MB, ON, QC, NB, NS, **PE** (Bousquet et al. 2013).

***Pityophagusferrugineus* (Linnaeus, 1760)**†

**New record. Queens Co.**, Brookvale, 46.2920°N, 63.4051°W, 13.VI–3.VII.2018 (1), 3.VII–2.VIII.2018 (1), 2.VI–3.VII.2019 (2), low traps (1, AFC; 1, CNC, 2, RWC).

**Distribution in Canada and Alaska. PE**, New Canadian and North American Record.

**Figure 2.**
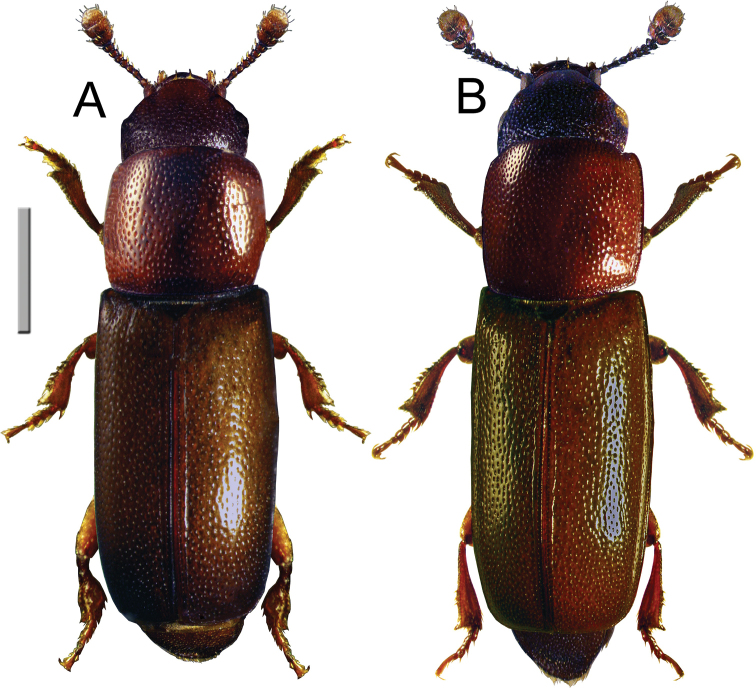
*Pityophagusferrugineus* (Linnaeus) **A** habitus in dorsal view of female from Brookvale, Queens Co., PE, Canada **B** habitus in dorsal view of female from Mögstorp, Östergötland Prov., Sweden. Scale bar: 1 mm.

**Comments**: Specimens (all females) of an unusual *Pityophagus* species unlike any North American species were captured at the Brookvale Woodlot (Fig. 2A). After checking various websites showing Palaearctic *Pityophagus* species, it appeared that this species was likely the European *P.ferrugineus.* We were able to confirm this identification by comparing our specimens with specimens (six females, one male) from Sweden and Finland (one female from Sweden is illustrated in Fig. 2B); the specimens were nearly identical in morphology, including the shape of the female ovipositor. In view of this, we conclude that the PE specimens are *P.ferrugineus*, which is a new North American and Canadian record.

*Pityophagusferrugineus* likely arrived in North America via untreated wood packaging of goods imported from Eurasia. Larvae of *P.ferrugineus* are common in galleries of coniferous bark beetles in Eurasia where they prey on larvae of a number of Scolytinae genera, including *Ips*, *Hylastes*, and *Dryocoetes* (Anon 2022). Scolytinae were the most common subfamily of beetles in wood packaging intercepted at US ports of entry between 1985 and 2000 (Haack 2006) and between 1984 and 2008 (Haack et al. 2014). *Pityophagusferrugineus* is attracted to stored wood (Lindelöw et al. 1992) as well as traps baited with ethanol and alpha-pinene (Schroeder 1999) or ethanol and lineatin (Martikainen 2001). All specimens on PE were captured in low traps baited with the softwood blend (monochamol, ipsenol, fuscumol, and fuscumol acetate plus UHR alpha-pinene and UHR ethanol).

#### Family CERYLONIDAE Billberg, 1820

Two species of this small family of beetles were recorded in this study, one being new to PE. The other species was previously known from PE (Bousquet et al. 2013).

##### Subfamily CERYLONINAE Billberg, 1820

***Cerylonunicolor* (Ziegler, 1845)**

**New record. Kings Co.**, Valleyfield, 46.1356°N, 62.7198°W, 12.VI–3.VII.2018, 3.VII–13.VIII.2019, low traps (2, AFC).

**Distribution in Canada and Alaska.** ON, QC, NB, NS, **PE** (Bousquet et al. 2013).

#### Family ENDOMYCHIDAE Leach, 1815

Two species of this family were previously known from PE (Bousquet et al. 2013). In this study four species were recorded from the four woodlots, three being species new to PE.

##### Subfamily ANAMORPHINAE Strohecker, 1953

***Symbiotesduryi* Blatchley, 1910**

**New records. Kings Co.**, Valleyfield, 46.1356°N, 62.7198°W, 3.VII–2.VIII.2018, low trap (1, AFC); New Harmony, 46.3914°N, 62.2021°W, 3.VII–13.VIII.2019, low trap (1, AFC).

**Distribution in Canada and Alaska.** ON, QC, NB, **PE** (Bousquet et al. 2013; Webster et al. 2016e, 2020).

##### Subfamily ENDOMYCHINAE Leach, 1815

***Endomychusbiguttatus* Say, 1824**

**New records. Kings Co.**, Valleyfield, 46.1356°N, 62.7198°W, 4.VI–3.VII.2019, low trap (1, AFC). **Queens Co.**, Auburn, 46.2882°N, 62.9267°W, 2.VIII–13.IX.2018, canopy traps in poplar (2), low trap (3, AFC).

**Distribution in Canada and Alaska.**NT, SK, MB, ON, QC, NB, NS, **PE**, NF (Bousquet et al. 2013).

##### Subfamily LYCOPERDININAE Bromhead, 1838

***Mycetinaperpulchra* (Newman, 1838)**

**New records. Kings Co.**, Valleyfield, 46.1356°N, 62.7198°W, 3.VII–2.VIII.2018, 3.VII–13.VIII.2019, low traps (2, AFC). **Queens Co.**, Brookvale, 46.2920°N, 63.4051°W, 13.VI–3.VII.2018 (2), 3.VII–13.VIII.2019 (1), low traps (3, AFC).

**Distribution in Canada and Alaska.**MB, ON, QC, NB, NS, **PE** (Bousquet et al. 2013).

#### Family COCCINELLIDAE Latreille, 1807

Bousquet et al. (2013) reported 21 species of Coccinellidae from PE. In this study we detected nine species, three of which are newly recorded for PE.

##### Subfamily COCCINELLINAE Latreille, 1807

***Chilocorusstigma* (Say, 1835)**

**New records. Kings Co.**, Valleyfield, 46.1356°N, 62.7198°W, 12.VI–3.VII.2018 (1), 3.VII–2.VIII.2018 (1), 3.VII–13.VIII.2019 (1), canopy traps in red maple (1), in white spruce, low trap (3, AFC); New Harmony, 46.3914°N, 62.2021°W, 5.VI–3.VII.2019, canopy trap in red maple (1, AFC). **Queens Co.**, Brookvale, 46.2920°N, 63.4051°W, 13.VIII–17.IX.2019, low trap (1, AFC).

**Distribution in Canada and Alaska.**AB, SK, MB, ON, QC, NB, NS, **PE** (Bousquet et al. 2013).

***Hyperaspisbinotata* (Say, 1826)**

**New record. Kings Co.**, Valleyfield, 46.1356°N, 62.7198°W, 3.VII–2.VIII.2018, canopy trap in red maple (1, AFC).

**Distribution in Canada and Alaska.**SK, MB, ON, QC, NB, NS, **PE** (Bousquet et al. 2013).

**Scymnus (Pullus) puncticollis LeConte, 1852**

**New record. Kings Co.**, Valleyfield, 46.1356°N, 62.7198°W, 12.VI–3.VII.2018, 4.VI–3.VII.2019, canopy traps in red maple (1), in white spruce (2) (3 ♂♂ (dissected), AFC).

**Distribution in Canada and Alaska.** ON, QC, NB, **PE** (Bousquet et al. 2013; Webster et al. 2020).

#### Family CORYLOPHIDAE LeConte, 1852

One species of Corylophidae was recorded in this study and it was a new record for the province. Only one other species of this family was previously known from PE (Bousquet et al. (2013).

##### Subfamily CORYLOPHINAE LeConte, 1852

***Clypastraealunata* (LeConte, 1852)**

**New records. Kings Co.**, Valleyfield, 46.1356°N, 62.7198°W, 12.VI–3.VII.2018 (3), 3.VII–2.VIII.2018 (1), 7.V–4.VII.2019 (1), canopy traps in red maple (2), in hemlock (1), in poplar (1) low trap (5, AFC). **Queens Co.**, Auburn, 46.2882°N, 62.9267°W, 13.VI–3.VII.2018, canopy trap in red maple (1, AFC).

**Distribution in Canada and Alaska.** ON, QC, NB, NS, **PE** (Bousquet et al. 2013; Webster et al. 2016e).

#### Family LATRIDIIDAE Erichson, 1842

Bousquet et al. (2013) included 17 species of this family from PE. Hammond and Chambers (2020) added *Corticariaelongata* (Gyllenhal) in their revision of the *Corticaria*. In our study we recorded seven species, two being new records.

##### Subfamily LATRIDIINAE Erichson, 1842

***Enicmustenuicornis* LeConte, 1878**

**New records. Kings Co.**, Valleyfield, 46.1356°N, 62.7198°W, 12.VI–3.VII.2018 (1), 3.VII–2.VIII.2018 (1), 7.V–4.VI.2019 (1), canopy trap in hemlock (1), low traps (2) (3, AFC); New Harmony, 46.3914°N, 62.2021°W, 8.V–5.VI.2019, low trap (1, AFC). **Queens Co.**, Auburn, 46.2882°N, 62.9267°W, 13.VI–3.VII.2018, low trap (1, AFC); Brookvale, 46.2920°N, 63.4051°W, 13.VI–3.VII.2018 (1), 3.VII–2.VIII.2018 (1), 4.VI–3.VII.2019 (1), low traps (3, AFC).

**Distribution in Canada and Alaska.**BC, MB, QC, NB, NS, **PE** (Bousquet et al. 2013).

***Stephostethusbreviclavis* (Fall, 1899)**

**New record. Kings Co.**, Valleyfield, 46.1356°N, 62.7198°W, 12.VI–3.VII.2018, low trap (1, AFC).

**Distribution in Canada and Alaska.** ON, QC, NB, NS, **PE**, NF (Bousquet et al. 2013).

#### Family MYCETOPHAGIDAE Leach, 1815

Bousquet et al. (2013) included two species of this family from PE. In our study we recorded three species, two being new records, bringing the total number of known species for PE to four.

##### Subfamily MYCETOPHAGINAE Leach, 1815

**Mycetophagus (Mycetophagus) punctatus Say, 1826**

**New records. Kings Co.**, Valleyfield, 46.1356°N, 62.7198°W, 2.VIII–13.IX.2018, canopy trap in poplar (1, AFC); New Harmony, 46.3914°N, 62.2021°W, 5.VI–3.VII.2019, low trap (1, AFC). **Queens Co.**, Auburn, 46.2882°N, 62.9267°W, 13.VI–3.VII.2018 (2), 4.VI–3.VII.2019 (1) low traps (3, AFC); Brookvale, 46.2920°N, 63.4051°W, 13.VI–3.VII.2018 (1), 3.VII–2.VIII.2018 (3), 3.VII–13.VIII.2019 (1), low traps (4, AFC; 1, RWC).

**Distribution in Canada and Alaska.**SK, MB, ON, QC, NB, NS, **PE** (Bousquet et al. 2013).

**Mycetophagus (Parilendus) quadriguttatus P.W.J. Müller, 1821**‡

**New record. Kings Co.**, Valleyfield, 46.1356°N, 62.7198°W, 3.VII–13.VIII.2019, canopy trap in poplar (1, AFC).

**Distribution in Canada and Alaska.**BC, AB, SK, MB, ON, QC, NB, NS, **PE** (Bousquet et al. 2013).

#### Family CIIDAE Leach, 1819

Only two species of this family were previously known from PE (Bousquet et al. (2013). In this study we recorded these two species, as well as 12 new records for the island province indicating the usefulness of Lindgren funnel traps for detecting members of this family. These traps were also very effective for detecting species of this family in NB (Lopes-Andrade et al. 2016).

##### Subfamily CIINAE Leach, 1819

***Ceracisthoracicornis* (Ziegler, 1845)**

**New record. Queens Co.**, Brookvale, 46.2920°N, 63.4051°W, 3.VII–13.VIII.2019, low trap (1, AFC).

**Distribution in Canada and Alaska.**MB, ON, QC, NB, NS, **PE** (Bousquet et al. 2013; Lopes-Andrade et al. 2016).

***Cisangustus* Hatch, 1962**

**New record. Kings Co.**, Valleyfield, 46.1356°N, 62.7198°W, 3.VII–2.VIII.2018, low trap (black) (1, RWC).

**Distribution in Canada and Alaska.**BC, NB, **PE** (Bousquet et al. 2013; Lopes-Andrade et al. 2016).

**Note.**Lawrence (1971) noted that this species was restricted to the coniferous forests at higher elevations of the Pacific coast of BC but mentioned that additional fieldwork might reveal a broader distribution. Lopes-Andrade et al. (2016) reported it from NB and here we report it from PE based on the record above. This species will likely be found in intervening areas of Canada.

***Ciscreberrimus* Mellié, 1849**

**New record. Queens Co.**, Auburn, 46.2882°N, 62.9267°W, 3.VII–2.VIII.2018, low trap (1, RWC).

**Distribution in Canada and Alaska.** ON, QC, NB, NS, **PE** (Bousquet et al. 2013; Lopes-Andrade et al. 2016).

***Cisfuscipes* Mellié, 1849**

**New records. Kings Co.**, Valleyfield, 46.1356°N, 62.7198°W, 4.VI–3.VII.2019, 13.VIII–17.IX.2019, low traps (2, AFC); New Harmony, 46.3914°N, 62.2021°W, 5.VI–3.VII.2019, canopy trap in white pine (1), low trap (1) (2, AFC). **Queens Co.**, Auburn, 46.2882°N, 62.9267°W, 13.VI–3.VII.2018 (4), 2.VIII–13.IX.2018 (1), 3.VII–14.VIII.2019 (1), canopy trap in poplar (1), low traps (5) (6, AFC); Brookvale, 46.2920°N, 63.4051°W, 13.VI–3.VII.2018, low trap (1, AFC).

**Distribution in Canada and Alaska.**NT, BC, AB, SK, MB, ON, QC, NB, NS, **PE**, NF (Bousquet et al. 2013; Lopes-Andrade et al. 2016).

***Cishorridulus* Casey, 1898**

**New records. Kings Co.**, Valleyfield, 46.1356°N, 62.7198°W, 12.VI–3.VII.2018, low trap (1, AFC); New Harmony, 46.3914°N, 62.2021°W, 5.VI–3.VII.2019, 3.VII–13.VIII.2019, low traps (2, AFC). **Queens Co.**, Brookvale, 46.2920°N, 63.4051°W, 13.VII–13.VIII.2019, low trap (1, AFC).

**Distribution in Canada and Alaska.**NT, BC, MB, ON, QC, NB, NS, **PE** (Bousquet et al. 2013; Lopes-Andrade et al. 2016).

*Cisstriatulus* Mellié, 1849*

**New records. Kings Co.**, Valleyfield, 46.1356°N, 62.7198°W, 4.VI–3.VII.2019, low trap (1, AFC). **Queens Co.**, Auburn, 46.2882°N, 62.9267°W, 2.VIII–13.IX.2018, low trap (1, AFC).

**Distribution in Canada and Alaska.**NT, BC, AB, ON, QC, NB, NS, **PE** (Bousquet et al. 2013; Lopes-Andrade et al. 2016).

**Note.**Lopes-Andrade et al. (2016) synonymized *Cisstriolata* Casey, 1898 with *C.striatulus*.

*Cissubmicans* Abeille de Perrin, 1874*

**New records. Kings Co.**, Valleyfield, 46.1356°N, 62.7198°W, 7.V–4.VI.2019, 4.VI–3.VII.2019, low traps (2, AFC).

**Distribution in Canada and Alaska.**NT, AB, SK, MB, ON, QC, NB, NS, **PE** (Bousquet et al. 2013; Lopes-Andrade et al. 2016).

**Note.** Lopes-Andrade (2016) synonymized *Cispistorius* Casey, 1898 with *C.submicans*.

*Dolichocislaricinus* (Mellié, 1849)*

**New record. Kings Co.**, Valleyfield, 46.1356°N, 62.7198°W, 3.VII–13.VIII.2019, low trap (1, RWC).

**Distribution in Canada and Alaska.**BC, QC, NB, **PE** (Bousquet et al. 2013; Lopes-Andrade et al. 2016).

**Note.**Lopes-Andrade et al. (2016) synonymized *Dolichocisindistinctus* Hatch, 1962 with *D.laricinus*.

***Hadreuleelongatula* (Gyllenhal, 1827)**†

**New record. Queens Co.**, Auburn, 46.2882°N, 62.9267°W, 3.VII–2.VIII.2018, canopy trap in poplar (1, AFC).

**Distribution in Canada and Alaska.**NB, **PE** (Bousquet et al. 2013; Lopes-Andrade et al. 2016).

***Malacocisbrevicollis* (Casey, 1898)**

**New records. Kings Co.**, Valleyfield, 46.1356°N, 62.7198°W, 3.VII–2.VIII.2018, 3.VII–13.VIII.2019, low traps (2, AFC). **Queens Co.**, Auburn, 46.2882°N, 62.9267°W, 13.VI–3.VII.2018, 3.VII–14.VIII.2019, low traps (2, AFC); Brookvale, 46.2920°N, 63.4051°W, 13.VI–3.VII.2018 (1), 3.VII–2.VIII.2018 (1), 3.VII–13.VIII.2019 (1), low traps (3, AFC).

**Distribution in Canada and Alaska.**MB, ON, QC, NB, NS, **PE**, NF (Bousquet et al. 2013; Lopes-Andrade et al. 2016).

***Orthocispunctatus* (Mellié, 1849)**

**New records. Kings Co.**, Valleyfield, 46.1356°N, 62.7198°W, 7.V–4.VI.2019, canopy trap in in poplar (1, AFC); New Harmony, 46.3914°N, 62.2021°W, 3.VII–13.VIII.2019, canopy trap in sugar maple (1, AFC). **Queens Co.**, Auburn, 46.2882°N, 62.9267°W, 7.V–4.VI.2019, low trap (1, AFC); Brookvale, 46.2920°N, 63.4051°W, 13.VI–3.VII.2018 (1), 3.VII–2.VIII.2018 (1), 4.VI–3.VII.2019 (1), 3.VII–13.VIII.2019 (1), canopy traps in hemlock (2), low traps (2) (4, AFC).

**Distribution in Canada and Alaska.**AK, NT, BC, AB, MB, ON, QC, NB, NS, **PE**, NF (Bousquet et al. 2013; Lopes-Andrade et al. 2016).

***Plesiociscribrum* Casey, 1898**

**New record. Kings Co.**, New Harmony, 46.3914°N, 62.2021°W, 3.VII–13.VIII.2019, canopy trap in white pine (3), low trap (1) (4, RWC).

**Distribution in Canada and Alaska.**BC, AB, MB, QC, NB, **PE** (Bousquet et al. 2013; Lopes-Andrade et al. 2016).

#### Family TETRATOMIDAE Billberg, 1820

Five species of this family were recorded from the four woodlots and four of these represented new records for PE. Only two species of Tetratomidae were previously known from PE (Bousquet et al. 2013).

##### Subfamily TETRATOMINAE Billberg, 1820

**Tetratoma (Abstrulia) canadensis Nikitsky & Chantal, 2004**

**New record. Queens Co.**, Brookvale, 46.2920°N, 63.4051°W, 13.VI–3.VII.2018, low trap (1, RWC).

**Distribution in Canada and Alaska.**QC, NB, **PE** (Bousquet et al. 2013; Webster et al. 2016e).

**Tetratoma (Abstrulia) tesselata (Melsheimer, 1844)**

**New records. Kings Co.**, Valleyfield, 46.1356°N, 62.7198°W, 12.VI–3.VII.2018 (1), 3.VII–2.VIII.2018 (1), 13.VIII–17.IX.2019 (1), canopy traps in hemlock (1), red maple (1), low trap (1) (2, AFC: 1, RWC). **Queens Co.**, Auburn, 46.2882°N, 62.9267°W, 3.VII–2.VIII.2018 (1), 2.VIII–13.IX.2018 (1), canopy trap in poplar (1), low trap (black, 1) (2, AFC); Brookvale, 46.2920°N, 63.4051°W, 13.VIII–17.IX.2019, low trap (1, AFC).

**Distribution in Canada and Alaska.** ON, QC, NB, NS, **PE** (Bousquet et al. 2013).

##### Subfamily PENTHINAE Lacordaire, 1859

***Pentheobliquata* (Fabricius, 1801)**

**New records. Kings Co.**, Valleyfield, 46.1356°N, 62.7198°W, 3.VII–2.VIII.2018, low trap (1, AFC). **Queens Co.**, Auburn, 46.2882°N, 62.9267°W, 3.VII–2.VIII.2018, low trap (1, AFC); Brookvale, 46.2920°N, 63.4051°W, 3.VII–2.VIII.2018, 3.VII–13.VIII.2019, low traps (2, AFC).

**Distribution in Canada and Alaska.**MB, ON, QC, NB, NS, **PE** (Bousquet et al. 2013).

##### Subfamily EUSTROPHINAE Gistel, 1848

***Eustrophustomentosus* Say, 1826**

**New record. Kings Co.**, Valleyfield, 46.1356°N, 62.7198°W, 4.VI–3.VII.2019, 3.VII–13.VIII.2019, low traps (2, AFC).

**Distribution in Canada and Alaska.**BC, MB, ON, QC, NB, NS, **PE** (Bousquet et al. 2013).

#### Family MELANDRYIDAE Leach, 1815

Bousquet et al. (2013) listed five species of this family for PE. In this study we recorded 17 species at the four woodlots including the five previously known species. The other 12 species are newly recorded for PE and we were able to detect all the Melandryidae known from PE using Lindgren funnel traps.

##### Subfamily MELANDRYINAE Leach, 1815

***Hypulussimulator* Newman, 1838**

**New records. Kings Co.**, Valleyfield, 46.1356°N, 62.7198°W, 4.VI–3.VII.2019 (1), 3.VII–13.VIII.2019 (1), low traps (2, AFC). **Queens Co.**, Brookvale, 46.2920°N, 63.4051°W, 4.VI–3.VII.2019, low trap (1, AFC).

**Distribution in Canada and Alaska.** ON, QC, NB, NS, **PE**, NF (Bousquet et al. 2013).

***Emmesaconnectens* Newman, 1838**

**New records. Kings Co.**, Valleyfield, 46.1356°N, 62.7198°W, 12.VI–3.VII.2018 (2), 3.VII–2.VIII.2018 (1), 4.VI–3.VII.2019 (1), low traps (4, AFC); New Harmony, 46.3914°N, 62.2021°W, 3.VII–13.VIII.2019, canopy trap in red maple (1, AFC). **Queens Co.**, Auburn, 46.2882°N, 62.9267°W, 13.VI–3.VII.2018 (1), 3.VII–2.VIII.2018 (1), 4.VI–3.VII.2019 (1), low traps (3, AFC); Brookvale, 46.2920°N, 63.4051°W, 3.VII–2.VIII.2018, 3.VII–13.VIII.2019, low traps (2, AFC).

**Distribution in Canada and Alaska.**MB, ON, QC, NB, NS, **PE**, LB, NF (Bousquet et al. 2013).

***Orchesiacastanea* (Melsheimer, 1846)**

**New records. Kings Co.**, Valleyfield, 46.1356°N, 62.7198°W, 3.VII–13.VIII.2019, low trap (1, AFC). **Queens Co.**, Auburn, 46.2882°N, 62.9267°W, 2.VIII–13.IX.2018 (2), 14.VIII–17.IX.2019 (1), canopy traps in poplar (2), low trap (1) (3, AFC); Brookvale, 46.2920°N, 63.4051°W, 2.VIII–13.IX.2018, low trap (1, AFC).

**Distribution in Canada and Alaska.**BC, SK, MB, ON, QC, NB, NS, **PE**, NF (Bousquet et al. 2013).

***Orchesiacultriformis* Laliberté, 1967**

**New records. Kings Co.**, Valleyfield, 46.1356°N, 62.7198°W, 2.VIII–13.IX.2018 (3), 13.VIII–17.IX.2019 (1), low traps (4, AFC); New Harmony, 46.3914°N, 62.2021°W, 13.VIII–17.IX.2019, canopy trap in sugar maple (1, AFC). **Queens Co.**, Auburn, 46.2882°N, 62.9267°W, 3.VII–14.VIII.2019, canopy trap in poplar (1, AFC); Brookvale, 46.2920°N, 63.4051°W, 3.VII–2.VIII.2018 (4), 2.VIII–13.IX.2018 (2), 3.VII–13.VIII.2019 (1), low traps (7, AFC).

**Distribution in Canada and Alaska.**NT, SK, MB, QC, NB, NS, **PE** (Bousquet et al. 2013).

*Dolotarsuslividus* (C.R. Sahlberg, 1833)*

**New records. Kings Co.**, Valleyfield, 46.1356°N, 62.7198°W, 7.V–4.VI.2019 (1), 4.VI–3.VII.2019 (2), canopy trap in poplar (1) low traps (2) (3, AFC). **Queens Co.**, Brookvale, 46.2920°N, 63.4051°W, 13.VI–3.VII.2018 (1), 3.VII–2.VIII.2018 (1), low traps (2, AFC).

**Distribution in Canada and Alaska.**YT, BC, AB, QC, NB, NS, **PE**, LB, NF (Bousquet et al. 2013).

***Enchodessericea* (Haldeman, 1848)**

**New records. Kings Co.**, Valleyfield, 46.1356°N, 62.7198°W, 3.VII–2.VIII.2018 (2), 3.VII–13.VIII.2019 (1), canopy trap in poplar (1) low traps (2) (3, AFC); New Harmony, 46.3914°N, 62.2021°W, 13.VIII–17.IX.2019, canopy trap in sugar maple (1, AFC). **Queens Co.**, Auburn, 46.2882°N, 62.9267°W, 3.VII–14.VIII.2019, low trap (1, AFC); Brookvale, 46.2920°N, 63.4051°W, 3.VII–13.VIII.2019, low trap (1, AFC).

**Distribution in Canada and Alaska.**BC, AB, SK, MB, ON, QC, NB, NS, **PE** (Bousquet et al. 2013).

***Scotochroaatra* LeConte, 1874**

**New records. Kings Co.**, Valleyfield, 46.1356°N, 62.7198°W, 12.VI–3.VII.2018 (2) 3.VII–2.VIII.2018 (1), 2.VIII–13.IX.2018 (1), canopy traps in hemlock (2, AFC; 2, RWC).

**Distribution in Canada and Alaska.**QC, NB, NS, **PE**, NF (Bousquet et al. 2013).

***Scotochroabuprestoides* (Kirby, 1837)**

**New records. Queens Co.**, Auburn, 46.2882°N, 62.9267°W, 3.VII–2.VIII.2018 (2), 3.VII–14.VIII.2019 (1), low traps (3, AFC); Brookvale, 46.2920°N, 63.4051°W, 3.VII–2.VIII.2018 (2), 3.VII–13.VIII.2019 (2), canopy trap in white pine (1), low traps (3) (4, AFC).

**Distribution in Canada and Alaska.**SK, MB, ON, QC, NB, NS, **PE**, NF (Bousquet et al. 2013).

***Scotochroidesantennatus* Mank, 1839**

**New records. Kings Co.**, Valleyfield, 46.1356°N, 62.7198°W, 3.VII–2.VIII.2018, low trap (1, AFC). **Queens Co.**, Auburn, 46.2882°N, 62.9267°W, 3.VII–14.VIII.2019, canopy trap in poplar (1, AFC).

**Distribution in Canada and Alaska.**QC, NB, NS, **PE** (Bousquet et al. 2013).

***Serropalpuscoxalis* Mank, 1839**

**New records. Kings Co.**, Valleyfield, 46.1356°N, 62.7198°W, 3.VII–2.VIII.2018, low traps (1, AFC). **Queens Co.**, Auburn, 46.2882°N, 62.9267°W, 3.VII–2.VIII.2018, low trap (1, AFC); Brookvale, 46.2920°N, 63.4051°W, 3.VII–2.VIII.2018 (3), 3.VII–13.VIII.2019 (1), low traps (4, AFC).

**Distribution in Canada and Alaska.**AK, BC, MB, ON, QC, NB, NS, **PE**, NF (Bousquet et al. 2013).

***Serropalpussubstriatus* Haldeman, 1848**

**New records. Kings Co.**, Valleyfield, 46.1356°N, 62.7198°W, 2.VIII–13.IX.2018 (5), 3.VII–13.VIII.2019 (2), canopy trap in poplar (1), low traps (6) (7, AFC). **Queens Co.**, Auburn, 46.2882°N, 62.9267°W, 3.VII–2.VIII.2018 (2), 3.VII–14.VIII.2019 (1), canopy trap in poplar (1), low traps (2) (3, AFC); Brookvale, 46.2920°N, 63.4051°W, 3.VII–2.VIII.2018 (8), 3.VII–13.VIII.2019 (2), low traps (10, AFC).

**Distribution in Canada and Alaska.**AK, NT, BC, AB, SK, MB, ON, QC, NB, NS, **PE**, LB, NF (Bousquet et al. 2013).

*Xylitalaevigata* (Hellenius, 1786)*

**New records. Kings Co.**, Valleyfield, 46.1356°N, 62.7198°W, 4.VI–3.VII.2019, 3.VII–13.VIII.2019, low traps (2, AFC); New Harmony, 46.3914°N, 62.2021°W, 5.VI–3.VII.2019, low trap (1, AFC). **Queens Co.**, Auburn, 46.2882°N, 62.9267°W, 13.VI–3.VII.2018 (2), 7.V–4.VII.2019 (2), low traps (4, AFC); Brookvale, 46.2920°N, 63.4051°W, 13.VI–3.VII.2018 (1), 7.V–4.VI.2019 (1), 4.VI–3.VII.2019 (1), canopy trap in white pine (1), low trap (1) (3, AFC).

**Distribution in Canada and Alaska.**AK, BC, AB, SK, MB, ON, QC, NB, NS, **PE**, NF (Bousquet et al. 2013).

#### Family MORDELLIDAE Latreille, 1802

Fourteen species of Mordellidae were listed by Bousquet et al. (2013) from PE. In this study we recorded 11 species; five are new records for the province.

##### Subfamily MORDELLINAE Latreille, 1802

***Tomoxialineela* LeConte, 1862**

**New records. Kings Co.**, Valleyfield, 46.1356°N, 62.7198°W, 3.VII–2.VIII.2018 (2), 2.VIII–13.IX.2018 (1), 3.VII–13.VIII.2019 (1), low traps (4, AFC); New Harmony, 46.3914°N, 62.2021°W, 3.VII–13.VIII.2019, low trap (1, AFC). **Queens Co.**, Auburn, 46.2882°N, 62.9267°W, 3.VII–2.VIII.2018 (4), 2.VIII–13.IX.2018 (1), 3.VII–14.VIII.2019 (1), canopy traps in poplar (4), low traps (2) (6, AFC); Brookvale, 46.2920°N, 63.4051°W, 3.VII–2.VIII.2018 (2), 3.VII–13.VIII.2019 (1), low traps (3, AFC).

**Distribution in Canada and Alaska.**SK, ON, QC, NB, NS, **PE** (Bousquet et al. 2013).

***Mordellistenaandreae* LeConte, 1862**

**New records. Queens Co.**, Brookvale, 46.2920°N, 63.4051°W, 3.VII–2.VIII.2018, low trap (1, RWC); Auburn, 46.2882°N, 62.9267°W, 3.VII–2.VIII.2018, canopy trap in poplar (1, AFC).

**Distribution in Canada and Alaska.** ON, QC, NB, **PE** (Bousquet et al. 2013; Webster et al. 2020).

**Note.**Bousquet et al. (2013) placed this species in the genus *Mordellina*, but Lisberg (2003) recommended keeping it in the genus *Mordellistena*, noting that it was not well placed in either genus.

***Mordellistenafrosti* Liljeblad, 1918**

**New records. Kings Co.**, Valleyfield, 46.1356°N, 62.7198°W, 3.VII–2.VIII.2018, low trap (1, AFC). **Queens Co.**, Auburn, 46.2882°N, 62.9267°W, 13.VI–3.VII.2018, canopy trap in poplar (1, RWC).

**Distribution in Canada and Alaska.**AB, MB, QC, NB, NS, **PE** (Bousquet et al. 2013).

***Mordellistenatosta* LeConte, 1862**

**New records. Kings Co.**, Valleyfield, 46.1356°N, 62.7198°W, 3.VII–2.VIII.2018, canopy trap in red maple (1, AFC). **Queens Co.**, Auburn, 46.2882°N, 62.9267°W, 3.VII–13.VIII.2019, canopy trap in poplar (1, AFC).

**Distribution in Canada and Alaska.**SK, MB, ON, QC, NB, NS, **PE** (Bousquet et al. 2013).

***Mordellochroascapularis* (Say, 1824)**

**New record. Queens Co.**, Brookvale, 46.2920°N, 63.4051°W, 3.VII–2.VIII.2018, low trap (1, AFC).

**Distribution in Canada and Alaska.**BC, AB, SK, MB, ON, QC, NB, NS, **PE** (Bousquet et al. 2013).

#### Family RIPIPHORIDAE Gemminger, 1870

This is the first record of this family for PE. Webster et al. (2022) recently reported it from NS.

##### Subfamily PELECOTOMINAE Seidlitz, 1875

***Pelecotomaflavipes* Melsheimer, 1846**

**New records. Kings Co.**, Valleyfield, 46.1356°N, 62.7198°W, 3.VII–2.VIII.2018, canopy trap in red maple (1, AFC). **Queens Co.**, Auburn, 46.2882°N, 62.9267°W, 13.VI–3.VII.2018, canopy trap in poplar (1), low trap (1) (2, AFC).

**Distribution in Canada and Alaska.**MB, ON, QC, NB, NS, **PE** (Bousquet et al. 2013; Webster et al. 2022).

#### Family ZOPHERIDAE Solier, 1834

*Phellopsisobcordata* (Kirby) was the only species of this family previously known from PE (Bousquet et al. 2013). Here we report another two species for the province.

##### Subfamily COLYDIINAE Billberg, 1820

***Lasconotusborealis* Horn, 1878**

**New record. Queens Co.**, Brookvale, 46.2920°N, 63.4051°W, 3.VII–2.VIII.2018, low traps (2, AFC).

**Distribution in Canada and Alaska.** (Bousquet et al. 2013).

***Synchitafuliginosa* Melsheimer, 1844**

**New records. Kings Co.**, Valleyfield, 46.1356°N, 62.7198°W, 3.VII–2.VIII.2018, 3.VII–13.VIII.2019, canopy traps in red maple (1), in white spruce (1) (2, AFC). **Queens Co.**, Auburn, 46.2882°N, 62.9267°W, 13.VI–3.VII.2018 (3), 3.VII–14.VIII.2019 (1), canopy trap in poplar (1), low traps (3) (4, AFC).

**Distribution in Canada and Alaska.**SK, MB, ON, QC, NB, NS, **PE** (Bousquet et al. 2013).

#### Family TENEBRIONIDAE Latreille, 1802

Bousquet et al. (2013) listed 17 species of Tenebrionidae from PE. In this study 15 species were recorded from Lindgren funnel traps, of which eight are new records.

##### Subfamily LAGRIINAE Latreille, 1802

***Paratenetusexutus* Bousquet & Bouchard, 2014**

**New records. Kings Co.**, Valleyfield, 46.1356°N, 62.7198°W, 12.VI–3.VII.2018 (4).VII–2.VIII.2018 (1), 7.V–4.VI.2019 (1), canopy traps in poplar (4), low traps (2) (6, AFC). **Queens Co.**, Auburn, 46.2882°N, 62.9267°W, 13.VI–3.VII.2018 (3), 3.VII–2.VIII.2018 (1), 2.VIII–13.IX.2018 (1), 7.V–4.VI.2019 (2), canopy traps in poplar (4), low traps (3) (7, AFC); Brookvale, 46.2920°N, 63.4051°W, 13.VI–3.VII.2018 (2), 3.VII–2.VIII.2018 (1), 7.V–4.VI,2019 (1), 4.VI–3.VII.2019 (1), canopy trap in white pine (1), low traps (3) (4, AFC).

**Distribution in Canada and Alaska.**AB, SK, MB, ON, QC, NB, NS, **PE** (Bousquet and Bouchard 2014).

##### Subfamily TENEBRIONINAE Latreille, 1802

***Neatustenebrioides* (Palisot de Beauvois, 1811**)

**New record. Queens Co.**, Auburn, 46.2882°N, 62.9267°W, 3.VII–2.VIII.2018, canopy trap in red maple (1, RWC).

**Distribution in Canada and Alaska.**AB, SK, MB, ON, QC, NB, **PE** (Bousquet et al. 2013).

##### Subfamily ALLECULINAE Laporte, 1840

***Androchiruserythropus* (Kirby, 1837)**

**New records. Kings Co.**, Valleyfield, 46.1356°N, 62.7198°W, 3.VII–2.VIII.2018 (2), 3.VII–13.VIII.2019 (1), canopy trap in poplar (1), low traps (2) (3, AFC); New Harmony, 46.3914°N, 62.2021°W, 3.VII–13.VIII.2019, canopy trap in red maple (1, AFC). **Queens Co.**, Auburn, 46.2882°N, 62.9267°W, 3.VII–2.VIII.2018 (1), 3.VII–14.VIII.2019 (2), canopy trap in poplar (1), low traps (2) (3, AFC); Brookvale, 46.2920°N, 63.4051°W, 3.VII–2.VIII.2018 (2), 3.VII–13.VIII.2019 (1), low traps (3, AFC).

**Distribution in Canada and Alaska.**MB, ON, QC, NB, NS, **PE** (Bousquet et al. 2013).

***Mycetocharabicolor* (Couper, 1865)**

**New records. Kings Co.**, Valleyfield, 46.1356°N, 62.7198°W, 3.VII–2.VIII.2018 (3), 3.VII–13.VIII.2019 (3), canopy traps in poplar (2), in red maple (1), in white spruce (1), low traps (2) (6, AFC); New Harmony, 46.3914°N, 62.2021°W, 3.VII–13.VIII.2019, canopy trap in sugar maple (1, AFC). **Queens Co.**, Auburn, 46.2882°N, 62.9267°W, 3.VII–2.VIII.2018 (2), 3.VII–14.VIII.2019 (2), canopy trap in poplar (1), low traps (3) (4, AFC); Brookvale, 46.2920°N, 63.4051°W, 3.VII–2.VIII.2018 (3), 3.VII–13.VIII.2019 (1), low traps (4, AFC).

**Distribution in Canada and Alaska.** ON, QC, NB, NS, **PE** (Bousquet et al. 2013).

***Mycetocharabinotata* (Say, 1824)**

**New records. Kings Co.**, Valleyfield, 46.1356°N, 62.7198°W, 3.VII–2.VIII.2018 (1), 2.VIII–13.IX.2018 (1), 3.VII–13.VIII.2019 (2), canopy trap in white spruce (1), low traps (3) (4, AFC); New Harmony, 46.3914°N, 62.2021°W, 3.VII–13.VIII.2019, canopy trap in red maple (1), low trap (1) (2, AFC). **Queens Co.**, Auburn, 46.2882°N, 62.9267°W, 3.VII–2.VIII.2018, canopy trap in red maple (1, AFC).

**Distribution in Canada and Alaska.**MB, ON, QC, NB, NS, **PE** (Bousquet et al. 2013).

***Mycetocharafoveata* (LeConte, 1866)**

**New records. Kings Co.**, Valleyfield, 46.1356°N, 62.7198°W, 2.VIII–13.IX.2018, 3.VII–13.VIII.2019, canopy traps in red maple, in white spruce (2, AFC); New Harmony, 46.3914°N, 62.2021°W, 3.VII–13.VIII.2019, canopy trap in sugar maple, low trap (2, AFC).

**Distribution in Canada and Alaska.** ON, QC, NB, NS, **PE** (Bousquet et al. 2013; Webster et al. 2022).

***Mycetocharafraterna* (Say, 1824)**

**New records. Kings Co.**, Valleyfield, 46.1356°N, 62.7198°W, 3.VII–2.VIII.2018 (2), 3.VII–13.VIII.2019 (1), canopy trap in poplar (1), low traps (2) (3, AFC); New Harmony, 46.3914°N, 62.2021°W, 3.VII–13.VIII.2019, canopy trap in red maple (1, AFC). **Queens Co.**, Auburn, 46.2882°N, 62.9267°W, 3.VII–2.VIII.2018, canopy trap in poplar (1), low trap (1) (2, AFC); Brookvale, 46.2920°N, 63.4051°W, 3.VII–13.VIII.2019, low trap (1, AFC).

**Distribution in Canada and Alaska.**BC, AB, SK, MB, ON, QC, NB, NS, **PE** (Bousquet et al. 2013).

##### Subfamily DIAPERINAE Latreille, 1802

***Corticeuspraetermissus* (Fall, 1826)**

**New records. Kings Co.**, Valleyfield, 46.1356°N, 62.7198°W, 12.VI–3.VII.2018 (3), 4.VI–3.VII.2019 (1), low traps (4, AFC); New Harmony, 46.3914°N, 62.2021°W, 5.VI–3.VII.2019, canopy trap in white pine (1, AFC). **Queens Co.**, Auburn, 46.2882°N, 62.9267°W, 13.VI–3.VII.2018 (1), 3.VII–2.VIII.2018 (1), 4.VI–3.VII.2019 (1), low traps (3, AFC); Brookvale, 46.2920°N, 63.4051°W, 13.VI–3.VII.2018 (1) 3.VII–2.VIII.2018 (1), 3.VII–13.VIII.2019 (1), low traps (3, AFC).

**Distribution in Canada and Alaska.**AK, YT, NT, BC, AB, SK, MB, ON, QC, NB, NS, **PE**, LB, NF (Bousquet et al. 2013).

#### Family SYNCHROIDAE Kirby, 1837

*Synchroapunctata* Newman is the only member of this family known from PE and the Maritime Provinces as a whole (Bousquet et al. 2013). This species was recorded from the Auburn and Brookvale Woodlots.

#### Family STENOTRACHELIDAE C. G. Thomson, 1859

Two species were recorded from PE by Bousquet et al. (2013). One of these, *Cephaloonlepturoides* Newman, was recorded from three of the four sites.

#### Family OEDEMERIDAE Latreille, 1810

Only the adventive *Nacerdesmelanura* (Linnaeus) was previously known from PE (Bousquet et al. 2013). Here we report three additional members of this family for PE.

##### Subfamily CALOPODINAE Costa, 1852

***Calopusangustus* LeConte, 1851**

**New records. Kings. Co.**, Valleyfield, 46.1356°N, 62.7198°W, 7.V–4.VI.2019, canopy trap in poplar (1), low trap (1) (2, AFC); New Harmony, 46.3914°N, 62.2021°W, 8.V–5.VI.2019, 5.VI–3.VII.2019, low traps (2, AFC). **Queens Co.**, Brookvale, 46.2920°N, 63.4051°W, 7.V–4.VI.2019, 4.VI–3.VII.2019, low traps (2, AFC).

**Distribution in Canada and Alaska.**BC, AB, ON, QC, NB, NS, **PE** (Bousquet et al. 2013).

##### Subfamily OEDEMERINAE Latreille, 1810

***Asclerapuncticollis* (Say, 1824)**

**New record. Kings. Co.**, Valleyfield, 46.1356°N, 62.7198°W, 4.VI–3.VII.2019, 3.VII–13.VIII.2019, canopy traps in poplar (1), in white spruce (1) (2, AFC).

**Distribution in Canada and Alaska.**SK, MB, On, QC, NB, NS, **PE** (Bousquet et al. 2013).

***Ascleraruficollis* (Say, 1824)**

**New record. Queens Co.**, Brookvale, 46.2920°N, 63.4051°W, 13.VI–3.VII.2018, low trap (1 AFC).

**Distribution in Canada and Alaska.** ON, QC, NB, NS, **PE** (Bousquet et al. 2013).

#### Family MELOIDAE Gyllenhal, 1810

Webster et al. (2022) reported *Meloeimpressus* from PE. This is the first record of this family for the province.

#### Family PYTHIDAE Solier, 1834

One of the two known species of this small family reported by Bousquet et al. (2013) was recorded in this study.

#### Family PYROCHROIDAE Latreille, 1806

All three species listed for PE by Bousquet et al. (2013) were recorded from Lindgren funnel traps in this study.

#### Family SALPINGIDAE Leach, 1815

This is the first record of this family for PE.

##### Subfamily SALPINGINAE Leach, 1815

***Rhinosimusviridiaeneus* Randall, 1838**

**New records. Kings Co.**, Valleyfield, 46.1356°N, 62.7198°W, 12.VI–3.VII.2018, 7.V–4.VI.2019, low traps (2, AFC); New Harmony, 46.3914°N, 62.2021°W, 8.V–5.VI.2019, low trap (1, AFC). **Queens Co.**, Auburn, 46.2882°N, 62.9267°W, 13.VI–3.VII.2018, 7.V–4.VI.2019, low traps (2, AFC); Brookvale, 46.2920°N, 63.4051°W, 13.VI–3.VII.2018 (2), 7.V–4.VI.2019 (1), canopy trap in white pine (1), low traps (2) (3, AFC).

**Distribution in Canada and Alaska.**AK, BC, ON, QC, NB, NS, **PE**, NF (Bousquet et al. 2013).

#### Family ANTHICIDAE Latreille, 1819

Bousquet et al. (2013) listed seven species of Anthicidae for PE. We did not record any members of this family from Lindgren funnel traps in this study.

#### Family ADERIDAE Csiki, 1909

The only member of this family known from PE reported by Bousquet et al. (2013) is *Vanonuswickhami* Casey. It was recorded from the Auburn Woodlot.

#### Family SCRAPTIIDAE Gistel, 1848

All three of the species reported by Bousquet et al. (2013) were recorded in this study from Lindgren funnel traps.

#### Family CERAMBYCIDAE Latreille, 1802

Bousquet et al. (2013) listed 43 species of Cerambycidae from PE. In this study, we recorded 50 species from Lindgren funnel traps from the four woodlots. Among these were 24 species new to PE, demonstrating the effectiveness of these traps for detecting species of this family.

##### Subfamily PRIONINAE Latreille, 1802

***Tragosomaharrisii* LeConte, 1851**

**New records. Kings Co.**, Valleyfield, 46.1356°N, 62.7198°W, 3.VII–13.VIII.2019, canopy trap in poplar (1, AFC). **Queens Co.**, Brookvale, 46.2920°N, 63.4051°W, 3.VII–13.VIII.2019 (1), 13.VIII–17.IX.2019 (2), low traps (2, AFC).

**Distribution in Canada and Alaska.**AK, NT, BC, AB, SK, MB, ON, QC, NB, NS, **PE**, NF (Bousquet et al. 2013).

##### Subfamily LEPTURINAE Latreille, 1802

***Judoliamontivagansmontivagans* (Couper, 1864)**

**New record. Queens Co.**, Auburn, 46.2882°N, 62.9267°W, 3.VII–2.VIII.2018, canopy trap in poplar (1) low trap (black, 1) (2 AFC).

**Distribution in Canada and Alaska.**AK, YT, NT, BC, SK, MB, ON, QC, NB, NS, **PE**, LB (Bousquet et al. 2013).

***Anthophylaxcyaneus* (Haldeman, 1847)**

**New record. Kings Co.**, Valleyfield, 46.1356°N, 62.7198°W, 12.VI–3.VII.2018, canopy trap in poplar (1, AFC).

**Distribution in Canada and Alaska.** ON, QC, NB, NS, **PE** (Bousquet et al. 2013).

**Pidonia (Pidonia) vibex (Newman, 1841)**

**New records. Queens Co.**, Auburn, 46.2882°N, 62.9267°W, 13.VI–3.VII.2018 (2), 3.VII–14.VIII.2019 (1), canopy traps in poplar (2), low trap (1) (3, AFC); Brookvale, 46.2920°N, 63.4051°W, 3.VII–2.VIII.2018 (1), 4.VI–3.VII.2019 (1), 3.VII–13.VIII.2019 (2), low traps (4, AFC).

**Distribution in Canada and Alaska.** ON, QC, NB, NS, **PE** (Bousquet et al. 2013).

##### Subfamily SPONDYLIDINAE Audinet-Serville, 1832

***Tetropiumschwarzianum* Casey, 1891**

**New record. Queens Co.**, Brookvale, 46.2920°N, 63.4051°W, 13.VI–3.VII.2018, low traps (2, AFC).

**Distribution in Canada and Alaska.** ON, QC, NB, NS, **PE**, NF (Bousquet et al. 2013).

##### Subfamily CERAMBYCINAE Latreille, 1802

***Phymatodesmaculicollis* LeConte, 1878**

**New records. Kings Co.**, Valleyfield, 46.1356°N, 62.7198°W, 12.VI–3.VII.2018 (3), 4.VI–3.VII.2019 (1), canopy trap in white spruce (1), low traps (3) (4, AFC). **Queens Co.**, Auburn, 46.2882°N, 62.9267°W, 13.VI–3.VII.2018 (3), 4.VI–3.VII.2019 (1), canopy traps in poplar (4, AFC); Brookvale, 46.2920°N, 63.4051°W, 13.VI–3.VII.2018 (5), 4.VI–3.VII.2019 (1), low traps (6, AFC).

**Distribution in Canada and Alaska.**AK, YT, BC, AB, QC, NB, **PE** (Bousquet et al. 2013).

***Phymatode* s *heutheri* Wappes & Santos-Silva, 2019**

**New record. Queens Co.**, Brookvale, 46.2920°N, 63.4051°W, 3.VII–2.VIII.2018, 3.VII–13.VIII.2019, canopy trap in hemlock (1), low trap (1) (1, AFC; 1 RWC).

**Distribution in Canada and Alaska.**AB, ON, QC, NB, NS, **PE** (Webster et al. 2012c, Wappes and Santos-Silva 2019).

**Note.** This is the same species as *Phymatodes* sp. A of Bousquet et al. (2017) (Wappes and Santos-Silva 2019), and also as *Phymatodes* species (CNC sp. nov. #1) reported from NB by Webster et al. (2012c) confirmed by RPW based on the description in Wappes and Santos-Silva (2019).

***Clytusmarginicollis* Laporte & Gory, 1835**

**New records. Kings Co.**, Valleyfield, 46.1356°N, 62.7198°W, 12.VI–3.VII.2018 (2), 4.VI–3.VII.2019 (1), canopy trap in poplar (1), low traps (2) (3, AFC); New Harmony, 46.3914°N, 62.2021°W, 3.VII–13.VIII.2019, canopy trap in red maple (1, AFC). **Queens Co.**, Auburn, 46.2882°N, 62.9267°W, 13.VI–3.VII.2018 (2), 3.VII–2.VIII.2018 (1), 3.VII–14.VIII.2019 (1), canopy traps in poplar (4, AFC); Brookvale, 46.2920°N, 63.4051°W, 13.VI–3.VII.2018 (4), 3.VII–13.VIII.2019 (1), low traps (5, AFC).

**Distribution in Canada and Alaska.** ON, QC, NB, NS, **PE** (Bousquet et al. 2013).

***Neoclytusacuminatusacuminatus* (Fabricius, 1775)**

**New record. Queens Co.**, Brookvale, 46.2920°N, 63.4051°W, 2.VIII–13.IX.2018, low trap (1, AFC).

**Distribution in Canada and Alaska.**AB, SK, MB, ON, QC, NB, NS, **PE** (Bousquet et al. 2013).

***Sarosesthesfulminans* (Fabricius, 1775)**

**New record. Kings Co.**, New Harmony, 46.3914°N, 62.2021°W, 3.VII–13.VIII.2019, canopy trap in red maple (1, AFC).

**Distribution in Canada and Alaska.**MB, ON, QC, NB, NS, **PE** (Bousquet et al. 2013; Webster et al. 2022).

**Note**: Males of *S.fulminans* emit (*R*)-3-hydroxyhexan-2-one and (2 *S*, 3*R*)-hexanediol that attracts both sexes (Lacey et al. 2009). These aggregation pheromones were present in the multi-lure (hardwood blend) used to bait the Lindgren funnel traps in this study. Traps baited with these pheromones were also responsible for the first detections of *S.fulminans* in New Brunswick (Webster et al. 2012c) and Nova Scotia (Webster et al. 2022).

***Xylotrechusquadrimaculatus* (Haldeman, 1847)**

**New record. Queens Co.**, Brookvale, 46.2920°N, 63.4051°W, 3.VII–2.VIII.2018, low trap (1, RWC).

**Distribution in Canada and Alaska.**MB, ON, QC, NB, NS, **PE** (Bousquet et al. 2013).

***Molorchusbimaculatusbimaculatus* Say, 1824**

**New record. Kings Co.**, Valleyfield, 46.1356°N, 62.7198°W, 12.VI–3.VII.2018 (1), 3.VII–2.VIII.2018 (1), 3.VII–13.VIII.2019 (1), canopy traps in canopy of poplar (2), red maple (1) (3, AFC).

**Distribution in Canada and Alaska.**AB, SK, MB, ON, QC, NB, NS, **PE** (Bousquet et al. 2013).

##### Subfamily LAMIINAE Latreille, 1825

***Astyleiopusvariegatus* (Haldeman, 1847)**

**New record. Queens Co.**, Brookvale, 46.2920°N, 63.4051°W, 2.VIII–13.IX.2018, low trap (1, AFC).

**Distribution in Canada and Alaska.**AB, SK, MB, ON, QC, NB, NS, **PE** (Bousquet et al. 2013).

***Astylopsismacula* (Say, 1826)**

**New records. Kings Co.**, Valleyfield, 46.1356°N, 62.7198°W, 3.VII–2.VIII.2018 (3), 3.VII–13.VIII.2019 (1), canopy traps in red maple (2), in poplar (1), low trap (1) (4, AFC); New Harmony, 46.3914°N, 62.2021°W, 3.VII–13.VIII.2019, canopy trap in red maple (1, AFC). **Queens Co.**, Auburn, 46.2882°N, 62.9267°W, 3.VII–2.VIII.2018 (5), 3.VII–14.VIII.2019 (1), canopy traps in red maple (2), poplar (2), low traps (2) (5, AFC); Brookvale, 46.2920°N, 63.4051°W, 3.VII–2.VIII.2018, 3.VII–13.VIII.2019, low traps (2, AFC).

**Distribution in Canada and Alaska.** ON, QC, NB, NS, **PE** (Bousquet et al. 2013).

***Astylopsissexguttata* (Say, 1826)**

**New records. Kings Co.**, Valleyfield, 46.1356°N, 62.7198°W, 2.VIII–13.IX.2018 (2), 3.VII–13.VIII.2019 (2), canopy traps in poplar (1), in white spruce (1), in hemlock (1), low trap (1) (4, AFC). **Queens Co.**, Auburn, 46.2882°N, 62.9267°W, 3.VII–2.VIII.2018 (2), 2.VIII–13.IX.2018 (2), canopy traps in poplar (3), low trap (1) (4, AFC).

**Distribution in Canada and Alaska.**MB, ON, QC, NB, NS, **PE** (Bousquet et al. 2013).

***Graphisurusfasciatus* (DeGeer, 1775)**

**New records. Kings Co.**, Valleyfield, 46.1356°N, 62.7198°W, 3.VII–2.VIII.2018 (1), 2.VIII–13.IX.2018 (1), 3.VII–13.VIII.2019 (2), canopy traps in poplar (1), in white spruce (1), low traps (2) (4, AFC); New Harmony, 46.3914°N, 62.2021°W, 3.VII–13.VIII.2019 (2), 13.VIII–17.IX.2019 (1), canopy trap in red maple (1), low traps (2) (3 AFC). **Queens Co.**, Auburn, 46.2882°N, 62.9267°W, 2.VIII–13.IX.2018, canopy trap in poplar (1, AFC); Brookvale, 3.VII–2.VIII.2018, low trap (1, AFC).

**Distribution in Canada and Alaska.** ON, QC, NB, NS, **PE** (Bousquet et al. 2013).

***Hyperplatysmaculata* Haldeman, 1847**

**New records. Kings Co.**, Valleyfield, 46.1356°N, 62.7198°W, 3.VII–2.VIII.2018, canopy trap in red maple (2, AFC); New Harmony, 46.3914°N, 62.2021°W, 3.VII–13.VIII.2019, 13.VIII–17.IX.2019, canopy traps in sugar maple (2, AFC). **Queens Co.**, Auburn, 46.2882°N, 62.9267°W, 2.VIII–13.IX.2018 (1), 3.VII–14.VIII.2019 (2), canopy traps in poplar (3, AFC).

**Distribution in Canada and Alaska.**MB, ON, QC, NB, NS, **PE** (Bousquet et al. 2013).

***Sternidiusrusticus* (LeConte, 1852)**

**New record. Kings Co.**, Valleyfield, 46.1356°N, 62.7198°W, 4.VI–3.VII.2019, canopy trap in white spruce (1, AFC).

**Distribution in Canada and Alaska.**MB, ON, NB, NS, **PE** (Bousquet et al. 2013, 2017).

***Urgleptessignatus* (LeConte, 1852)**

**New records. Kings Co.**, Valleyfield, 46.1356°N, 62.7198°W, 3.VII–2.VIII.2018 (3), 2.VIII–13.IX.2018 (1), 3.VII–13.VIII.2019 (1), canopy traps in red maple (3), in poplar (1), low trap (1) (5, AFC); New Harmony, 46.3914°N, 62.2021°W, 3.VII–13.VIII.2019, canopy traps in red maple (1), in sugar maple (1) (2, AFC). **Queens Co.**, Auburn, 46.2882°N, 62.9267°W, 3.VII–2.VIII.2018 (1), 2.VIII–13.IX.2018 (2), canopy traps in poplar (3, AFC); Brookvale, 46.2920°N, 63.4051°W, 3.VII–2.VIII.2018 (5), 2.VIII–13.IX.2018 (2), 3.VII–13.VIII.2019 (2), canopy trap in white pine (1), low traps (8) (9, AFC).

**Distribution in Canada and Alaska.** ON, QC, NB, **PE** (Bousquet et al. 2013).

***Psenocerussupernotatus* (Say, 1823)**

**New records. Kings Co.**, Valleyfield, 46.1356°N, 62.7198°W, 3.VII–2.VIII.2018 (3), 2.VIII–13.IX.2018 (1), 3.VII–13.VIII.2019 (1), canopy traps in red maple (3), in poplar (1), in white spruce (1) (5, AFC); New Harmony, 46.3914°N, 62.2021°W, 3.VII–13.VIII.2019, canopy trap in sugar maple (1, AFC). **Queens Co.**, Auburn, 46.2882°N, 62.9267°W, 3.VII–14.VIII.2019, canopy trap in poplar (1, AFC); Brookvale, 46.2920°N, 63.4051°W, 3.VII–2.VIII.2018, 3.VII–13.VIII.2019, canopy trap in hemlock (1), low trap (1) (2, AFC).

**Distribution in Canada and Alaska.**MB, ON, QC, NB, NS, **PE** (Bousquet et al. 2013).

***Microgoesoculatus* (LeConte, 1862)**

**New records. Queens Co.**, Auburn, 46.2882°N, 62.9267°W, 3.VII–2.VIII.2018, canopy traps in poplar (1), in red maple (2) (3, AFC); Brookvale, 46.2920°N, 63.4051°W, 3.VII–2.VIII.2018, low trap (1, AFC).

**Distribution in Canada and Alaska.** ON, QC, NB, NS, **PE** (Bousquet et al. 2013).

***Monochamusmarmorator* Kirby, 1837**

**New records. Kings Co.**, Valleyfield, 46.1356°N, 62.7198°W, 3.VII–2.VIII.2018 (1), 2.VIII–13.IX.2018 (2), 3.VII–13.VIII.2019 (1), canopy trap in poplar (1) low traps (3) (4, AFC).

**Distribution in Canada and Alaska.**MB, ON, QC, NB, NS, **PE** (Bousquet et al. 2013).

***Pogonocheruspencillatus* LeConte, 1850**

**New records. Kings Co.**, Valleyfield, 46.1356°N, 62.7198°W, 12.VI–3.VII.2018, 7.V–4.VI.2019, canopy trap in poplar (1) low trap (1) (2, AFC). **Queens Co.**, Auburn, 46.2882°N, 62.9267°W, 13.VI–3.VII.2018 (2), 3.VII–2.VIII.2018 (1), 4.VI–3.VII.2019 (1), canopy traps in poplar (3) low trap (1) (4, AFC); Brookvale, 46.2920°N, 63.4051°W, 13.VI–3.VII.2018 (2), 3.VII–2.VIII.2018 (2), 7.V–4.VI.2019 (1), canopy trap in hemlock (1) low traps (4) (5, AFC).

**Distribution in Canada and Alaska.**AK, YT, NT, BC, AB, SK, MB, ON, QC, NB, NS, **PE**, LB, NF (Bousquet et al. 2013).

***Tetropspraeusta* (Linnaeus, 1758)**†

**New record. Queens Co.**, Brookvale, 46.2920°N, 63.4051°W, 13.VI–3.VII.2018 (1), 3.VII–2.VIII.2018 (1), low traps (2, AFC).

**Distribution in Canada and Alaska.**QC, NB, NS, **PE** (Bousquet et al. 2013; Webster et al. 2020).

#### Family MEGALOPODIDAE Latreille, 1802

One species of this family, *Zeugophoraabnormis* (LeConte), was previously reported from PE by Bousquet et al. (2013). This and three species new to PE were recorded in this study.

##### Subfamily ZEUGOPHORINAE Böving & Craighead, 1931

**Zeugophora (Zeugophora) puberula Crotch, 1873**

**New records. Kings Co.**, Valleyfield, 46.1356°N, 62.7198°W, 12.VI–3.VII.2018 (3), 4.VI–3.VII.2019 (1), canopy traps in poplar (2, AFC; 2, RWC). **Queens Co.**, Auburn, 46.2882°N, 62.9267°W, 13.VI–3.VII.2018 (5), 3.VII–2.VIII.2018 (8), canopy traps in poplar (6, AFC; 7, RWC).

**Distribution in Canada and Alaska.**MB, ON, QC, NB, NS, **PE** (Bousquet et al. 2013; Webster et al. 2016e, 2022).

**Zeugophora (Zeugophora) scutellaris Suffrian, 1840**†

**New record. Queens Co.**, Auburn, 46.2882°N, 62.9267°W, 3.VII–2.VIII.2018, canopy trap in poplar (1, RWC).

**Distribution in Canada and Alaska.**NT, BC, AB, SK, MB, ON, QC, NB, **PE** (Bousquet et al. 2013; Webster et al. 2016e).

**Zeugophora (Zeugophora) varians Crotch, 1873**

**New records. Queens Co.**, Auburn, 46.2882°N, 62.9267°W, 2.VIII–13.IX.2018, canopy traps in poplar (2, AFC); Brookvale, 3.VII–2.VIII.2018, low traps (2, AFC).

**Distribution in Canada and Alaska.**NT, BC, AB, SK, MB, ON, QC, NB, NS, **PE** (Bousquet et al. 2013).

#### Family ORSODACNIDAE C.G. Thomson, 1859

*Orsodacneatra* (Ahrens) is the only member of this family known from Canada and PE (Bousquet et al. 2013). This species was captured in Lindgren funnel traps at three of the four study sites.

#### Family CHRYSOMELIDAE Latreille, 1802

Bousquet et al. (2013) listed 93 species of Chrysomelidae from PE. Another two species were added to the provincial list by Webster et al. (2022). In this study we recorded 20 species of this family using Lindgren funnel traps. Only three of these are provincial records.

##### Subfamily CHRYSOMELINAE Latreille, 1802

**Calligrapha (Calligrapha) knabi W.J. Brown, 1940**

**New record. Queens Co.**, Brookvale, 46.2920°N, 63.4051°W, 13.VI–3.VII.2018 (1), 4.VI.3.VII.2019 (1), low traps (2, RWC).

**Distribution in Canada and Alaska.**MB, ON, QC. **PE** (Bousquet et al. 2013).

##### Subfamily GALERUCINAE Latreille, 1802

***Chaetocnemahortensis* (Geoffroy, 1785)**†

**New record. Queens Co.**, Brookvale, 46.2920°N, 63.4051°W, 13.VI–3.VII.2018, low trap (1, AFC).

**Distribution in Canada and Alaska.**BC, ON, NB, NS, **PE**, LB, NF (Pentinsaari et al. 2019; Webster et al. 2020).

##### Subfamily CRYPTOCEPHALINAE Gyllenhal, 1813

**Pachybrachis (Pachybrachis) obsoletus Suffrian, 1852**

**New record. Kings Co.**, Valleyfield, 46.1356°N, 62.7198°W, 3.VII–13.VIII.2019, canopy trap in poplar (1, AFC).

**Distribution in Canada and Alaska.**BC, AB, SK, MB, ON, QC, NB, **PE** (Bousquet et al. 2013,

#### Family NEMONYCHIDAE Bedel, 1882

We report this family for the first time for PE based on the two species listed below.

##### Subfamily CIMBERIDINAE Gozis, 1882

***Cimberiselongata* (LeConte, 1876)**

**New record. Queens Co.**, Brookvale, 46.2920°N, 63.4051°W, 13.VI–3.VII.2018 (1), 7.V–4.VI.2019 (1), 4.VI–3.VII.2019 (1), 3.VII–13.VIII.2019 (2), Canopy traps in white pine (4), low trap (1) (5, AFC).

**Distribution in Canada and Alaska.**BC, SK, MB, ON, QC, NB, NS, **PE** (Bousquet et al. 2013).

***Cimberispallipennis* (Blatchley, 1916)**

**New records. Kings Co.**, Valleyfield, 46.1356°N, 62.7198°W, 12.VI–3.VII.2018 (1), 7.V–4.VI.2019 (1), 4.VI–3.VII.2019 (2), canopy traps in poplar (4, AFC). **Queens Co.**, Auburn, 46.2882°N, 62.9267°W, 13.VI–3.VII.2018 (1), 7.V–4.VI.2019 (1), 3.VII–14.VIII.2019 (1), canopy traps in poplar (3, AFC); Brookvale, 46.2920°N, 63.4051°W, 7.V–4.VI.2019, low trap (1, AFC).

**Distribution in Canada and Alaska.**AB, QC, NB, NS, **PE** (Bousquet et al. 2013; Webster et al. 2016a).

#### Family ANTHRIBIDAE Billberg, 1820

*Trigonorhinussticticus* (Boheman) was the only species of Anthribidae reported from PE by Bousquet et al. (2013). Webster et al. (2020) later reported the adventive *Anthribusnebulosus* Forster for the first time for PE from specimens collected at the Auburn Woodlot in this study.

#### Family ATTELABIDAE Billberg, 1820

Bousquet et al. (2013) reported two species of this small family for PE. Here we report one additional species for the province. We did not capture the other two known species in this study.

##### Subfamily RHYNCHITINAE Gistel, 1848

***Temnoceruscyanellus* (LeConte, 1876)**

**New records. Kings Co.**, Valleyfield, 46.1356°N, 62.7198°W, 12.VI–3.VII.2018, canopy trap in poplar (1, AFC). **Queens Co.**, Brookvale, 46.2920°N, 63.4051°W, 3.VII–2.VIII.2018, low trap (1, AFC).

**Distribution in Canada and Alaska.**NT, AB, SK, MB, ON, QC, NB, NS, **PE**, NF (Bousquet et al. 2013).

#### Family BRENTIDAE Billberg, 1823

Bousquet et al. (2013) reported three species of Brentidae from PE. Here we report one additional species. We did not collect examples of the three previously known species in this study.

##### Subfamily APIONINAE Schönherr, 1823

***Betulapionsimilewalshii* (J.B. Smith, 1884)**

**New records. Kings Co.**, Valleyfield, 46.1356°N, 62.7198°W, 12.VI–3.VII.2018 (3), 7.V–4.VI.2019 (1), canopy traps in red maple (3), low trap (1) (3, AFC); New Harmony, 46.3914°N, 62.2021°W, 8.V–5.VI.2019, 13.VIII–17.IX.2019, canopy trap in sugar maple (1), low trap (1) (2, AFC). **Queens Co.**, Auburn, 46.2882°N, 62.9267°W, 13.VI–3.VII.2018 (1), 2.VIII–13.IX.2018 (1), canopy traps in poplar (2, AFC); Brookvale, 46.2920°N, 63.4051°W, 13.VI–3.VII.2018 (2), 3.VII–2.VIII.2018 (1) low traps (3, AFC).

**Distribution in Canada and Alaska.**BC, AB, SK, MB, ON, QC, NB, NS, **PE**, NF (Bousquet et al. 2013).

#### Family DRYOPHTHORIDAE Schönherr, 1825

Bousquet et al. (2013) reported four species of this family for PE. The only species of this family we recorded is a new record, reported below.

##### Subfamily DRYOPHTHORINAE Schönherr, 1825

***Dryophthorusamericanus* Bedel, 1885**

**New records. Kings Co.**, Valleyfield, 46.1356°N, 62.7198°W, 3.VII–2.VIII.2018 (2), 3.VII–13.VIII.2019 (1), low traps (3, AFC); New Harmony, 46.3914°N, 62.2021°W, 3.VII–13.VIII.2019, low trap (1, AFC). **Queens Co.**, Auburn, 46.2882°N, 62.9267°W, 3.VII–2.VIII.2018 (4), 3.VII–14.VIII.2019 (1), low traps (5, AFC); Brookvale, 46.2920°N, 63.4051°W, 3.VII–2.VIII.2018, 3.VII–13.VIII.2019, low traps (2, AFC).

**Distribution in Canada and Alaska.**BC, AB, SK, MB, ON, QC, NB, NS, **PE**, NF (Bousquet et al. 2013).

#### Family BRACHYCERIDAE Billberg, 1820

*Notarispuncticollis* (LeConte) is the only member of this family listed for PE by Bousquet et al. (2013). We did not record this species at any of the study sites.

#### Family CURCULIONIDAE Latreille, 1802

Bousquet et al. (2013) listed 99 species of Curculionidae for PE. In this study, we recorded 84 species of this family. Four of these, the adventive *Polydrususimpressifrons* Gyllenhal, *Hylastesopacus* Erichson, *Xyleborinussaxesenii* (Ratzeburg), and *X.germanus* (Blandford) were previously reported as new to PE by Webster et al. (2020). Here, we report an additional 36 new records for PE.

##### Subfamily CURCULIONINAE Latreille, 1802

**Anthonomus (Tachypterellus) quadrigibbus Say, 1832**

**New records. Kings Co.**, Valleyfield, 46.1356°N, 62.7198°W, 12.VI–3.VII.2018, canopy trap in poplar (1, AFC). **Queens Co.**, Auburn, 46.2882°N, 62.9267°W, 13.VI–3.VII.2018, canopy trap in poplar (1, AFC); Brookvale, 46.2920°N, 63.4051°W, 12.VI–3.VII.2018, low trap (1, AFC).

**Distribution in Canada and Alaska.**BC, AB, SK, MB, ON, QC, NB, NS, **PE** (Bousquet et al. 2013).

***Pseudanthonomusrufulus* Dietz, 1891**

**New records. Kings Co.**, Valleyfield, 46.1356°N, 62.7198°W, 13.VIII–17.IX.2019, canopy trap in poplar (1, AFC); New Harmony, 46.3914°N, 62.2021°W, 3.VII–13.VIII.2019, canopy trap in red maple (1, RWC).

**Distribution in Canada and Alaska.**MB, QC, **PE** (Bousquet et al. 2013).

***Pseudanthonomusseriesetosus* Dietz, 1891**

**New records. Kings Co.**, Valleyfield, 46.1356°N, 62.7198°W, 7.V–4.VI.2019, canopy trap in white spruce (1, RWC). New Harmony, 46.3914°N, 62.2021°W, 5.VI–3.VII.2019, canopy trap in sugar maple (1, AFC).

**Distribution in Canada and Alaska.** ON, QC, NB, NS, **PE** (Bousquet et al. 2013).

***Dorytomusparvicollis* Casey, 1892**

**New records. Kings Co.**, Valleyfield, 46.1356°N, 62.7198°W, 12.VI–3.VII.2018, low trap (1, AFC). **Queens Co.**, Auburn, 46.2882°N, 62.9267°W, 13.VI–3.VII.2018, canopy trap in red maple (1, AFC).

**Distribution in Canada and Alaska.**AK, BC, AB, SK, MB, ON, QC, NB, NS, **PE** (Bousquet et al. 2013).

***Orchestestestaceus* (O.F. Müller, 1776)**

**New records. Kings Co.**, Valleyfield, 46.1356°N, 62.7198°W, 12.VI–3.VII.2018, canopy trap in poplar (1, AFC). **Queens Co.**, Auburn, 46.2882°N, 62.9267°W, 13.VI–3.VII.2018, low trap (1, AFC); Brookvale, 46.2920°N, 63.4051°W, 13.VI–3.VII.2018, low trap (1, AFC).

**Distribution in Canada and Alaska.**AK, BC, AB, SK, MB, ON, QC, NB, NS, **PE**, NF (Bousquet et al. 2013).

***Tachyergesephippiatus* (Say, 1832)**

**New records. Kings Co.**, Valleyfield, 46.1356°N, 62.7198°W, 12.VI–3.VII.2018 (3), 4.VI–3.VII.2019 (1), canopy traps in poplar, in white spruce (1) (4, AFC; 1, RWC). **Queens Co.**, Auburn, 46.2882°N, 62.9267°W, 13.VI–3.VII.2018 (1), 3.VII–2.VIII.2018 (1), canopy traps in poplar (2, AFC).

**Distribution in Canada and Alaska.**BC, AB, SK, MB, ON, QC, NB, NS, **PE** (Bousquet et al. 2013).

***Tachyergessalicis* (Linnaeus, 1758)**

**New records. Kings Co.**, Valleyfield, 46.1356°N, 62.7198°W, 12.VI–3.VII.2018, canopy trap in poplar (1, AFC). **Queens Co.**, Brookvale, 46.2920°N, 63.4051°W, 13.VI–3.VII.2018, low trap (1, AFC).

**Distribution in Canada and Alaska.**NT, BC, AB, SK, MB, ON, QC, NB, NS, **PE** (Bousquet et al. 2013).

##### Subfamily BARIDINAE Schönherr, 1836

***Stethobarisovata* (LeConte, 1868)**

**New record. Queens Co.**, Brookvale, 46.2920°N, 63.4051°W, 3.VII–3.VII.2019, low trap (1), white panel trap (1) (2, RWC).

**Distribution in Canada and Alaska.**NT, BC, AB, SK, MB, ON, QC, NB, NS, **PE** (Bousquet et al. 2013).

##### Subfamily CONODERINAE Schönherr, 1833

***Acoptussuturalis* LeConte, 1876**

**New records. Kings Co.**, Valleyfield, 46.1356°N, 62.7198°W, 12.VI–3.VII.2018 (1), 3.VII–2.VIII.2018 (1), 4.VI–3.VII.2019 (1), canopy traps in red maple (2), in white spruce (1) (3, AFC); New Harmony, 46.3914°N, 62.2021°W, 3.VII–13.VIII.2019, low trap (1, AFC). **Queens Co.**, Auburn, 46.2882°N, 62.9267°W, 13.VI–3.VII.2018, 4.VI–3.VII.2019, canopy traps in poplar (2, AFC); Brookvale, 46.2920°N, 63.4051°W, 13.VI–3.VII.2018 (2), 3.VII–2.VIII.2018 (1), 4.VI–3.VII.2019 (1), low traps (4, AFC).

**Distribution in Canada and Alaska.** ON, QC, NB, NS, **PE** (Bousquet et al. 2013).

##### Subfamily COSSONINAE Schönherr, 1825

***Phloeophagusapionides* Horn, 1873**

**New records. Kings Co.**, Valleyfield, 46.1356°N, 62.7198°W, 2.VIII–13.IX.2018, canopy trap in poplar (1, AFC); New Harmony, 46.3914°N, 62.2021°W, 3.VII–13.VIII.2019, canopy trap in red maple (1, AFC).

**Distribution in Canada and Alaska.** ON, QC, NB, NS, **PE** (Bousquet et al. 2013).

***Himatiumerrans* LeConte, 1876**

**New record. Queens Co.**, Auburn, 46.2882°N, 62.9267°W, 3.VII–2.VIII.2018, low traps (4, AFC).

**Distribution in Canada and Alaska.**QC, NB, NS, **PE** (Bousquet et al. 2013).

***Rhyncolusmacrops* Buchanan, 1946**

**New records. Kings Co.**, Valleyfield, 46.1356°N, 62.7198°W, 12.VI–3.VII.2018 (2), 3.VII–13.VIII.2019 (1), low traps (3, AFC); New Harmony, 46.3914°N, 62.2021°W, 5.VI–3.VII.2019, low trap (1, AFC). **Queens Co.**, Auburn, 46.2882°N, 62.9267°W, 13.VI–3.VII.2018 (2), 3.VII–2.VIII.2018 (1), 4.VI–3.VII.2019 (1), low traps (4, AFC); Brookvale, 46.2920°N, 63.4051°W, 13.VI–3.VII.2018 (1), 3.VII–2.VIII.2018 (1), 4.VI–3.VII.2019 (1), low traps (3, AFC).

**Distribution in Canada and Alaska.**AK, BC, ON, QC, NB, NS, **PE** (Bousquet et al. 2013).

##### Subfamily MESOPTILIINAE Lacordaire, 1863

***Magdalisalutacea* LeConte, 1878**

**New record. Queens Co.**, Brookvale, 46.2920°N, 63.4051°W, 3.VII–2.VIII.2018, low traps (2, AFC).

**Distribution in Canada and Alaska.**AK, YT, NT, BC, AB, SK, QC, NB, **PE** (Bousquet et al. 2013).

***Magdalishispoides* LeConte, 1876**

**New records. Queens Co.**, Auburn, 46.2882°N, 62.9267°W, 13.VI–3.VII.2018 (2), canopy traps in poplar (2, AFC); Brookvale, 46.2920°N, 63.4051°W, 13.VI–3.VII.2018 (3), 3.VII–2.VIII.2018 (3), low traps (6, AFC).

**Distribution in Canada and Alaska.**YT, BC, AB, ON, QC, NB, **PE**, NF (Bousquet et al. 2013).

##### Subfamily MOLYTINAE Schönherr, 1823

***Pissodesaffinis* Randall, 1838**

**New records. Kings Co.**, Valleyfield, 46.1356°N, 62.7198°W, 12.VI–3.VII.2018 (2), 4.VI–3.VII.2019 (1), canopy trap in red maple (1), low traps (2) (3, AFC). **Queens Co.**, Auburn, 46.2882°N, 62.9267°W, 13.VI–3.VII.2018, canopy traps in poplar (2, AFC); Brookvale, 46.2920°N, 63.4051°W, 13.VI–3.VII.2018 (3), 4.VI–3.VII.2019 (2), canopy trap in hemlock (1) low traps (4) (5, AFC).

**Distribution in Canada and Alaska.**NT, BC, AB, SK, MB, ON, QC, NB, NS, **PE**, NF (Bousquet et al. 2013).

***Pissodesrotundatus* LeConte, 1876**

**New records. Kings Co.**, Valleyfield, 46.1356°N, 62.7198°W, 4.VI–3.VII.2019, canopy traps in poplar (1), in white spruce (1) (2, AFC). **Queens Co.**, Auburn, 46.2882°N, 62.9267°W, 3.VII–2.VIII.2018, canopy trap in poplar (1, AFC); Brookvale, 46.2920°N, 63.4051°W, 3.VII–2.VIII.2018, low traps (3, AFC).

**Distribution in Canada and Alaska.**AK, YT, NT, BC, AB, SK, MB, ON, QC, NB, NS, **PE**, NF (Bousquet et al. 2013).

***Pissodessimilis* Hopkins, 1911**

**New records. Kings Co.**, Valleyfield, 46.1356°N, 62.7198°W, 12.VI–3.VII.2018 (1), 3.VII–2.VIII.2018 (1), 3.VII–13.VIII.2019 (1), low traps (3, AFC); New Harmony, 46.3914°N, 62.2021°W, 5.VI–3.VII.2019, canopy trap in white pine (1, AFC). **Queens Co.**, Brookvale, 46.2920°N, 63.4051°W, 13.VI–3.VII.2018, low traps (3, AFC).

**Distribution in Canada and Alaska.**BC, AB, ON, QC, NB, NS, **PE**, NF (Bousquet et al. 2013).

##### Subfamily SCOLYTINAE Latreille, 1804

***Gnathotrichusmateriarius* (Fitch, 1858)**

**New records. Kings Co.**, Valleyfield, 46.1356°N, 62.7198°W, 12.VI–3.VII.2018, canopy trap in hemlock (1, AFC). **Queens Co.**, Auburn, 46.2882°N, 62.9267°W, 13.VI–3.VII.2018, 4.VI–3.VII.2019, low traps (2, AFC); Brookvale, 46.2920°N, 63.4051°W, 13.VI–3.VII.2018 (2), 4.VI–3.VII.2019 (1), 3.VII–13.VIII.2019 (1), low traps (4, AFC).

**Distribution in Canada and Alaska.**SK, MB, ON, QC, NB, NS, **PE** (Bousquet et al. 2013).

***Conophthorusconiperda* (Schwarz, 1895)**

**New record. Queens Co.**, Brookvale, 46.2920°N, 63.4051°W, 4.VI–3.VII.2019, low trap (1, AFC).

**Distribution in Canada and Alaska.** ON, QC, NB, NS, **PE** (Bousquet et al. 2013).

***Monarthrummali* (Fitch, 1855)**

**New records. Kings Co.**, Valleyfield, 46.1356°N, 62.7198°W, 12.VI–3.VII.2018, 4.VI–3.VII.2019, canopy trap in white spruce (1), low trap (1) (2, AFC); New Harmony, 46.3914°N, 62.2021°W, 5.VI–3.VII.2019, canopy trap in sugar maple (1, AFC). **Queens Co.**, Brookvale, 46.2920°N, 63.4051°W, 13.VI–3.VII.2018 (2), 4.VI–3.VII.2019 (1), low traps (3, AFC).

**Distribution in Canada and Alaska.** ON, QC, NB, NS, **PE** (Bousquet et al. 2013).

***Pityophthorus* (*Pityophthoru* s) *balsameus* Blackman, 1922**

**New records. Kings Co.**, Valleyfield, 46.1356°N, 62.7198°W, 3.VII–13.VIII.2019, canopy trap in poplar (1, AFC). **Queens Co.**, Brookvale, 46.2920°N, 63.4051°W, 7.V–4.VI.2019, canopy trap in hemlock (1, RWC).

**Distribution in Canada and Alaska.**NT, ON, QC, NB, NS, **PE** (Bousquet et al. 2013).

***Pityophthorus* (*Pityophthoru* s) *carinatuscarinatus* Bright, 1978**

**New record. Kings Co.**, Valleyfield, 46.1356°N, 62.7198°W, 7.V–4.VI.2019, canopy trap in poplar (1), low trap (1) (1, AFC: 1, RWC).

**Distribution in Canada and Alaska.**QC, NB, NS, **PE** (Bousquet et al. 2013).

***Pityophthorus* (*Pityophthoru* s) *concavus* Blackman, 1928**

**New record. Queens Co.**, Brookvale, 46.2920°N, 63.4051°W, 13.VI–3.VII.2018, low traps (2, AFC).

**Distribution in Canada and Alaska.** ON, QC, NB, NS, **PE** (Bousquet et al. 2013).

***Pityophthorus* (*Pityophthoru* s) *opaculus* LeConte, 1878**

**New records. Queens Co.**, Auburn, 46.2882°N, 62.9267°W, 13.VI–3.VII.2018 (1), 4.VI–3.VII.2019 (1), canopy traps in poplar (2, AFC); Brookvale, 46.2920°N, 63.4051°W, 7.V–4.VI.2019 (2), 3.VII–13.VIII.2019 (1), canopy traps in hemlock (3, AFC).

**Distribution in Canada and Alaska.** (Bousquet et al. 2013).

***Pityophthorus* (*Pityophthoru* s) *puberulus* (LeConte, 1868)**

**New records. Kings Co.**, Valleyfield, 46.1356°N, 62.7198°W, 12.VI–3.VII.2018, canopy trap in hemlock (4, AFC). **Queens Co.**, Auburn, 46.2882°N, 62.9267°W, 13.VI–3.VII.2018, canopy trap in poplar (1, AFC); Brookvale, 46.2920°N, 63.4051°W, 13.VI–3.VII.2018, low traps (2, AFC).

**Distribution in Canada and Alaska.** ON, QC, NB, NS, **PE**, NF (Bousquet et al. 2013).

***Pityophthorus* (*Pityophthoru* s) *ramiperda* Swaine, 1917**

**New records. Kings Co.**, New Harmony, 46.3914°N, 62.2021°W, 5.VI–3.VII.2019, canopy trap in red maple (1), low trap (1) (1, AFC; 1, RWC). **Queens Co.**, Brookvale, 46.2920°N, 63.4051°W, 7.V–4.VI–2019 (1), 4.VI–3.VII.2019 (2), 3.VII–13.VIII.2019 (1), low traps (1, AFC; 3, RWC).

**Distribution in Canada and Alaska.** ON, QC, NB, NS, **PE** (Bousquet et al. 2013).

***Cryphalusruficollisruficollis* Hopkins, 1915**

**New records. Kings Co.**, Valleyfield, 46.1356°N, 62.7198°W, 12.VI–3.VII.2018 (1), 5.VI–3.VII.2019 (1), canopy trap in hemlock (1), low traps (2) (3, AFC); New Harmony, 46.3914°N, 62.2021°W, 5.VI–3.VII.2019, low trap (1) (1, AFC). **Queens Co.**, Auburn, 46.2882°N, 62.9267°W, 13.VI–3.VII.2018, 4.VI–3.VII.2019 (1), canopy traps in red maple (1), in poplar (1), low trap (1) (3, AFC); Brookvale, 46.2920°N, 63.4051°W, 13.VI–3.VII.2018, 7.V–4.VI.2019 (1), canopy trap in hemlock (1), low traps (4, AFC).

**Distribution in Canada and Alaska.**AK, YT, BC, AB, MB, ON, QC, NB, NS, **PE**, NF (Bousquet et al. 2013).

***Dryocoetesbetulae* Hopkins, 1894**

**New records. Kings Co.**, Valleyfield, 46.1356°N, 62.7198°W, 4.VI–3.VII.2019, white panel trap (1, RWC). **Queens Co.**, Brookvale, 46.2920°N, 63.4051°W, 13.VI–3.VII.2018, low trap (1, AFC).

**Distribution in Canada and Alaska.**BC, AB, ON, QC, NB, NS, **PE**, NF (Bousquet et al. 2013).

***Lymantordecipiens* (LeConte, 1878)**

**New records. Kings Co.**, Valleyfield, 46.1356°N, 62.7198°W, 12.VI–3.VII.2018, low trap (1, AFC); New Harmony, 46.3914°N, 62.2021°W, 5.VI–3.VII.2019 (2), 3.VII–13.VIII.2019 (1), low traps (3) (3, AFC). **Queens Co.**, Auburn, 46.2882°N, 62.9267°W, 13.VI–3.VII.2018 (2), 3.VII–2.VIII.2018 (1), 3.VII–14.VIII.2019 (2), canopy trap in poplar (1), low traps (4) (5, AFC); Brookvale, 46.2920°N, 63.4051°W, 13.VI–3.VII.2018, 4.VI–3.VII.2019, low traps (3, AFC).

**Distribution in Canada and Alaska.** ON, QC, NB, NS, **PE** (Bousquet et al. 2013).

***Ips grandicollis* (Eichhoff, 1868)**

**New records. Kings Co.**, Valleyfield, 46.1356°N, 62.7198°W, 12.VI–3.VII.2018 (3), 3.VII–2.VIII.2018 (3), 4.VI–3.VII.2019 (2) canopy traps in red maple (3), in hemlock (1), in poplar (2), low trap (2) (8, AFC); New Harmony, 46.3914°N, 62.2021°W, 3.VII–13.VIII.2019, low trap (1, AFC). **Queens Co.**, Brookvale, 46.2920°N, 63.4051°W, 3.VII–2.VIII.2018 (2), 4.VI–3.VII.2019 (1), 3.VII–13.VIII.2019 (1), canopy trap in white pine (1) low traps (3) (4, AFC).

**Distribution in Canada and Alaska.**MB, ON, QC, NB, NS, **PE** (Bousquet et al. 2013; Webster et al. 2016a).

***Ips perroti* Swaine, 1915**

**New records. Kings Co.**, Valleyfield, 46.1356°N, 62.7198°W, 7.V–4.VI.2019 (2), 4.VI–3.VII.2019 (1), canopy traps in poplar (3, AFC). **Queens Co.**, Auburn, 46.2882°N, 62.9267°W, 7.V–4.VI.2019, canopy trap in poplar (1, AFC); Brookvale, 46.2920°N, 63.4051°W, 13.VI–3.VII.2018 (2), 3.VII–13.VII.2019 (1), low traps (3, AFC).

**Distribution in Canada and Alaska.**BC, AB, SK, MB, ON, QC, NB, NS, **PE** (Bousquet et al. 2013).

***Orthotomicuslatidens* (LeConte, 1874)**

**New records. Kings Co.**, Valleyfield, 46.1356°N, 62.7198°W, 4.VI–3.VII.2019, low trap (1, AFC). **Queens Co.**, Brookvale, 46.2920°N, 63.4051°W, 4.VI–3.VII.2019, canopy trap in white pine (1) low trap (1) (2, AFC).

**Distribution in Canada and Alaska.**YT, BC, AB, SK, ON, QC, NB, NS, **PE** (Bousquet et al. 2013).

***Pityogeneshopkinsi* Swaine, 1915**

**New record. Queens Co.**, Brookvale, 46.2920°N, 63.4051°W, 4.VI–3.VII.2019 (1), 3.VII–13.VIII.2019 (1), canopy trap in white pine (1) low trap (1) (2, AFC).

**Distribution in Canada and Alaska.**SK, MB, ON, QC, NB, NS, **PE**, NF (Bousquet et al. 2013).

***Phloeotribuspiceae* Swaine, 1911**

**New record. Queens Co.**, Brookvale, 46.2920°N, 63.4051°W, 4.VI–3.VII.2019, canopy trap in hemlock (1, AFC).

**Distribution in Canada and Alaska.**AK, YT, NT, BC, AB, MB, ON, QC, NB, NS, **PE** (Bousquet et al. 2013).

***Scolytuspiceae* (Swaine, 1910)**

**New record. Kings Co.**, Valleyfield, 46.1356°N, 62.7198°W, 12.VI–3.VII.2018, canopy trap in poplar (1, AFC).

**Distribution in Canada and Alaska.**AK, YT, NT, BC, AB, SK, MB, ON, QC, NB, NS, **PE**, LB, NF (Bousquet et al. 2013).

***Anisandrussayi* (Hopkins, 1910)**

**New records. Kings Co.**, Valleyfield, 46.1356°N, 62.7198°W, 12.VI–3.VII.2018 (2), 4.VI–3.VII.2019 (1), low traps (3, AFC); New Harmony, 46.3914°N, 62.2021°W, 5.VI–3.VII.2019, low trap (1, AFC). **Queens Co.**, Auburn, 46.2882°N, 62.9267°W, 13.VI–3.VII.2018 (2), 4.VI–3.VII.2019 (1), canopy traps in red maple (1), in poplar (1), low trap (1) (3, AFC); Brookvale, 46.2920°N, 63.4051°W, 13.VI–3.VII.2018 (1), 4.VI–3.VII.2019 (1), low traps (2, AFC).

**Distribution in Canada and Alaska.** ON, QC, NB, NS, **PE** (Bousquet et al. 2013).

### ﻿Checklist of the Coleoptera of Prince Edward Island, Canada

The classification of the family-group taxa used in this checklist follows Bouchard et al. (2011), except for the Hydrophiloidea, which follows Short and Fikáĉek (2013) and the Aleocharinae that follows Klimaszewski et al. (2018). Synonyms and changes to the genus a species was included in proposed after the publication of Bousquet et al. (2013) are included in checklist (species or genus name indented). The order used is phylogenetic for superfamilies, families, and subfamilies starting with the accepted most basal-grade taxa, and is alphabetic for supertribes, tribes, and subtribes. Genera, subgenera, and species are listed alphabetically. The species included in this checklist are based on PE records contained in Bousquet et al. (2013), records published after that checklist, and new records included in this publication.

An asterisk [*] after a species name indicates that the taxon is Holarctic, a dagger [†] denotes an adventive species in North America, a double dagger [‡] indicates that the status is uncertain and that the species could be either Holarctic or adventive in North America. Species with a bullet point [●] after the name were recorded in this study; those that are also in bold are newly recorded for PE.

**Order COLEOPTERA**

**Suborder ADEPHAGA**

**Family GYRINIDAE Latreille, 1810**

**Subfamily GYRININAE Latreille, 1810**

**Tribe Enhydrusini Régimbart, 1882**

Subtribe Dineutina Desmarest, 1851

*Dineutusassimilis* (Kirby, 1837)

*Dineutushornii* Roberts, 1895

*Dineutusnigrior* Roberts, 1895

**Tribe Gyrinini Latreille, 1810**

Subtribe Gyrinina Latreille, 1810

Gyrinus (Gyrinus) affinis Aubé, 1838

Gyrinus (Gyrinus) aquiris LeConte, 1868

Gyrinus (Gyrinus) bifarius Fall, 1922

Gyrinus (Gyrinus) confinis LeConte, 1868

Gyrinus (Gyrinus) latilimbus Fall, 1922

Gyrinus (Gyrinus) fraternus Couper, 1865

Gyrinus (Gyrinus) lecontei Fall, 1922

Gyrinus (Gyrinus) pugionis Fall, 1922

Gyrinus (Gyrinus) sayi Aubé, 1838

**Family CARABIDAE Latreille, 1810**

**Subfamily NEBRIINAE Laporte, 1834**

**Tribe Nebriini Laporte, 1834**

Nebria (Reductonebria) pallipes Say, 1823

**Tribe Notiophilini Motschulsky, 1850**

*Notiophilusaeneus* (Herbst, 1806)

*Notiophilusbiguttatus* (Fabricius, 1779)†

*Notiophiluspalustris* (Duftschmid, 1812)†

*Notiophilussemistriatus* Say, 1823

**Subfamily CICINDELINAE Latreille, 1802**

**Tribe Cicindelini Latreille, 1802**

Subtribe Cicindelina Latreille, 1802

Cicindela (Cicindela) duodecimguttata Dejean, 1825

Cicindela (Cicindela) hirticollis rhodensis Calder, 1916

Cicindela (Cicindela) longilabris longilabris Say, 1824

Cicindela (Cicindela) repanda repanda Dejean, 1825

Cicindela (Cicindela) repanda novascotiae Vaurie, 1951

Cicindela (Cicindela) tranquebarica tranquebarica Herbst, 1806

**Subfamily CARABINAE Latreille, 1802**

**Tribe Carabini Latreille, 1802**

Calosoma (Calosoma) frigidum Kirby, 1837

Calosoma (Chrysostigma) calidum (Fabricius, 1775)

Carabus (Archicarabus) nemoralis nemoralis O. F. Müller, 1764†

Carabus (Carabus) granulatus granulatus Linnaeus, 1758†●

Carabus (Hemicarabus) serratus Say, 1823

Carabus (Homoeocarabus) maeander maeander Fischer von Waldheim, 1820

**Tribe Cychrini Perty, 1830**

*Sphaeroderusstenostomuslecontei* Dejean, 1826

**Subfamily LORICERINAE Bonelli, 1810**

**Tribe Loricerini Bonelli, 1810**

Loricera (Loricera) pilicornis pilicornis (Fabricius, 1775)*

**Subfamily OMOPHRONINAE Bonelli, 1810**

**Tribe Omophronini Bonelli, 1810**

*Omophronamericanum* Dejean, 1831

*Omophrontessellatum* Say, 1823

**Subfamily ELAPHRINAE Latreille, 1802**

**Tribe Elaphrini Latreille, 1802**

*Blethisahudsonica* Casey, 1924

*Blethisaquadricollis* Haldeman, 1847

Elaphrus (Elaphrus) americanus americanus Dejean, 1831

Elaphrus (Elaphrus) californicus Mannerheim, 1843

Elaphrus (Neoelaphrus) clairvillei Kirby, 1837

Elaphrus (Neoelaphrus) olivaceus LeConte, 1863

**Subfamily SCARITINAE Bonelli, 1810**

**Tribe Clivinini Rafinesque, 1815**

Subtribe Clivinina Rafinesque, 1815

Clivina (Clivina) fosser fosser (Linnaeus, 1758)†●

**Tribe Dyschriini Kolbe, 1880**

*Dyschiriusdejeanii* Putzeys, 1846●

*Dyschiriusglobulosus* (Say, 1823)

*Dyschiriussellatus* LeConte, 1857

*Dyschiriussetosus* LeConte, 1857

*Dyschiriussphaericollis* (Say, 1823)

**Subfamily BROSCINAE Hope, 1838**

**Tribe Broscini Hope, 1838**

Subtribe Broscina Hope, 1838

*Broscuscephalotes* (Linnaeus, 1758)†

**Subfamily TRECHINAE Bonelli, 1810**

**Tribe Bembidiini Stephens, 1827**

Subtribe Bembidiina Stephens, 1827

*Amerizuswingatei* (Bland, 1864)

Bembidion (Bembidion) quadrimaculatum oppositum Say, 1823

Bembidion (Bracteon) inaequale Say, 1823

Bembidion (Diplocampa) transparens transparens (Gebler, 1830)*

Bembidion (Eupetedromus) incrematum LeConte, 1860*

Bembidion (Furcacampa) mimus Hayward, 1897

Bembidion (Furcacampa) versicolor (LeConte, 1847)

Bembidion (Hirmoplataphus) nigrum Say, 1823

Bembidion (Hydrium) nitidum (Kirby, 1837)

Bembidion (Metallina) properans (Stephens, 1828)†●

Bembidion (Notaphus) constrictum (LeConte, 1847)

Bembidion (Notaphus) contractum Say, 1823

Bembidion (Notaphus) nigripes (Kirby, 1837)*

Bembidion (Notaphus) patruele Dejean, 1831

Bembidion (Ocydromus) scopulinum (Kirby, 1837)*

Bembidion (Peryphanes) stephensii Crotch, 1866†

Bembidion (Peryphus) bruxellense Wesmael, 1835†

Bembidion (Peryphus) obscurellum obscurellum (Motschulsky, 1845)*

Bembidion (Peryphus) petrosum petrosum Gebler, 1833*

Bembidion (Peryphus) sejunctum sejunctum Casey, 1918

Bembidion (Peryphus) tetracolum tetracolum Say, 1823†

Bembidion (Phyla) obtusum Audinet-Serville, 1821†

Bembidion (Semicampa) muscicola Hayward, 1897

Bembidion (Trepanedoris) concretum Casey, 1918

Bembidion (Trepanedoris) fortestriatum (Motschulsky, 1845)

Bembidion (Trepanedoris) frontale (LeConte, 1847)

Subtribe Tachyina Motschulsky, 1862

Elaphropus (Barytachys) incurvus (Say, 1830)

**Tachyta (Tachyta) angulata Casey, 1918**●

**Tribe Trechini Bonelli, 1810**

*Blemusdiscusdiscus* (Fabricius, 1792)†

Trechus (Trechus) apicalis Motschulsky, 1845*

Trechus (Trechus) rubens (Fabricius, 1792)†

**Subfamily PATROBINAE Kirby, 1837**

**Tribe Patrobini Kirby, 1837**

Subtribe Patrobina Kirby, 1837

*Patrobuslongicornis* (Say, 1823)

**Subfamily HARPALINAE Bonelli, 1810**

**Tribe Chlaeniini Brullé, 1834**

Chlaenius (Agostenus) niger Randall, 1838

Chlaenius (Brachylobus) lithophilus Say, 1823

Chlaenius (Chlaeniellus) pensylvanicus pensylvanicus Say, 1823

Chlaenius (Chlaenius) sericeus (Forster, 1771)

**Tribe Harpalini Bonelli, 1810**

Subtribe Anisodactylina Lacordaire, 1854

Anisodactylus (Anadaptus) sanctaecrucis (Fabricius, 1798)

Anisodactylus (Anisodactylus) harrisii LeConte, 1863

Anisodactylus (Anisodactylus) kirbyi Lindroth, 1953

Anisodactylus (Anisodactylus) nigerrimus (Dejean, 1831)

Anisodactylus (Anisodactylus) nigrita Dejean, 1829

Anisodactylus (Gynandrotarsus) rusticus (Say, 1823)

Notiobia (Anisotarsus) terminata (Say, 1823)

*Xestonotuslugubris* (Dejean, 1829)

Subtribe Harpalina Bonelli, 1810

Harpalus (Harpalus) affinis (Schrank, 1781)†

Harpalus (Harpalus) herbivagus Say, 1823

Harpalus (Harpalus) plenalis Casey, 1914●

Harpalus (Harpalus) rubripes (Duftschmid, 1812)†

Harpalus (Harpalus) somnulentus Dejean, 1829

Harpalus (Opadius) fulvilabris Mannerheim, 1853

Harpalus (Opadius) laevipes Zetterstedt, 1828*

Harpalus (Opadius) laticeps LeConte, 1850

Harpalus (Pseudophonus) pensylvanicus (DeGeer, 1774)

Harpalus (Pseudophonus) rufipes (DeGeer, 1774)†

*Ophonuspuncticeps* Stephens, 1828†

Subtribe Stenolophina Kirby, 1837

Acupalpus (Acupalpus) canadensis Casey, 1924

Acupalpus (Acupalpus) carus (LeConte, 1863)

Acupalpus (Acupalpus) pumilus Lindroth, 1968

Acupalpus (Tachistodes) pauperculus Dejean, 1829

*Agonoleptusconjunctus* (Say, 1823)

Bradycellus (Catharellus) lecontei Csiki, 1932

Bradycellus (Lipalocellus) nigrinus (Dejean, 1829)

Bradycellus (Stenocellus) neglectus (LeConte, 1847)●

Bradycellus (Triliarthrus) lugubris (LeConte, 1847)

Dicheirotrichus (Trichocellus) cognatus (Gyllenhal, 1827)*

Stenolophus (Agonoderus) comma (Fabricius, 1775)

Stenolophus (Agonoderus) lineola (Fabricius, 1775)

Stenolophus (Stenolophus) fuliginosus Dejean, 1829

**Tribe Lebiini Bonelli, 1810**

Subtribe Cymindidina Laporte, 1834

**Cymindus (Pinacodera) limbata Dejean, 1831**●

Subtribe Dromiusina Bonelli, 1810

*Dromiuspiceus* Dejean, 1831●

*Syntomusamericanus* (Dejean, 1831)

Subtribe Lebiina Bonelli, 1810

Lebia (Lebia) fuscata Dejean, 1825●

Lebia (Lebia) moesta LeConte, 1850

Lebia (Lebia) pumila Dejean, 1831

Lebia (Lebia) viridis Say, 1823

**Tribe Licinini Bonelli, 1810**

Subtribe Dicaelina Laporte, 1834

Diplocheila (Isorembus) obtusa (LeConte, 1847)

Subtribe Licinina Bonelli, 1810

Badister (Badister) neopulchellus Lindroth, 1954

Badister (Baudia) grandiceps Casey, 1920

Badister (Baudia) micans LeConte, 1844

**Tribe Platynini Bonelli, 1810**

Agonum (Agonum) muelleri (Herbst, 1784)†●

Agonum (Agonum) placidum (Say, 1823)

Agonum (Europhilus) anchomenoides Randall, 1838

Agonum (Europhilus) canadense Goulet, 1969

Agonum (Europhilus) gratiosum (Mannerheim, 1853)*

Agonum (Europhilus) lutulentum (LeConte, 1854)

Agonum (Europhilus) picicornoides Lindroth, 1966

Agonum (Europhilus) retractum LeConte, 1846

Agonum (Europhilus) sordens Kirby, 1837

Agonum (Europhilus) superioris Lindroth, 1966

Agonum (Europhilus) thoreyi Dejean, 1828*

Agonum (Olisares) affine Kirby, 1837

Agonum (Olisares) crenistriatum (LeConte, 1863)

Agonum (Olisares) cupreum Dejean, 1831

Agonum (Olisares) cupripenne (Say, 1823)

Agonum (Olisares) harrisii LeConte, 1846

Agonum (Olisares) melanarium Dejean, 1828

Agonum (Olisares) metallescens (LeConte, 1854)

Agonum (Olisares) mutatum (Gemminger & Harold, 1868)

Agonum (Olisares) octopunctatum (Fabricius, 1798)

Agonum (Olisares) propinquum (Gemminger & Harold, 1868)

Agonum (Olisares) tenue (LeConte, 1854)

Agonum (Olisares) trigeminum Lindroth, 1954

Agonum (Platynomicrus) nigriceps LeConte, 1846*

*Oxypselaphuspusillus* (LeConte, 1854)

Platynus (Batenus) mannerheimii (Dejean, 1828)*

Platynus (Platynus) decentis (Say, 1823)●

Platynus (Platynus) tenuicollis (LeConte, 1846)

**Tribe Pterostichini Bonelli, 1810**

Subtribe Pterostichina Bonelli, 1810

Poecilus (Poecilus) lucublandus (Say, 1823)

Pterostichus (Argutor) commutabilis (Motschulsky, 1866)

Pterostichus (Bothriopterus) adstrictus Eschscholtz, 1823*

*Pterostichus* (*Bothriopterus) mutus* (Say, 1823)

Pterostichus (Bothriopterus) pensylvanicus LeConte, 1873

Pterostichus (Euferonia) coracinus (Newman, 1838)●

Pterostichus (Hypherpes) tristis (Dejean, 1828)●

Pterostichus (Melanius) corvinus (Dejean, 1828)

Pterostichus (Morphnosoma) melanarius melanarius (Illiger, 1798)†●

Pterostichus (Phonias) patruelis (Dejean, 1831)

Pterostichus (Pseudomaseus) luctuosus (Dejean, 1828)

Pterostichus (Pseudomaseus) tenuis (Casey, 1924)

*Stomis* (*Stomis) pumicatus* (Panzer, 1795)†

**Tribe Sphodrini Laporte, 1834**

Subtribe Atranopsina Baehr, 1982

*Pseudamaraarenaria* (LeConte, 1847)

Subtribe Calathina Laporte, 1834

Calathus (Neocalathus) ingratus Dejean, 1828

Subtribe Sphodrina Laporte, 1834

Laemostenus (Pristonychus) terricola terricola (Herbst, 1784)†

Subtribe Synuchina Lindroth, 1956

*Synuchusimpunctatus* (Say, 1823)●

**Tribe Zabrini Bonelli, 1810**

Subtribe Amarina C.C.A. Zimmermann, 1832

Amara (Amara) aenea (DeGeer, 1774)†

Amara (Amara) communis (Panzer, 1797)†

Amara (Amara) cupreolata Putzeys, 1866

Amara (Amara) familiaris (Duftschmid, 1812)†●

Amara (Amara) littoralis Dejean, 1828*

Amara (Amara) lunicollis Schiodte, 1837*

Amara (Amara) otiosa Casey, 1918

Amara (Amara) ovata (Fabricius, 1792)†

Amara (Amarocelia) laevipennis Kirby, 1837

*Amara* (*Amarocelia) patruelis* Dejean, 1831*

Amara (Bradytus) apricaria (Paykull, 1790)†

Amara (Bradytus) avida (Say, 1823)

Amara (Bradytus) fulva (O.F. Müller, 1776)†

Amara (Bradytus) latior (Kirby, 1837)

Amara (Celia) bifrons (Gyllenhal, 1810)†

Amara (Celia) sinuosa (Casey, 1918)

Amara (Curtonotus) aulica (Panzer, 1796)†

Amara (Paracelia) quenseli quenseli (Schönherr, 1806)*

Amara (Percosia) obesa (Say, 1823)

**Family HALIPLIDAE Aubé, 1836**

(Crawling water beetles)

Haliplus (Haliplus) immaculicollis Harris, 1828

Haliplus (Haliplus) longulus LeConte, 1850

Haliplus (Liaphlus) canadensis Wallis, 1933

Haliplus (Liaphlus) cribrarius LeConte, 1850

Haliplus (Liaphlus) connexus Matheson, 1912

Peltodytes (Neopeltodytes) edentulus (LeConte, 1863)

Peltodytes (Neopeltodytes) tortulosus Roberts, 1913

**Family DYTISCIDAE Leach, 1815**

**Subfamily COPELATINAE Branden, 1885**

*Copelatusglyphicus* (Say, 1823)

**Subfamily LACCOPHILINAE Gistel, 1848**

**Tribe Laccophilini Gistel, 1848**

*Laccophilusmaculosusmaculosus* Say, 1823

**Subfamily HYDROPORINAE Aubé, 1836**

**Tribe Bidessini Sharp, 1880**

*Liodessusaffinis* (Say, 1823)

**Tribe Hydroporini Aubé, 1836**

*Boreonectesgriseostriatus* (DeGeer, 1774)

*Hydrocoluspaugus* (Fall, 1923)

*Hydrocolusstagnalis* (Gemminger & Harold, 1868)

*Hydroporusdentellus* Fall, 1917

*Hydroporusfuscipennis* Schaum, 1868*

*Hydroporusgossei* Larson & Roughly, 2000

*Hydroporusniger* Say, 1823

*Hydroporusnotabilis* LeConte, 1850*

*Hydroporusobscurus* Sturm, 1835*

*Hydroporussignatussignatus* Mannerheim, 1853

*Hydroporusstriola* (Gyllenhal, 1826)*

*Hydroporustenebrosus* LeConte, 1850

*Hydroporustristis* (Paykull, 1798)*

*Nebrioporusrotundatus* (LeConte, 1863)

*Neoporuscarolinus* (Fall, 1917)

*Neoporusclypealis* (Sharp, 1882)

*Neoporusdimidiatus* (Gemminger & Harold, 1868)

*Neoporussulcipennis* (Fall, 1917)

*Neoporusundulatus* (Say, 1823)

*Sanfilippodytespseudovilis* (Young, 1953)

**Tribe Hygrotini Portevin, 1929**

*Coelambuscompar* Fall, 1919

*Coelambusimpressopunctatus* (Schaller, 1783)*

*Coelambuslaccophilinus* (LeConte, 1878)

*Coelambuspicatus* (Kirby, 1837)

*Coelambusturbidus* (LeConte, 1855)

*Hygrotussayi* Balfour-Browne, 1944

**Tribe Hyphydrini Gistel, 1848**

*Desmopachriaconvexa* (Aubé, 1838)

**Tribe Laccornini Walfe & Roughly, 1990**

*Laccornislatens* (Fall, 1937)

**Subfamily AGABINAE C.G. Thomson, 1867**

Agabus (Acatodes) anthracinus Mannerheim, 1852

Agabus (Acatodes) discolor (Harris, 1828)*

Agabus (Acatodes) phaeopterus (Kirby, 1837)

Agabus (Acatodes) subfuscatus Sharp, 1882

Agabus (Agabus) bifarius (Kirby, 1837)*

Agabus (Agabus) punctulatus Aubé, 1838

Agabus (Gaurodytes) ambiguus (Say, 1823)

Agabus (Gaurodytes) e*rytropterus* (Say, 1823)

Agabus (Gaurodytes) semipunctatus (Kirby, 1837)

*Ilybiosomaseriatum* (Say, 1823)

*Ilybiusangustior* (Gyllenhal, 1808)*

*Ilybiusbiguttulus* (Germar, 1824)

*Ilybiusdiscedens* Sharp, 1882*

*Ilybiuserichsoni* (Gemminger & Harold, 1868)*

*Ilybiuslarsoni* (Fery & Nilsson, 1993)

*Ilybiuspleuriticus* LeConte, 1850

**Subfamily COPTOTOMINAE Branden, 1885**

*Coptotomuslonguluslenticus* Hilsenhoff, 1980

**Subfamily COLYMBETINAE Erichson, 1837**

**Tribe Colymbetini Erichson, 1837**

*Colymbetuspaykulli* Erichson, 1837*

*Colymbetussculptilis* Harris, 1829

Rhantus (Nartus) sinuatus (LeConte, 1862)

Rhantus (Rhantus) binotatus (Harris, 1828)

Rhantus (Rhantus) consimilis Motschulsky, 1859)

Rhantus (Rhantus) suturellus (Harris, 1828)*

Rhantus (Rhantus) wallisi Hatch, 1953

**Subfamily DYTISCINAE Leach, 1815**

**Tribe Aciliini C.G. Thomson, 1867**

Acilius (Acilius) semisulcatus Aubé, 1838

Acilius (Acilius) sylvanus Hilsenhoff, 1975

Acilius (Homoeolytrus) mediatus (Say, 1823)

*Graphoderusliberus* (Say, 1825)

*Graphoderusperplexus* Sharp, 1882*

**Tribe Dytiscini Leach, 1815**

*Dytiscusdauricus* Gebler, 1832*

*Dytiscusfasciventris* Say, 1824

*Dytiscusharrisii* Kirby, 1837

*Dytiscusverticalis* Say, 1823

**Tribe Hydaticini Sharp, 1880**

Hydaticus (Hydaticus) aruspex H. Clark, 1864*

**Suborder POLYPHAGA**

**Superfamily HYDROPHILOIDEA Latreille, 1802**

**Family HELOPHORIDAE Leach, 1815**

Helophorus (Helophorus) grandis Illiger, 1798†

Helophorus (Rhopalohelophorus) orientalis Motschulsky, 1860*●

**Family HYDROPHILIDAE Latreille, 1802**

**Subfamily HYDROPHILINAE Latreille, 1802**

**Tribe Berosini Mulsant, 1844**

Berosus (Berosus) sayi Hansen, 1999

**Tribe Laccobiini Houlbert, 1922**

*Laccobiusreflexipenis* Cheary, 1971

*Paracymussubcupreus* (Say, 1825)

**Tribe Hydrobiusini Mulsant, 1844**

*Hydrobiusfuscipes* (Linnaeus, 1758)*

*Hydrobiusmelaenus* (Germar, 1824)

**Tribe Hydrophilini Latreille, 1802**

*Hydrocharaobtusata* (Say, 1823)

*Tropisternusglaber* (Herbst, 1797)

*Tropisternusmixtus* (LeConte, 1855)

**Subfamily CHAETARTHRIINAE Bedel, 1881**

**Tribe Anacaenini Hansen, 1991**

*Anacaenalutescens* (Stephens, 1829)†

Crentis (Crentis) digesta (LeConte, 1855)

Crentis (Crentis) monticola (Horn, 1890)

**Subfamily ENOCHRINAE Short & Fikáček, 2013**

*Cymbiodytasemistriata* (C.C.A. Zimmermann, 1869)

***Cymbiodytavindicata* Fall, 1924**●

Enochrus (Lumetus) hamiltoni (Horn, 1890)

Enochrus (Methydrus) ochraceus (Melsheimer, 1844)

**Subfamily SPHAERIDIINAE Latreille, 1802**

**Tribe Megasternini Mulsant, 1844**

Cercyon (Cercyon) assecla Smetana, 1978●

Cercyon (Cercyon) haemorrhoidalis (Fabricius, 1775)†

*Cryptopleurumminutum* (Fabricius, 1775)†

**Tribe Sphaeridiini Latreille, 1802**

*Sphaeridiumbipustulatum* Fabricius, 1781†

*Sphaeridiumlunatum* Fabricius, 1792†

**Family HISTERIDAE Gyllenhal, 1808**

**Subfamily ABRAEINAE MacLeay, 1819**

**Tribe Acritini Wenzel, 1944**

*Aeletespolitus* (LeConte, 1853)

**Subfamily SAPRININAE Blanchard, 1845**

*Baeckmanniolusdimidiatipennis* (J.E. LeConte, 1824)

Euspilotus (Hesperosaprinus) assimilis (Paykull, 1811)

***Gnathoncusbarbatus* Bousquet & Laplante, 1999**●

*Hypocaccusfraternus* (Say, 1825)

**Subfamily DENDROPHILINAE Reitter, 1909**

**Tribe Paromalini Reitter, 1909**

*Carcinopspumilio* (Erichson, 1834)†

*Paromalusteres* LeConte, 1878●

**Subfamily HISTERINAE Gyllenhal, 1808**

**Tribe Histerini Gyllenhal, 1808**

*Atholusperplexus* (LeConte, 1863)

***Histercurtatus* J.E. LeConte, 1844**●

*Histerfurtivus* J.E. LeConte, 1859

Margarinotus (Paralister) faedatus (J.E. LeConte, 1845)

Margarinotus (Paralister) lecontei Wenzel, 1944

Margarinotus (Ptomister) brunneus (Fabricius, 1775)†

Margarinotus (Ptomister) immunis (Erichson, 1834)

Margarinotus (Ptomister) interruptus (Palisot de Beauvois, 1818)

*Psiloscelisplanipes* (LeConte, 1852)

**Tribe Platysomatini Bickhardt, 1914**

**Platysoma (Cylister) coarctatum J.E. LeConte, 1844**●

**Platysoma (Cylistus) deficiens (Casey, 1924)**●

**Superfamily STAPHYLINOIDEA Latreille, 1802**

**Family PTILIIDAE Erichson, 1845**

**Tribe Nanosellini Barber, 1924**

*Cylindroselloidesdybasi* Hall, 1999

**Tribe Ptenidiini Flach, 1889**

*Ptenidiumpusillum* (Gyllenhal, 1808)†

**Subfamily ACROTRICHINAE Reitter, 1909**

*Ptiliopycnamoerens* (Matthews, 1874)

**Family LEIODIDAE Fleming, 1821**

**Subfamily LEIODINAE Fleming, 1821**

**Tribe Agathidiini Westwood, 1838**

***Anisotomaglobososa* Hatch, 1929**●

**Subfamily CHOLEVINAE Kirby, 1837**

**Tribe Anemadini Hatch, 1928**

Subtribe Nemadina Jeannel, 1936

**Nemadus (Laferius) brachyderus (LeConte, 1863)**●

**Nemadus (Nemadus) horni Hatch, 1933**●

**Nemadus (Nemadus) triangulum Jeannel, 1936**●

**Tribe Cholevini Kirby, 1837**

Subtribe Catopina Chaudoir, 1845

*Sciodrepoidesterminans* (LeConte, 1850)●

*Prionochaetaopaca* (Say, 1825)

**Family SILPHIDAE Latreille, 1806**

**Subfamily SILPHINAE Latreille, 1806**

*Necrodessurinamensis* (Fabricius, 1775)

*Necrophilaamericana* (Linnaeus, 1758)●

*Oiceoptomanoveboracense* (Forster, 1771)

*Thanatophiluslapponicus* (Herbst, 1793)*

**Subfamily NICROPHORINAE Kirby, 1837**

*Nicrophorusdefodiens* Mannerheim, 1846●

*Nicrophorusinvestigator* Zetterstedt, 1824*

*Nicrophorusorbicollis* Say, 1825●

*Nicrophoruspustulatus* Herschel, 1807●

*Nicrophorussayi* Laporte, 1840●

*Nicrophorustomentosus* Weber, 1801

*Nicrophorusvespilloides* Herbst, 1783*

**Family STAPHYLINIDAE Latreille, 1802**

**Subfamily OMALIINAE MacLeay, 1825**

**Tribe Anthophagini C.G. Thomson, 1859**

*Acidotacrenata* (Fabricius, 1792)*●

***Acidotasubcarinata* Erichson, 1840**●

**Tribe Eusphalerini Hatch, 1957**

**Eusphalerum (Eusphalerum) orientale (Bernhauer, 1912)**●

**Eusphalerum (Eusphalerum) pothos (Mannerheim, 1843)**●

**Tribe Omaliini MacLeay, 1825**

***Phloeonomuslaesicollis* (Mäklin, 1852)**●

**Subfamily PROTEININAE Erichson, 1839**

**Tribe Proteinini Erichson, 1839**

*Megarthrusexcisus* LeConte, 1863

**Subfamily PSELAPHINAE Latreille, 1802**

**Supertribe BATRISITAE Reitter, 1882**

**Tribe Batrisini Reitter, 1882**

Subtribe Batrisina Reitter, 1882

**Batrisodes (Excavodes) frontalis (LeConte, 1849)**●

**Batrisodes (Excavodes) lineaticollis (Aubé, 1833)**●

**Supertribe EUPLECTITAE Streubel, 1839**

**Tribe Euplectini Streubel, 1839**

**Euplectus (Euplectus) duryi Casey, 1908**●

**Euplectus (Euplectus) elongatus Brendel, 1893**●

*Euplectus* (*Diplectellu*s) *karstenii* (Reichenbach, 1816)†

**Tribe Trichonychini Reitter, 1882**

Subtribe Bibloporina Park, 1951

***Bibloporusbicanalis* (Casey, 1884)**●

**Supertribe GONIACERITAE Reiter, 1882**

**Tribe Brachyglutini Raffray, 1904**

Subtribe Brachyglutina Raffray, 1904

*Reichenbachiaborealis* Casey, 1897

**Subfamily PHLOEOCHARINAE Erichson, 1839**

***Charhyphuspicipennis* (LeConte, 1863)**●

**Subfamily TACHYPORINAE MacLeay, 1825**

**Tribe Mycetoporini C.G. Thomson, 1859**

***Bryoporusrufescens* LeConte, 1863**●

***Carphacisnepigonensis* (Bernhauer, 1912)**●

*Ischnosomapictum* (Horn, 1877)

Lordithon (Bolitobus) fungicola Campbell, 1982

*Mycetoporuslepidus* (Gravenhorst, 1806)†

**Tribe Tachyporini MacLeay, 1825**

***Coproporusventriculus* (Say, 1832)**●

***Sepedophiluscinctulus* (Erichson, 1839)**●

***Sepedophiluslittoreus* (Linnaeus, 1758)**†●

Tachinus (Tachinus) corticinus Gravenhorst, 1802†●

Tachinus (Tachinus) limbatus Melsheimer, 1844

Tachinus (Tachinus) picipes Erichson, 1839

Tachinus (Tachinus) rufipes (Linnaeus, 1758)†

Tachyporus (Tachyporus) atriceps Stephens, 1832†

Tachyporus (Tachyporus) dispar (Paykull, 1789)†●

**Subfamily ALEOCHARINAE Fleming, 1821**

**Tribe Aleocharini Fleming, 1821**

Subtribe Aleocharina Fleming, 1821

Aleochara (Aleochara) curtula (Goeze, 1777)†

Aleochara (Coprochara) bilineata Gyllenhal, 1810†

Aleochara (Coprochara) verna Say, 1833

Aleochara (Xenochara) fumata Gravenhorst, 1802†

**Tribe Athetini Casey, 1910**

Subtribe Athetina Casey, 1910

*Amishaanalis* (Gravenhorst, 1802)†●

Atheta (Datomicra) acadiensis Klimaszewski & Majka, 2007

Atheta (Datomicra) dadopora C.G. Thomson, 1867*

**Atheta (Dimetrota) fanatica Casey, 1910**●

Atheta (Pseudota) klagesi Bernhauer, 1909

*Dinaraeaangustula* (Gyllenhal, 1810)†

*Mocytafungi* (Gravenhorst, 1806)†●

*Nehemitropialividipennis* (Mannerheim, 1830)†

***Peliopterathujae* (Klimaszewski & Webster, 2016)**●

***Atheta***

*Strigotaambigua* (Erichson, 1839)

**Tribe Boreocyphini Klimaszewski & Langor, 2011**

*Boreocyphawebsteri* Klimaszewski & Langor, 2011

**Tribe Homalotini Heer, 1839**

Subtribe Bolitocharina C.G. Thomson, 1859

Leptusa (Adoxopisalia) opaca Casey, 1894●

Leptusa (Eucryptusa) brevicollis Casey, 1894●

*Silusidamarginella* (Casey, 1894)

Subtribe Gyrophaenina Kraatz, 1856

*Eumicrotasocia* (Erichson, 1839)

Gyrophaena (Gyrophaena) gaudens Casey, 1906

Gyrophaena (Gyrophaena) vitrina Casey, 1911

Subtribe Homalotina Heer, 1839

***Cypheacurtula* (Erichson, 1837)**†●

*Agaricomorphavincenti* Klimaszewski & Webster, 2016

***Homolotaplana* (Gyllenhal, 1810)**†●

Subtribe Silusina Fenyes, 1918

*Silusaalternans* Sachse, 1852

*Silusacalifornica* Bernhauer, 1905

**Tribe Hypocyhtini Laporte, 1835**

*Oligotaparva* Kraatz, 1862†

**Tribe Lomechusini Fleming, 1821**

Subtribe Myrmedoniina C.G. Thomson, 1867

*Drusillacanaliculata* (Fabricius, 1787)†

Subtribe Oxypodina C.G. Thomson, 1859

***Hylotacryptica* Klimaszewski & Webster, 2016**●

***Hylotaochracea* Casey, 1906**●

***Mniusayukonensis* (Klimaszewski & Godin, 2012)**●

Subtribe Phloeoporina C.G. Thomson, 1859

***Phloeoporaoregona* Casey, 1906**●

**Tribe Placusini Mulsant & Rey, 1871**

***Placusatachyporoides* (Walt, 1838)**†●

**Tribe Tachyusini C.G. Thomson, 1859**

*Gnypetacaerula* (C.R. Sahlberg, 1830)*

**Subfamily PIESTINAE Erichson, 1839**

***Siagoniumpunctatum* (LeConte, 1866)**●

**Subfamily OSORIINAE Erichson, 1839**

**Tribe Thoracophorini Reitter, 1909**

Subtribe Clavilispinina Newton & Thayer, 1992

***Clavilispinusprolixus* (LeConte, 1877)**●

**Subfamily OXYTELINAE Fleming, 1821**

**Tribe Blediini Ádám, 2001**

*Blediusneglectus* Casey, 1890

*Blediusopaculus* LeConte, 1863

**Tribe Coprophilini Heer, 1839**

***Coprophilusstriatulus* (Fabricius, 1792)**†●

**Tribe Oxytelini Fleming, 1821**

*Anotylusrugosus* (Fabricius, 1775)†

*Carpelimusobesus* (Kiensenwetter, 1844)†

**Subfamily SCYDMAENINAE Leach, 1815**

**Supertribe SCYDMAENITAE Leach, 1815**

**Tribe Cyrtoscydmini Schaufuss, 1889**

*Brachycepsissubpunctata* (LeConte, 1852)●

***Parascydmuscorpusculus* (Casey, 1897)**●

**Subfamily STENINAE MacLeay, 1825**

Stenus (Hypostenus) flavicornis Erichson, 1840)

Stenus (Hypostenus) rossi Sanderson, 1958

Stenus (Stenus) clavicornis (Scopoli, 1763)†

Stenus (Stenus) colonus Erichson, 1840

Stenus (Stenus) erythropus Melsheimer, 1844

Stenus (Stenus) juno (Paykull, 1789)*

Stenus (Stenus) mammops mammops Casey, 1884*

Stenus (Stenus) schwarzi Casey, 1884

**Subfamily EUAESTHETINAE C.G. Thomson, 1859**

**Tribe Euaesthetini C.G. Thomson, 1859**

*Euaesthetuslaeviusculus* Mannerheim, 1844

**Subfamily PAEDERINAE Fleming, 1821**

**Tribe Paederini Fleming, 1821**

Subtribe Lathrobina Laporte, 1835

Lathrobium (Lathrobium) fauveli Duvivier, 1883

Lathrobium (Lathrobium) spissicorne Casey, 1905

*Tetartopeusfurvulus* Casey, 1905

*Tetartopeusniger* (LeConte, 1863)

Subtribe Medonina Casey, 1905

Sunius (Trachysectus) confluentus (Say, 1831)

**Subfamily STAPHYLININAE Latreille, 1802**

**Tribe Othiini C.G. Thomson, 1859**

*Atrecusamericanus* (Casey, 1906)●

**Tribe Staphylinini Latreille, 1802**

Subtribe Anisolinina Hayashi, 1993

***Tympanophoruspuncticollis* (Erichson, 1840)**●

Subtribe Philonthina Kirby, 1837

***Bisniusblandus* (Gravenhorst, 1806)**●

***Bisniusquediinus* (Horn, 1884)**●

*Bisniussiegwaldii* (Mannerheim, 1843)

*Cafiusaguayoi* Bierig, 1934

*Erichsoniusnanus* (Horn, 1884)

*Gabriuspicipennis* (Mäklin, 1852)

***Philonthuscaeruleipenniscaeruleipennis* (Mannerheim, 1830)**●

*Philonthuscarbonarius* (Gravenhorst, 1802)†

*Philonthuscognatus* Stephens, 1832†●

*Philonthusconcinnus* (Gravenhorst, 1802)†●

*Philonthuscouleensis* Hatch, 1857

*Philonthuscruentatus* (Gmelin, 1790)†

*Philonthusdebilis* (Gravenhorst, 1802)†

*Philonthusleechensis* Hatch, 1957

*Philonthuslindrothi* Smetana, 1965

*Philonthuspalliatus* (Gravenhorst, 1806)

*Philonthusrectangulus* Sharp, 1874†

*Philonthusumbratilis* (Gravenhorst, 1802)†

*Philonthusvarians* (Paykull, 1789)†●

*Philonthusvulgatus* Casey, 1915

Subtribe Quediina Kraatz, 1857

**Quedius (Microsaurus) bicoloris Smetana & Webster, 2011**●

**Quedius (Microsaurus) canadensis (Casey, 1915**)●

**Quedius (Microsaurus) mesomelinus mesomelinus (Marsham, 1802)**†●

Quedius (Quedionuchus) plagiatus Mannerheim, 1843*●

Quedius (Quedius) curtipennis Bernhauer, 1908†

Quedius (Quedius) molochinus (Gravenhorst, 1806)†

Subtribe Staphylinina Latreille, 1802

*Creophilusmaxillosusvillosus* (Gravenhorst, 1802)

Dinothenarus (Parabemus) badipes (LeConte, 1863

*Ontholestescingulatus* (Gravenhorst, 1802)●

*Platydracusviolaceus* (Gravenhorst, 1802)●

***Platydracusviridanus* (Horn, 1879)**●

Tasgius (Rayacheila) melanarius melanarius (Heer, 1839)†

Tasgius (Tasgius) ater (Gravenhorst, 1802)†

**Tribe Xantholinini Erichson, 1839**

*Gyrohypnusfracticornis* (O.F. Müller, 1776)†

*Leptacinusintermedius* Donisthorpe, 1936†

***Neohypnusbeckeri* Smetana, 1882**●

*Nudobiuscephalus* (Say, 1830)●

Xantholinus (Xantholinus) linearis linearis (Olivier, 1795)†

**Superfamily SCARABAEOIDEA Latreille, 1802**

**Family GEOTRUPIDAE Latreille, 1802**

**Subfamily GEOTRUPINAE Latreille, 1802**

**Tribe Geotrupini Latreille, 1802**

**Geotrupus (Anoplotrupes) balyi Jekel, 1865**●

Geotrupus (Geotrupus) stercorarius (Linnaeus, 1758)†

**Family TROGIDAE MacLeay, 1819**

**Subfamily TROGINAE MacLeay, 1819**

*Troxunistriatus* Palisot de Beauvois, 1818

*Troxvariolatus* Melsheimer, 1845●

**Family LUCANIDAE Latreille, 1804**

**Subfamily SYNDESINAE MacLeay, 1819**

***Ceruchuspiceus* (Weber, 1801)**●

**Subfamily LUCANINAE Latreille, 1804**

**Tribe Platycerinae Mulsant, 1842**

*Platycerusdepressus* LeConte, 1850

***Platycerusvirescens* (Fabricius, 1775)**●

**Family SCARABAEIDAE Latreille, 1802**

**Subfamily AEGIALIINAE Laporte, 1840**

Aegialia (Aegialia) opifex Horn, 1887

***Caeliusrufescens* (Horn, 1887)**●

**Subfamily APHODIINAE Leach, 1815**

**Tribe Aphodiini Leach, 1815**

Subtribe Aphodiina Leach, 1815

*Aphodiusfimetarius* (Linnaeus, 1758)†●

***Dialytesstriatulus* (Say, 1825)**●

*Melinopterusprodromus* (Brahm, 1790)†

*Teuchestesfossor* (Linnaeus, 1758)†

**Subfamily MELOLONTHINAE Leach, 1819**

**Tribe Dichelonychini Burmeister, 1855**

***Dichelonyxalbicollis* Burmeister, 1855**●

*Dichelonyxelongatula* (Schönherr, 1817)●

***Dichelonyxsubvittata* LeConte, 1856**●

**Tribe Diplotaxini Kirby, 1837**

*Diplotaxistristis* Kirby, 1837

**Tribe Melolonthini Leach, 1819**

Subtribe Melolonthina Leach, 1819

*Phyllophagaanxia* (LeConte, 1850)●

*Phyllophagadrakii* (Kirby, 1837)

**Tribe Sericini Kirby, 1837**

Subtribe Sericina Kirby, 1837

***Sericaatracapilla* (Kirby, 1837)**●

*Sericageorgianalecontei* Dawson, 1921●

*Sericatristis* LeConte, 1850

**Subfamily RUTELINAE MacLeay, 1819**

**Tribe Anomalini Struebel, 1839**

Subtribe Popillina Ohaus, 1918

*Popilliajaponica* Newman, 1838†

**Subfamily DYNASTINAE MacLeay, 1819**

**Tribe Pentodontini Mulsant, 1842**

*Tomarusrelictus* (Say, 1825)

**Subfamily CETONIINAE Leach, 1815**

**Tribe Trichiini Fleming, 1821**

Subtribe Osmodermatina Schenkling, 1922

***Osmodermascabra* (Palisot de Beauvois, 1805)**●

Subtribe Trichiina Fleming, 1821

*Trichiotinusassimilis* (Kirby, 1837)

**Superfamily SCIRTOIDEA Fleming, 1821**

**Family EUCINETIDAE Lacordaire, 1857**

*Eucinetushaemorrhoidalis* (Germar, 1818)†

***Eucinetusmorio* LeConte, 1853**●

**Family SCIRTIDAE Fleming, 1821**

**Subfamily SCIRTINAE Fleming, 1821**

*Contacyphoncollaris* (Guérin-Méneville, 1843)

*Contacyphonconfusus* (W.J. Brown, 1930)●

*Contayphonobscurus* (Guérin-Méneville, 1843)

***Contacyphonpadi* (Linnaeus, 1758)**†●

*Contacyphonruficollis* (Say, 1825)

*Contacyphonvariabilis* (Thunberg, 1785)*

*Prionocyphonlimbatus* LeConte, 1866

***Sacodespulchella* (Guérin-Méneville, 1843)**●

*Scirtestibialis* Guérin-Méneville, 1843

**Superfamily BUPRESTOIDEA Leach, 1815**

**Family BUPRESTIDAE Leach, 1815**

**Subfamily CHRYSOCHROINAE Laporte, 1835**

**Tribe Chrysochroini Laporte, 1835**

Subtribe Chalcophorina Lacordaire, 1857

*Chalcophoraliberata* (Germar, 1824)

**Tribe Dicercini Gistel, 1848**

Subtribe Dicercina Gistel, 1848

*Dicercadivaricata* (Say, 1823)●

*Dicercatenebrosa* (Kirby, 1837)

**Subfamily BUPRESTINAE Leach, 1815**

**Tribe Buprestini Leach, 1815**

Subtribe Buprestina Leach, 1815

Buprestis (Buprestis) maculativentris Say, 1824

**Tribe Chrysobothrini Gory & Laporte, 1836**

*Chrysobothrisscabripennis* Gory & Laporte, 1837

*Chrysobothrissexsignata* (Say, 1833)

*Chrysobothristrinervia* (Kirby, 1837)

**Tribe Melanophilini Bedel, 1921**

*Melanophilaacuminata* (DeGeer, 1774)*

*Phaenopsaeneola* (Melsheimer, 1845)

*Phaenopsfulvoguttata* (Harris, 1829)

**Subfamily AGRILINAE Laporte, 1835**

**Tribe Agrilini Laporte, 1835**

Subtribe Agrilina Laporte, 1835

*Agrilusanxius* Gory, 1841●

***Agrilusgranulatusliragus* Barter & W.J. Brown, 1950**●

***Agrilusmasculinus* Horn, 1891**●

*Agriluspensus* Horn, 1891

***Agriluspolitus* (Say, 1825)**●

**Superfamily BYRRHOIDEA Latreille, 1804**

**Family BYRRHIDAE Latreille, 1804**

**Subfamily BYRRHINAE Latreille, 1804**

**Tribe Byrrhini Latreille, 1804**

*Byrrhusamericanus* LeConte, 1850

*Cytilusalternatus* (Say, 1825)

**Tribe Simplocariini Mulsant & Rey, 1869**

*Simplocariasemistriata* (Fabricius, 1794)†●

**Subfamily SYNCALYPTINAE Mulsant & Rey, 1869**

**Tribe Syncalyptini Mulsant & Rey, 1869**

*Chaetophoraspinosa* (Rossi, 1794)†

**Family ELMIDAE Curtis, 1830**

**Subfamily ELMINAE Curtis, 1830**

**Tribe Elmini Curtis, 1830**

*Dubiraphiaminima* Hilsenhoff, 1973

*Optioservusfastiditus* (LeConte, 1850)

*Optioservusovalis* (LeConte, 1863)

*Stenelmiscrenata* (Say, 1824)

**Family HETEROCERIDAE MacLeay, 1825**

**Subfamily HETEROCERINAE MacLeay, 1825**

**Tribe Heterocerini MacLeay, 1825**

*Lanternariusbrunneus* (Melsheimer, 1844)

**Family PTILODACTYLIDAE Laporte, 1836**

**Subfamily PTILODACTYLINAE Laporte, 1836**

*Ptilodactylaserricollis* (Say, 1823)

**Superfamily ELATEROIDEA Leach, 1815**

**Family EUCNEMIDAE Eschscholtz, 1829**

**Subfamily MELASINAE Fleming, 1821**

**Tribe Dirhagini Reitter, 1911**

***Entomophthalmusrufiolus* (LeConte, 1866)**●

***Microrhaguspectinatus* LeConte, 1866**●

*Microrhagussubsinuatus* LeConte, 1852●

***Microrhagustriangularis* (Say, 1823)**●

**Tribe Epiphanini Muona, 1993**

***Epiphaniscornutus* Eschscholtz, 1829**●

*Hylisterminalis* (LeConte, 1866)●

***Dirrhagofarsusernae* Otto, Muona & McClarin, 2014**†●

**Tribe Melasini Fleming, 1821**

Subtribe Melasina Fleming, 1821

***Isorhipisobliqua* (Say, 1839)**●

***Isorhipisruficornis* (Say, 1823)**●

**Subfamily MACRAULACINAE Fleutiaux, 1923**

**Tribe Macraulacini Fleutiaux, 1923**

*Deltometopusamoenicornis* (Say, 1839)●

***Dromaeolusharringtoni* Horn, 1886**●

*Onichodoncanadensis* (W.J. Brown, 1940)●

***Onichodonorchesides* Newman, 1838**●

**Family THROSCIDAE Laporte, 1840**

*Aulonothroscusconstrictor* (Say, 1839)●

*Trixaguscarnicollis* (C. Schaeffer, 1916)●

**Family ELATERIDAE Leach, 1815**

**Subfamily AGRYPNINAE Candèze, 1857**

**Tribe Agrypnini Candèze, 1857**

*Danosomabrevicorne* (LeConte, 1853)●

***Laconauroratus* (Say, 1839)**●

**Subfamily LISSOMINAE Laporte, 1835**

*Oestodestenuicollis* (Randall, 1838)

**Subfamily PITYOBIINAE Hyslop, 1917**

*Pityobiusanguinus* LeConte, 1853

**Subfamily DENDROMETRINAE Gistel, 1848**

**Tribe Dendrometrini Gistel, 1848**

Subtribe Dendrometrina Gistel, 1848

***Athousacanthus* (Say, 1839)**●

***Athousbrightwelli* (Kirby, 1837)**●

*Athouscampyloides* Newman, 1833†

*Athouscucullatus* (Say, 1825)†

*Athousorvus* Becker, 1974

***Athousposticus* (Melsheimer, 1845)**●

*Athousrufifrons* (Randall, 1838)●

***Athousscapularis* (Say, 1839)**●

*Limoniusaeger* LeConte, 1853●

***Limoniusconfusus* LeConte, 1853**●

Subtribe Denticollina Stein & Weise, 1877

*Denticollisdenticornis* (Kirby, 1837)●

Subtribe Hemicrepidiina Champion, 1896

*Hemicrepidiusbrevicollis* (Candèze, 1863)●

*Hemicrepidiusmemnonius* (Herbst, 1806)●

**Tribe Hypnoidini Schwarz, 1906**

*Hypnoidusabbreviatus* (Say, 1823)●

**Tribe Prosternini Gistel, 1856**

*Corymbitodesdorothyae* (Knull, 1959)

*Corymbitodestarsalis* (Melsheimer, 1845)

***Hypoganussulcicollis* (Say, 1833)**●

*Liotrichusspinosus* (LeConte, 1853)●

*Nitidolimoniusresplendens* (Eschscholtz, 1829)●

***Oxygonusmontanus* C. Schaeffer, 1917**●

***Pseudanostirushamatus* (Say, 1834)**●

*Pseudanostirushieroglyphicus* (Say, 1834)●

*Pseudanostiruspropoluspropolus* (LeConte, 1853)●

*Pseudanostirustriundulatus* (Randall, 1838)●

*Selatosomusappropinquans* (Randall, 1838)●

*Selatosomuspulcher* (LeConte, 1853)●

*Setasomusnitidulus* (LeConte, 1853)

*Setasomusrufopleuralis* (Fall, 1933)

*Sylvanelatorcylindriformis* (Herbst, 1806)●

**Subfamily NEGASTRIINAE Nakane & Kishii, 1956**

**Tribe Negastriini Nakane & Kishii, 1956**

*Negastriusdelumbis* (Horn, 1891)

***Neohypdonustumescens* (LeConte, 1853)**●

**Subfamily ELATERINAE Leach, 1815**

**Tribe Agriotini Laporte, 1840**

Subtribe Agriotina Laporte, 1840

*Agriotescollaris* (LeConte, 1853)●

***Agriotesfucosus* (LeConte, 1853)**●

*Agrioteslimosus* (LeConte, 1853)●

*Agrioteslineatus* (Linnaeus, 1767)†

*Agriotesmancus* (Say, 1823)

*Agriotesobscurus* (Linnaeus, 1758)†

*Agriotessputator* (Linnaeus, 1758)†●

*Agriotesstabilis* (LeConte, 1853)●

***Dalopiuscognatus* W. J. Brown, 1934**●

***Dalopiusfuscipes* W. J. Brown, 1934**●

*Dalopiusgentilus* W. J. Brown, 1934

*Dalopiuspallidus* W. J. Brown, 1934

*Dalopiusvagus* W. J. Brown, 1934●

**Tribe Ampedini Gistel, 1848**

*Ampedusapicatus* (Say, 1834)●

*Ampeduslaurentinus* W.J. Brown, 1933

*Ampedusluctuosus* (LeConte, 1853)●

*Ampedusmixtus* (Herbst, 1806)●

***Ampedusoblessus* (Say, 1833)**●

***Ampedusprotervus* (LeConte, 1853)**●

*Ampeduspullus* Germar, 1844●

*Ampedusrubricus* (Say, 1825)●

*Ampedussanguinipennis* (Say, 1823)

*Ampedussemicinctus* (Randall, 1838)●

***Ampedusvitiosus* (LeConte, 1853)**●

**Tribe Elaterini Leach, 1815**

*Sericusincongruus* (LeConte, 1853)

**Tribe Megapenthini Gurjeva, 1973**

***Megapenthesrogersi* Horn, 1871**●

**Tribe Melanotini Candèze, 1859**

*Melanotuscastanipes* (Paykull, 1800)‡●

*Melanotusdecumanus* (Erichson, 1841)

*Melanotussimilis* (Kirby, 1837)●

**Tribe Pomachiliini Candèze, 1859**

***Idolusdebilis* (LeConte, 1884)**●

**Subfamily CARDIOPHORINAE Candèze, 1859**

*Cardiophorusconvexulus* LeConte, 1853

*Cardiophorusgagates* Erichson, 1840

**Family LYCIDAE Laporte, 1836**

**Subfamily DICTYOPTERINAE Houlbert, 1922**

**Tribe Dictyopterini Houlbert, 1922**

*Dictyopteraaurora* (Herbst, 1784)*●

**Subfamily LYCINAE Laporte, 1836**

**Tribe Calopterini Green, 1949**

Subtribe Calopterina Green, 1949

***Caeniadimidiata* (Fabricius, 1801)**●

***Leptoceletesbasalis* (LeConte, 1847)**●

**Tribe Erotini LeConte, 1881**

***Eropterusarculus* Green, 1951**●

***Eroshumeralis* (Fabricius, 1801)**●

**Erotides (Erotides) sculptilis (Say, 1835)**●

**Tribe Platerodini Kleine, 1929**

***Platerosflavoscutellatus* Blatchley, 1914**●

***Plateroslictor* (Newman, 1838)**●

**Family LAMPYRIDAE Rafinesque, 1815**

**Subfamily LAMPYRINAE Rafinesque, 1815**

**Tribe Cratomorphini Green, 1948**

*Pyractomenaangulata* (Say, 1825)●

***Pyractomenaborealis* (Randall, 1838)**●

**Tribe Lucidotini Lacordaire, 1857**

Subtribe Lucidotina Lacordaire, 1857

*Lucidotaatra* (Olivier, 1790)●

Subtribe Photinina LeConte, 1881

Ellychnia (Ellychnia) corrusca (Linnaeus, 1767)●

*Photinusobscurellus* LeConte, 1851

*Pyropygadecipiens* (Harris, 1836)

**Subfamily PHOTURINAE Lacordaire, 1857**

*Photurisfairchildi* Barber, 1951●

**Family CANTHARIDAE Imhoff, 1856**

**Subfamily CANTHARINAE Imhoff, 1856**

**Tribe Cantharini Imhoff, 1856**

*Cantharisrufa* Linnaeus, 1758†●

***Pacificanthiarotundicollis* (Say, 1825)**●

***Rhagonychafraxini* (Say, 1823)**●

***Rhagonychaimbecillis* (LeConte, 1851)**●

***Rhagonycharecta* (Melsheimer, 1846)**●

***Dichelotarsuspiniphilus* (Eschscholtz, 1830)**●

***Dichelotarsuspuberulus* (LeConte, 1850)**●

***Dichelotarsuspunctatus* (LeConte, 1850)**●

***Dichelotarsussimplex* (Couper, 1865)**●

***Podabrusdiadema* (Fabricius, 1798)**●

***Podabrusintrusus* Green, 1947**●

***Podabrusmodestus* (Say, 1823)**●

**Subfamily SILINAE Mulsant, 1862**

**Tribe Silini Mulsant, 1862**

***Silispercomis* (Say, 1835)**●

**Subfamily MALTHININAE Kiesenwetter, 1852**

**Tribe Malthodini Böving & Craighead, 1930**

***Malthodesfragilis* (LeConte, 1851)**●

***Malthodesniger* (LeConte, 1851)**●

*Malthodespumilus* (Brébisson, 1835)†●

**Superfamily DERODONTOIDEA LeConte, 1861**

**Family DERODONTIDAE LeConte, 1861**

**Subfamily LARICOBIINAE Mulsant & Rey, 1864**

***Laricobiusrubidus* LeConte, 1861**●

**Superfamily BOSTRICHOIDEA Latreille, 1802**

**Family DERMESTIDAE Latreille, 1804**

**Subfamily DERMESTINAE Latreille, 1804**

**Tribe Dermestini Latreille, 1804**

Dermestis (Dermestis) lardarius Linnaeus, 1758†

Dermestis (Dermestis) maculatus DeGeer, 1774†

**Subfamily ATTAGENINAE Laporte, 1840**

**Tribe Attagenini Laporte, 1840**

Attagenus (Attagenus) unicolor japonicus Reitter, 1877)†

**Subfamily MEGATOMINAE Leach, 1815**

**Tribe Anthrenini Gistel, 1848**

Anthrenus (Anthrenus) scrophulariae scrophulariae (Linnaeus, 1758)†

Anthrenus (Florilinus) castaneae Melsheimer, 1844

Anthrenus (Florilinus) museorum (Linnaeus, 1761)†

Anthrenus (Helocerus) fuscus Olivier, 1789†●

*Reesavespulae* (Milliron, 1939)

*Trogodermainclusum* LeConte, 1854

*Trogodermasternalesternale* Jayne, 1882

*Trogodermavariabile* Ballion, 1878†

**Family BOSTRICHIDAE Latreille, 1802**

**Subfamily DINODERINAE C.G. Thomson, 1863**

Dinoderus (Dinoderus) minutus (Fabricius, 1775)†

*Rhyzoperthadominica* (Fabricius, 1792)†

**Subfamily LYCTINAE Billberg, 1820**

**Tribe Lyctini Billberg, 1820**

*Lyctusbrunneus* (Stephens, 1830)†

*Lyctusplanicollis* LeConte, 1858

**Tribe Trogoxylini Lesne, 1921**

*Trogoxylonparallelopipedum* (Melsheimer, 1844)

**Family PTINIDAE Latreille, 1892**

**Subfamily PTININAE Latreille, 1802**

**Tribe Meziini Bellés, 1985**

*Meziumaffine* Boieldieu, 1856†

**Tribe Ptinini Latreille, 1802**

*Niptushololeucus* (Faldermann, 1835)†

Ptinus (Ptinus) fur (Linnaeus, 1758)†

Ptinus (Ptinus) villiger (Reitter, 1884)†

**Subfamily ERNOBIIBAE Pic, 1912**

*Ernobiusmollis* (Linnaeus, 1758)†

**Subfamily ANOBIINAE Fleming, 1821**

*Hadrobregmusnotatus* (Say, 1825)●

*Hemicoeluscarinatus* (Say, 1823)●

*Hemicoelusdefectus* (Fall, 1905)

*Microbregmaemarginatum* (Duftschmid, 1825)†●

***Oligomerusobtusus* LeConte, 1865**●

*Priobiumsericeum* (Say, 1825)●

*Stegobiumpaniceum* (Linnaeus, 1758)†

**Subfamily PTILININAE Shuckard, 1839**

***Ptilinuslobatus* Casey, 1898**●

***Ptilinuspruinosus* Casey, 1898**●

***Ptilinusruficornis* Say, 1823**●

**Subfamily XYLETININAE Gistel, 1848**

**Tribe Lasiodermini Böving, 1927**

*Lasiodermaserricorne* (Fabricius, 1792)†

*Euvrillettapeltata* (Harris, 1836)

**Tribe Xyletinini Gistel, 1848**

***Xyletinuslugubris* LeConte, 1878**●

**Subfamily DORCATOMINAE C.G. Thomson, 1859**

*Caenocaraoculatum* (Say, 1824)

*Dorcatomapallicornis* LeConte, 1874●

***Sculptothecapuberula* (LeConte, 1865)**●

**Superfamily LYMEXYLOIDEA Fleming, 1821**

**Family LYMEXYLIDAE Fleming, 1821**

**Subfamily HYLECOETINAE Germar, 1818**

***Elateroideslugubris* (Say, 1835)**●

**Superfamily CLEROIDEA Latreille, 1802**

**Family TROGOSSITIDAE Latreille, 1802**

**Subfamily PELTINAE Latreille, 1806**

**Tribe Thymalini Léveillé, 1888**

*Thymalusmarginicollis* Chevrolat, 1842●

**Subfamily TROGOSSITINAE Latreille, 1802**

**Tribe Calityini Reitter, 1922**

*Calitysscabra* (Thunberg, 1784)*●

**Tribe Trogossitini Latreille, 1802**

***Tenebriodescorticalis* (Melsheimer, 1844)**●

*Tenebriodesmauritanicus* (Linnaeus, 1758)‡

**Family CLERIDAE Latreille, 1802**

**Subfamily TILLINAE Fischer von Waldheim, 1813**

***Cymatoderabicolor* (Say, 1825)**●

**Subfamily HYDNOCERINAE Spinola, 1844**

**Tribe Hydnocerini Spinola, 1844**

*Phyllobaenushumeralis* (Say, 1823)

*Phyllobaenuslecontei* (Wolcott, 1912)

*Phyllobaenuspallipennis* (Say, 1825)

*Phyllobaenusverticalis* (Say, 1835)●

**Subfamily CLERINAE Latreille, 1802**

***Enoclerusnigripesrufiventris* (Spinola, 1844)**●

***Thanasimusdubius* (Fabricius, 1777)**●

***Thanasimusundatulusnubilus* (Klug, 1842)**●

***Thanasimusundatulusundatulus* (Say, 1835)**●

**Subfamily KORYNETINAE Laporte, 1836**

*Madonielladislocata* (Say, 1825)●

*Necrobiarufipes* (DeGeer, 1775)†

**Subfamily THANEROCLERINAE Chapin, 1924**

**Tribe Thaneroclerini Chapin, 1924**

*Zenodosussanguineus* (Say, 1835)●

**Family MELYRIDAE Leach, 1815**

**Subfamily MALACHIINAE Fleming, 1821**

**Tribe Malachiini Fleming, 1821**

Malachius (Malachius) aeneus (Linnaeus, 1761)†

***Nodopusflavilabris* (Say, 1825)**●

**Superfamily CUCUJOIDEA Latreille, 1802**

**Family BYTURIDAE Gistel, 1848**

**Subfamily BYTURINAE Gistel, 1848**

*Byturusunicolor* Say, 1823●

**Family SPHINDIDAE Jacquelin du Val, 1860**

**Subfamily ODONTOSPHINDINAE Sen Gupta & Crowson, 1979**

***Odontosphindusdenticollis* LeConte, 1878**●

**Subfamily SPHINDINAE Jacquelin du Val, 1860**

***Sphindusamericanus* LeConte, 1866**●

***Sphindustrinifer* Casey, 1898**●

**Family EROTYLIDAE Latreille, 1802**

**Subfamily EROTYLINAE Latreille, 1802**

**Tribe Tritomini Curtis, 1834**

*Triplaxdissimulator* (Crotch, 1873)●

***Triplaxfrosti* Casey, 1924**●

*Triplaxthoracica* Say, 1825●

***Tritomapulchra* Say, 1826**●

**Family MONOTOMIDAE Laporte, 1840**

**Subfamily RHIZOPHAGINAE Redtenbacher, 1845**

**Rhizophagus (Anomophagus) brunneus brunneus Horn, 1879**●

**Rhizophagus (Rhizophagus) dimidiatus Mannerheim, 1843**●

**Rhizophagus (Rhizophagus) remotus LeConte, 1866**●

**Subfamily MONOTOMINAE Laporte, 1840**

**Tribe Monotomini Laporte, 1840**

*Monotomapicipes* Herbst, 1793†●

*Monotomaproducta* LeConte, 1855

**Family CRYPTOPHAGIDAE Kirby, 1826**

**Subfamily CRYPTOPHAGINAE Kirby, 1826**

**Tribe Cryptophagini Kirby, 1826**

*Antherophagusochraceus* Melsheimer, 1844●

*Cryptophagusfallax* Balfour-Browne, 1953†

*Cryptophagusjakowlewi* Reitter, 1888*

*Henotideruscentromaculatus* Reitter, 1877*●

*Telmatophilustyphae* (Fallén, 1802)†

**Subfamily ATOMARIINAE LeConte, 1861**

**Tribe Atomariini LeConte, 1861**

Atomaria (Anchicera) ephippiata C.C.A. Zimmermann, 1869●

Atomaria (Anchicera) fuscata Schönherr, 1808†●

**Atomaria (Anchicera) lewisi Reitter, 1877**†●

Atomaria (Atomaria) affinis R.F. Sahlberg, 1834*●

**Atomaria (Atomaria) alpina Heer, 1841**†●

**Atomaria (Atomaria) constricta (Casey, 1900)**●

Atomaria (Atomaria) nigrirostris Stephens, 1830*●

**Atomaria (Atomaria) pinicola Pelletier, 2019**●

**Family SILVANIDAE Kirby, 1837**

**Subfamily Brontinae Blanchard, 1845**

**Tribe Brontini Blanchard, 1845**

***Dendrophaguscygnaei* Mannerheim, 1846**●

**Subfamily SILVANINAE Kirby, 1837**

*Ahasverusadvena* (Waltl, 1834)†

*Oryzaephilusmercator* (Fauvel, 1889)†

***Silvanusbidentatus* (Fabricius, 1792)**†●

**Family CUCUJIDAE Latreille, 1802**

***Cucujusclavipesclavipes* Fabricius, 1777**●

**Family PHALACRIDAE Leach, 1815**

**Subfamily PHALACRINAE Leach, 1815**

*Olibrussemistriatus* LeConte, 1856●

*Stilbusapicalis* (Melsheimer, 1844)

**Family LAEMOPHLOEIDAE Ganglbauer, 1899**

***Charaphloeusconvexulus* (LeConte, 1879)**●

*Cryptolestesferrugineus* (Stephens, 1832)†●

***Cryptolestesturcicus* (Grouvelle, 1876)**†●

*Laemophloeusbiguttatus* (Say, 1825)●

***Laemophloeusfasciatus* Melsheimer, 1844**●

**Family KATERETIDAE Kirby, 1837**

*Brachypteroluspulicarius* (Linnaeus, 1758)†

***Brachypterusurticae* (Fabricius, 1792)**†●

***Heterhelussericanssericans* (LeConte, 1859)**●

**Family NITIDULIDAE Latreille, 1802**

**Subfamily EPURAEINAE Kirejtshuk, 1986**

**Tribe Epuraeini Kirejtshuk, 1986**

Epuraea (Epuraea) aestiva (Linnaeus, 1758)†●

Epuraea (Epuraea) flavomaculata Mäklin, 1853●

Epuraea (Epuraea) linearis Mäklin, 1853*●

**Epuraea (Epuraea) pallescens labilis Erichson, 1843**●

**Epuraea (Epuraea) planulata Erichson, 1843**●

Epuraea (Epuraea) rufomarginata (Stephens, 1830)*●

**Epuraea (Epuraea) truncatella (Mannerheim, 1846)**●

***Epuraeaavara* (Randall, 1838)**●

**Subfamily CARPOPHILINAE Erichson, 1842**

Carpophilus (Ecnomorphus) brachypterus (Say, 1825)●

Carpophilus (Semocarpolus) marginellus Motschulsky, 1858†

**Subfamily MELIGETHINAE C.G. Thomson, 1859**

*Brassicogethessimplipes* (Easton, 1947)●

*Brassicogethesviridescens* (Fabricius, 1787)†●

*Fabogethesnigrescens* (Stephens, 1830)*●

**Subfamily NITIDULINAE Latreille, 1802**

**Tribe Cychramini Gistel, 1848**

***Cychramusadustus* Erichson, 1843**●

**Tribe Nitidulini Latreille, 1802**

***Omositanearctica* Kirejtshuk, 1987**●

**Subfamily CILLAEINAE Kirejtshuk & Audisio, 1986**

***Colopterustruncatus* (Randall, 1838)**●

*Conotelusobscurus* Erichson, 1843

**Subfamily CRYPTARCHINAE C.G. Thomson, 1859**

**Tribe Cryptarchini C.G. Thomson, 1859**

Cryptarcha (Cryptarcha) ampla Erichson, 1843●

**Glischrochilus (Glischrochilus) moratus W.J. Brown, 1932**●

**Glischrochilus (Glischrochilus) vittatus (Say, 1835**)●

Glischrochilus (Librodor) fasciatus (Olivier, 1790)●

Glischrochilus (Librodor) quadrisignatus (Say, 1835)●

Glischrochilus (Librodor) sanguinolentus sanguinolentus (Olivier, 1790)●

Glischrochilus (Librodor) siepmanni W.J. Brown, 1932●

***Pityophagusferrugineus* (Linnaeus, 1760)**†●

**Family CERYLONIDAE Billberg, 1820**

**Subfamily CERYLONINAE Billberg, 1820**

*Ceryloncastaneum* Say, 1827●

***Cerylonunicolor* (Ziegler, 1845)**●

**Family ENDOMYCHIDAE Leach, 1815**

**Subfamily ANAMORPHINAE Strohecker, 1953**

***Symbiotesduryi* Blatchley, 1910**●

**Subfamily LEIESTINAE C.G. Thomson, 1863**

*Phymaphorapulchella* Newman, 1838

**Subfamily MYCETAEINAE Jacquelin du Val, 1857**

*Mycetaeasubterranea* (Fabricius, 1801)†●

**Subfamily ENDOMYCHINAE Leach, 1815**

***Endomychusbiguttatus* Say, 1824**●

**Subfamily LYCOPERDININAE Bromhead, 1838**

***Mycetinaperpulchra* (Newman, 1838)**●

**Family COCCINELLIDAE Latreille, 1807**

**Subfamily MICROWEISEINAE Leng, 920**

**Tribe Microweiseini Leng, 1920**

*Coccidophilusmarginatus* (LeConte, 1878)

**Subfamily COCCINELLINAE Latreille, 1807**

**Tribe Brachiacanthini Mulsant, 1850**

*Brachiacanthadecempustulata* (Melsheimer, 1847)

**Tribe Chilocorini Mulsant, 1846**

***Chilocorusstigma* (Say, 1835)**●

**Tribe Coccinellini Latreille, 1807**

*Adaliabipunctata* (Linnaeus, 1758)‡

*Anatismali* (Say, 1824)●

*Anisostictabitriangularis* (Say, 1824)

*Calviaquatuordecimguttata* (Linnaeus, 1758)*

*Coccinellaseptempunctata* Linnaeus, 1758†●

*Coccinellatransversoguttatarichardsoni* W. Brown, 1962

*Coccinellatrifasciataperplexa* Mulsant, 1850

*Coccinellaundecimpunctataundecimpunctata* Linnaeus, 1758†

*Harmoniaaxyridis* (Pallas, 1773)†●

*Hippodamiaquinquesignataquinquesignata* (Kirby, 1837)

*Hippodamiatredecimpunctatatibialis* (Say, 1824)

*Hippodamiavariegata* (Goeze, 1777)†

*Mulsantinahudsonica* (Casey, 1899)●

*Myziapullata* (Say, 1826)

*Propylaeaquatuordecimpunctata* (Linnaeus, 1758)†●

*Psylloboravigintimaculata* (Say, 1824)●

**Tribe Hyperaspidini Mulsant, 1846**

***Hyperaspisbinotata* (Say, 1826)**●

*Hyperaspisoctavia* Casey, 1908

*Hyperaspisundulata* (Say, 1824)

**Tribe Scymnini Mulsant, 1846**

**Scymnus (Pullus) puncticollis LeConte, 1852**●

Scymnus (Pullus) tenebrosus Mulsant, 1850

**Family CORYLOPHIDAE LeConte, 1852**

**Subfamily CORYLOPHINAE LeConte, 1852**

**Tribe Orthoperini Jacquelin du Val, 1857**

*Orthoperussuturalis* LeConte, 1878

**Tribe Parmulini Poey, 1854**

***Clypastraealunata* (LeConte, 1852**)●

**Family LATRIDIIDAE Erichson, 1842**

**Subfamily LATRIDIINAE Erichson, 1842**

Cartodere (Aridius) bifasiata (Reitter, 1877)†

Cartodere (Aridius) nodifer (Westwood, 1839)†●

Cartodere (Cartodere) constricta (Gyllenhal, 1827)†

*Dienerellaargus* (Reitter, 1884)†

*Dienerellacostulata* (Reitter, 1877)†

*Dienerellafiliformis* (Gyllenhal, 1827)†

***Enicmustenuicornis* LeConte, 1878**●

*Latridiusminutus* (Linnaeus, 1767)†●

***Stephostethusbreviclavis* (Fall, 1899)**●

*Stephostethusliratus* (LeConte, 1863)●

**Subfamily CORTICARIINAE Curtis, 1829**

*Corticariaelongata* (Gyllenhal, 1827)†

*Corticariaserrata* (Paykull, 1798)†●

*Corticarinacavicollis* (Mannerheim, 1844)

*Corticarinaminuta* (Fabricius, 1792)*

*Cortinicaragibbosa* (Herbst, 1793)†

Melanophthalma (Cortilena) picta (LeConte, 1855●

Melanophthalma (Melanophthalma) americana (Melsheimer, 1844)

Melanophthalma (Melanophthalma) inermis Motschulsky, 1866

Melanophthalma (Melanophthalma) pumilla (LeConte, 1855)

Melanophthalma (Melanophthalma) villosa (C.C.A. Zimmermann, 1869)

**Superfamily TENEBRIONOIDEA Latreille, 1802**

**Family MYCETOPHAGIDAE Leach, 1815**

**Subfamily MYCETOPHAGINAE Leach, 1815**

**Tribe Mycetophagini Leach, 1815**

Litargus (Tilargus) tetraspilotus LeConte, 1856●

**Mycetophagus (Mycetophagus) punctatus Say, 1826**●

**Mycetophagus (Parilendus) quadriguttatus P.W.J. Müller, 1821**‡●

**Tribe Typhaeini C.G. Thomson, 1863**

*Typhaeastercorea* (Linnaeus, 1758)†

**Family CIIDAE Leach, 1819**

**Subfamily CIINAE Leach, 1819**

**Tribe CIINI Leach, 1819**

***Ceracisthoracicornis* (Ziegler, 1845)**●

*Cisamericanus* Mannerheim, 1852●

***Cisangustus* Hatch, 1962**●

***Ciscreberrimus* Mellié, 1849**●

***Cisfuscipes* Mellié, 1849**●

***Cishorridulus* Casey, 1898**●

*Cislevettei* (Casey, 1898)●

*Cisstriatulus* Mellié, 1849*●

*striolatus* Casey, 1898

*Cissubmicans* Abeille de Perrin, 1874*●

*pistorius* Casey, 1898

*Dolichocislaricinus* (Mellié, 1849)*●

*indistinctus* Hatch, 1962

***Hadreuleelongatula* (Gyllenhal, 1827)**†●

***Malacocisbrevicollis* (Casey, 1898)**●

***Orthocispunctatus* (Mellié, 1849)**●

***Plesiociscribrum* Casey, 1898**●

**Family TETRATOMIDAE Billberg, 1820**

**Subfamily TETRATOMINAE Billberg, 1820**

**Tetratoma (Abstrulia) canadensis Nikitsky & Chantal, 2004**●

**Tetratoma (Abstrulia) tesselata (Melsheimer, 1844)**●

**Subfamily PENTHINAE Lacordaire, 1859**

***Pentheobliquata* (Fabricius, 1801)**●

*Penthepimelia* (Fabricius, 1801)●

**Subfamily EUSTROPHINAE Gistel, 1848**

**Tribe Eustrophini Gistel, 1848**

***Eustrophustomentosus* Say, 1826**●

**Tribe Holostrophini Nikitsky, 1998**

*Holostrophusbifasciatus* (Say, 1824)

**Family MELANDRYIDAE Leach, 1815**

**Subfamily MELANDRYINAE Leach, 1815**

**Tribe Hypulini Gistel, 1848**

***Hypulussimulator* Newman, 1838**●

*Symphoraflavicollis* (Haldeman, 1848)●

**Tribe Melandryini Leach, 1815**

***Emmesaconnectens* Newman, 1838**●

*Melandryastriata* Say, 1824●

**Tribe Orchesiini Mulsant, 1856**

***Orchesiacastanea* (Melsheimer, 1846)**●

***Orchesiacultriformis* Laliberté, 1967**●

*Orchesiaovata* Laliberté, 1967●

**Tribe Serropalpini Latreille, 1829**

*Dircaealiturata* (LeConte, 1866)●

*Dolotarsuslividus* (C.R. Sahlberg, 1833)*●

***Enchodessericea* (Haldeman, 1848)**●

*Phloiotryafusca* (LeConte, 1878)●

***Scotochroaatra* LeConte, 1874**●

***Scotochroabuprestoides* (Kirby, 1837)**●

***Scotochroidesantennatus* Mank, 1839**●

***Serropalpuscoxalis* Mank, 1839**●

***Serropalpussubstriatus* Haldeman, 1848**●

*Xylitalaevigata* (Hellenius, 1786)*●

**Family MORDELLIDAE Latreille, 1802**

**Subfamily MORDELLINAE Latreille, 1802**

**Tribe Mordellini Latreille, 1802**

*Mordellamarginatamarginata* Melsheimer, 1846

*Mordellariaborealis* (LeConte, 1862)●

*Mordellariaserval* (Say, 1835)●

***Tomoxialineela* LeConte, 1862**●

**Tribe Mordellistenini Ermisch, 1941**

*Mordellinainfima* (LeConte, 1862)

*Mordellinanigricans* (Melsheimer, 1846)

*Mordellinapustulata* (Melsheimer, 1846)●

***Mordellistenaandreae* LeConte, 1862**●

*Mordellistenaaspersa* (Melsheimer, 1846)

*Mordellistenacervicalis* LeConte, 1862

***Mordellistenafrosti* Liljeblad, 1918**●

*Mordellistenafuscipennis* (Melsheimer, 1846)●

*Mordellistenalimbalis* (Melsheimer, 1846)●

*Mordellistenapicilabris* Helmuth, 1864●

*Mordellistenarubrifascia* Liljeblad, 1945

*Mordellistenasericans* Fall, 1907

***Mordellistenatosta* LeConte, 1862**●

*Mordellistenavilis* (LeConte, 1858)

***Mordellochroascapularis* (Say, 1824)**●

**Family RIPIPHORIDAE Gemminger, 1870**

**Subfamily PELECOTOMINAE Seidlitz, 1875**

***Pelecotomaflavipes* Melsheimer, 1846**●

**Family ZOPHERIDAE Solier, 1834**

**Subfamily COLYDIINAE Billberg, 1820**

**Tribe Synchitini Erichson, 1845**

***Lasconotusborealis* Horn, 1878**●

***Synchitafuliginosa* Melsheimer, 1844**●

**Subfamily ZOPHERINAE Solier, 1834**

**Tribe Phellopsini Ślipiński & Lawrence, 1999**

*Phellopsisobcordata* (Kirby, 1837)

**Family TENEBRIONIDAE Latreille, 1802**

**Subfamily LAGRIINAE Latreille, 1825**

**Tribe Goniaderini Lacordaire, 1859**

***Paratenetusexutus* Bousquet & Bouchard, 2014**●

**Tribe Lagriini Latreille, 1825**

Subtribe Lagriina Latreille, 1825

*Arthromacraaeneaaenea* (Say, 1824)●

**Subfamily TENEBRIONINAE Latreille, 1802**

**Tribe Alphitobiini Reitter, 1917**

*Alphitobiusdiaperinus* (Panzer, 1796)†

**Tribe Bolitophagini Kirby, 1837**

*Bolitophaguscorticola* Say, 1826

*Bolitotheruscornutus* (Fabricius, 1801)●

**Tribe Opatrini Brullé, 1832**

*Blapstinusmetallicus* (Fabricius, 1801)

**Tribe Tenebrionini Latreille, 1802**

***Neatustenebrioides* (Palisot de Beauvois, 1811)**●

*Tenebriomolitor* Linnaeus, 1758†

**Tribe Triboliini Gistel, 1848**

Tribolium (Tribolium) castaneum (Herbst, 1797)‡

Tribolium (Tribolium) confusum Jacquelin du Val, 1861‡

Tribolium (Tribolium) destructor Uyttenboogaart, 1933†

**Subfamily ALLECULINAE Laporte, 1840**

**Tribe Alleculini Laporte, 1840**

Subtribe Alleculina Laporte, 1840

*Hymenorusmolestus* Fall, 1931●

*Hymenorusniger* (Melsheimer, 1846)●

Subtribe Gonoderina Seidlitz, 1896

***Androchiruserythropus* (Kirby, 1837)**●

*Capnochroafuliginosa* (Melsheimer, 1846)

*Isomiraquadristriata* (Couper, 1865)●

***Mycetocharabicolor* (Couper, 1865)**●

***Mycetocharabinotata* (Say, 1824)**●

***Mycetocharafoveata* (LeConte, 1866)**●

***Mycetocharafraterna* (Say, 1824)**●

**Subfamily DIAPERINAE Latreille, 1802**

**Tribe Diaperini Latreille, 1802**

Subtribe Diaperina Latreille, 1802

*Diaperismaculata* Olivier, 1791●

*Neomidabicornis* (Fabricius, 1777)

**Tribe Hypophlaeini Billberg, 1820**

***Corticeuspraetermissus* (Fall, 1926)**●

**Tribe Scaphidemini Reitter, 1922**

*Scaphidemaaeneolum* (LeConte, 1850)●

**Subfamily STENOCHIINAE Kirby, 1837**

**Tribe Cnodalonini Oken, 1843**

*Upisceramboides* (Linnaeus, 1758)*

**Family SYNCHROIDAE Lacordaire, 1859**

*Synchroapunctata* Newman, 1838●

**Family STENOTRACHELIDAE C.G. Thomson, 1859**

**Subfamily CEPHALOINAE LeConte, 1862**

*Cephaloonlepturoides* Newman, 1838●

*Cephaloonungulare* LeConte, 1874

**Family OEDEMERIDAE Latreille, 1810**

**Subfamily CALOPODINAE Costa, 1852**

***Calopusangustus* LeConte, 1851**●

**Subfamily OEDEMERINAE Latreille, 1810**

**Tribe Asclerini Gistel, 1848**

***Asclerapuncticollis* (Say, 1824)**●

***Ascleraruficollis* (Say, 1824)**●

**Tribe Nacerdini Mulsant, 1858**

*Nacerdesmelanura* (Linnaeus, 1758)†

**Family MELOIDAE Gyllenhal, 1810**

**Subfamily MELOINAE Gyllenhal, 1810**

**Tribe Meloini Gyllenhal, 1810**

Meloe (Meloe) impressus Kirby, 1837

**Family PYTHIDAE Solier, 1834**

*Priognathusmonilicornis* (Randall, 1838)●

*Pythoniger* Kirby, 1837

**Family PYROCHROIDAE Latreille, 1806**

**Subfamily PEDILINAE Lacordaire, 1859**

*Pediluslugubris* (Say, 1826)●

**Subfamily PYROCHROINAE Latreille, 1806**

*Dendroidescanadensis* Latreille, 1810●

*Dendroidesconcolor* (Newman, 1838)●

**Family SALPINGIDAE Leach, 1815**

**Subfamily SALPINGINAE Leach, 1815**

***Rhinosimusviridiaeneus* Randall, 1838**●

**Family ANTHICIDAE Latreille, 1819**

**Subfamily ANTHICINAE Latreille, 1819**

**Tribe Anthicini Latreille, 1819**

*Amblyderusgranularis* (LeConte, 1850)

*Amblyderuspallens* (LeConte, 1850)

*Anthicusflavicans* LeConte, 1852

*Malporusformicarius* (LaFerté-Sénectère, 1847)

*Omonadusfloralis* (Linnaeus, 1758)†

*Omonadusformicarius* (Goeze, 1777)†

**Subfamily NOTOXINAE Stephens, 1829**

*Notoxusanchora* Hentz, 1827

**Family ADERIDAE Csiki, 1909**

**Tribe Aderini Csiki, 1909**

Subtribe Syzetoninina Báguena Corella, 1948

*Vanonuswickhami* Casey, 1895●

**Family SCRAPTIIDAE Gistel, 1848**

**Subfamily SCRAPTIINAE Gistel, 1848**

**Tribe Scraptiini Gistel, 1848**

*Canifapallipes* (Melsheimer, 1846)●

**Subfamily ANASPIDINAE Mulsant, 1856**

**Tribe Anaspidini Mulsant, 1856**

*Anaspisflavipennis* Haldeman, 1848●

*Anaspisrufa* Say, 1826●

**Superfamily CHRYSOMELOIDEA Latreille, 1802**

**Family CERAMBYCIDAE Latreille, 1802**

**Subfamily PRIONINAE Latreille, 1802**

**Tribe Meroscelisini J. Thomson, 1860**

***Tragosomaharrisii* LeConte, 1851**●

**Subfamily LEPTURINAE Latreille, 1802**

**Tribe Lepturini Latreille, 1802**

*Analepturalineola* (Say, 1824)●

*Bellamirascalaris* (Say, 1826)●

*Brachylepturachamplaini* Casey, 1913

*Grammopterasubargentata* (Kirby, 1837)●

***Judoliamontivagansmontivagans* (Couper, 1864)**●

Lepturobosca (Cosmosalia) chrysocoma (Kirby, 1837)

*Pygolepturanigrellanigrella* (Say, 1826)

*Stictolepturacanadensiscanadensis* (Olivier, 1795)●

*Strangaleptaabbreviata* (Germar, 1824)●

*Strophionanitens* (Forster, 1771)

*Trachysidaasperabrevifrons* (Howden, 1959)

*Trachysidamutabilis* (Newman, 1841)●

*Trigonarthrissubpubescens* (Kirby, 1837)

*Typocerusvelutinusvelutinus* (Olivier, 1795)

**Tribe Oxymirini Danilevsky, 1997**

*Anthophylaxattenuatus* (Haldeman, 1847)●

***Anthophylaxcyaneus* (Haldeman, 1847)**●

*Anthophylaxviridis* LeConte, 1850●

**Tribe Rhagiini Kirby, 1837**

*Acmaeopsproteusproteus* (Kirby, 1837)●

*Evodinusmonticolamonticola* (Randall, 1838)●

Pidonia (Pidonia) ruficollis (Say, 1824)●

**Pidonia (Pidonia) vibex (Newman, 1841)**●

*Rhagiuminquisitor* (Linnaeus, 1758)*●

**Subfamily SPONDYLIDINAE Audinet-Serville, 1832**

**Tribe Asemini J. Thomson, 1860**

*Arhopalusfoveicollis* (Haldeman, 1847)

*Asemumstriatum* (Linnaeus, 1758)*●

*Tetropiumcinnamopterum* Kirby, 1837●

***Tetropiumschwarzianum* Casey, 1891**●

**Subfamily CERAMBYCINAE Latreille, 1802**

**Tribe Anaglyptini Lacordaire, 1868**

*Microclytuscompressicollis* (Laporte & Gory, 1835)●

**Tribe Callidiini Kirby, 1837**

*Callidiumviolaceum* (Linnaeus, 1758)†

*Phymatodesdimidiatus* (Kirby, 1837)●

***Phymatodesmaculicollis* LeConte, 1878**●

***Phymatodeshuetheri* Wappes & Santos-Silva, 2019**●

*Pronoceracollariscollaris* (Kirby, 1837)

*Ropalopussanguinicollis* (Horn, 1860)

**Tribe Clytini Mulsant, 1839**

***Clytusmarginicollis* Laporte & Gory, 1835**●

*Clytusruricola* (Olivier, 1795)●

*Glycobiusspeciosus* (Say, 1828)●

*Megacyllenerobiniae* (Forster, 1771)

***Neoclytusacuminatusacuminatus* (Fabricius, 1775)**●

*Neoclytusleucozonusleucozonus* (Laporte & Gory, 1835)●

***Sarosesthesfulminans* (Fabricius, 1775)**●

*Xylotrechuscolonus* (Fabricius, 1775)●

*Xylotrechusinteger* (Haldeman, 1847)●

***Xylotrechusquadrimaculatus* (Haldeman, 1847)**●

*Xylotrechussagittatussagittatus* (Germar, 1821)●

*Xylotrechusundulatus* (Say, 1824)●

**Tribe Molorchini Gistel, 1848**

***Molorchusbimaculatusbimaculatus* Say, 1824**●

**Subfamily LAMIINAE Latreille, 1825**

**Tribe Acanthocinini Blanchard, 1845**

*Acanthocinuspusillus* (Kirby, 1837)●

***Astyleiopusvariegatus* (Haldeman, 1847)**●

***Astylopsismacula* (Say, 1826)**●

***Astylopsissexguttata* (Say, 1826)**●

***Graphisurusfasciatus* (DeGeer, 1775)**●

*Hyperplatysaspersa* (Say, 1824)

***Hyperplatysmaculata* Haldeman, 1847**●

***Sternidiusrusticus* (LeConte, 1852**)●

***Urgleptessignatus* (LeConte, 1852)**●

**Tribe Desmiphorini J. Thomson, 1860**

***Psenocerussupernotatus* (Say, 1823)**●

**Tribe Monochamini Gistel, 1848**

***Microgoesoculatus* (LeConte, 1862)**●

***Monochamusmarmorator* Kirby, 1837**●

*Monochamusnotatus* (Drury, 1773)●

*Monochamusscutellatus* (Say, 1824)●

**Tribe Pogonocherini Mulsant, 1839**

***Pogonocheruspencillatus* LeConte, 1850**●

**Tribe Saperdini Mulsant, 1839**

*Saperdacalcarata* Say, 1824

*Saperdainornata* Say, 1824

*Saperdalateralis* Fabricius, 1775

*Saperdaobliqua* Say, 1826

**Tribe Tetropini Portevin, 1927**

***Tetropspraeusta* (Linnaeus, 1758)**†●

**Family MEGALOPODIDAE Latreille, 1802**

**Subfamily ZEUGOPHORINAE Böving & Craighead, 1931**

Zeugophora (Zeugophora) abnormis (LeConte, 1850)●

**Zeugophora (Zeugophora) puberula Crotch, 1873**●

**Zeugophora (Zeugophora) scutellaris Suffrian, 1840**†●

**Zeugophora (Zeugophora) varians Crotch, 1873**●

**Family ORSODACNIDAE C.G. Thomson, 1859**

**Subfamily ORSODACNINAE C.G. Thomson, 1859**

*Orsodacneatra* (Ahrens, 1810)●

**Family CHRYSOMELIDAE Latreille, 1802**

**Subfamily BRUCHINAE Latreille, 1802**

**Tribe Bruchini Latreille, 1802**

Subtribe Acanthoscelidina Bridwell, 1946

*Acanthoscelidesobtectus* (Say, 1831)†

*Callosobruchusmaculatus* (Fabricius, 1775)

Subtribe Bruchina Latreille, 1802

*Bruchuspisorum* (Linnaeus, 1758)†

Subtribe Megacerina Bridwell, 1946

Megacerus (Megacerus) discoidus (Say, 1824)

**Subfamily DONACIINAE Kirby, 1837**

**Tribe Donaciini Kirby, 1837**

*Donacia (Donaciomima) caerulea* Olivier, 1795

*Donacia (Donaciomima) distincta* LeConte, 1851

*Donacia (Donaciomima) fulgens* LeConte, 1851

*Donacia (Donaciomima) hirticollis* Kirby, 1837

**Tribe Haemoniini Chen, 1941**

*Neohaemonianigricornis* (Kirby, 1837)

**Tribe Plateumarini Böving, 1922**

*Plateumarisnitida* (Germar, 1811)

*Plateumarispusilla* (Say, 1826)

*Plateumarisrufa* (Say, 1826)

*Plateumarisshoemakeri* (C. Schaeffer, 1925)

**Subfamily CRIOCERINAE Latreille, 1804**

**Tribe Criocerini Latreille, 1804**

*Criocerisasparagi* (Linnaeus, 1758)†

*Criocerisduodecimpunctata* (Linnaeus, 1758)†

*Lilioceruslilii* (Scopoli, 1763)†

**Tribe Lemini Gyllenhal, 1813**

Oulema (Oulema) melanopus (Linnaeus, 1758)†

**Subfamily CASSIDINAE Gyllenhal, 1813**

**Tribe Cassidini Gyllenhal, 1813**

Cassida (Cassida) rubiginosa O.F. Müller, 1776†

Cassida (Mionycha) flaveola Thunberg, 1794†●

Charidotella (Charidotella) sexpunctata bicolor (Fabricius, 1798)

*Deloyalaguttata* (Olivier, 1790)●

**Tribe Chalepini Weise, 1910**

*Sumitrosisinaequalis* (Weber, 1801)●

*Sumitrosisrosea* (Weber, 1801)

**Tribe Uroplatini Weise, 1910**

*Microrhopalaexcavataexcavata* (Olivier, 1808)

**Subfamily CHRYSOMELINAE Latreille, 1802**

**Tribe Chrysomelini Latreille, 1802**

Calligrapha (Bidensomela) californica coreopsivora W.J. Brown, 1945

Calligrapha (Calligrapha) alni C. Schaeffer, 1928

Calligrapha (Calligrapha) alnicola W.J. Brown, 1945

Calligrapha (Calligrapha) ignota W.J. Brown, 1945●

**Calligrapha (Calligrapha) knabi W.J. Brown, 1940**●

Calligrapha (Calligrapha) multipunctata (Say, 1824)

Calligrapha (Calligrapha) rowena Knab, 1909●

Chrysolina (Hypericia) hyperici hyperici (Forster, 1771)†

Chrysomela (Macrolina) mainensis mainensis Bechyné, 1954

Gastrophysa (Gastrophysa) polygoni (Linnaeus, 1758)†

*Leptinotarsadecemlineata* (Say, 1824)

Phaedon (Phaedon) laevigatus (Duftschmid, 1825)†

Phratora (Phratora) purpurea purpurea W.J. Brown, 1951●

Prasocuris (Hydrothassa) vittata (Olivier, 1807)

**Subfamily GALERUCINAE Latreille, 1802**

**Tribe Alticini Newman, 1834**

Altica (Altica) ambiens alni Harris, 1869●

Altica (Altica) corni Woods, 1918

Altica (Altica) prasina populi W.J. Brown, 1938

***Chaetocnemahortensis* (Geoffroy, 1785)**†●

*Chaetocnemaconcinna* (Marsham, 1802)†●

*Chaetocnemaminuta* Melsheimer, 1847

*Crepidoderanana* (Say, 1824)

*Diboliaborealis* Chevrolat, 1834

*Diboliachelones* Parry, 1974

*Disonychatriangularis* (Say, 1824)

*Disonychaxanthomelas* (Dalman, 1823)

*Epitrixcucumeris* (Harris, 1851)●

*Longitarsusferrugineus* (Foudras, 1859)†

*Longitarsusganglbauri* Heikertinger, 1873†

*Longitarusjacobaeae* Waterhouse, 1858†

*Longitarusluridis* (Scopoli, 1763)†

*Longitaruspratensis* (Panzer, 1794)†

*Manturachrysanthemi* (Koch, 1803)†

*Phyllotretaarmoraciae* (Koch, 1803)†

*Phyllotretachalybeipennis* (Crotch, 1873)

*Phyllotretacruciferae* (Goeze, 1777)†●

*Phyllotretastriolata* (Fabricius, 1803)†●

*Phyllotretazimmermanni* (Crotch, 1873)*

*Psylliodesaffinis* (Paykull, 1799)†

*Psylliodesnapi* (Fabricius, 1792)†

*Systenafrontalis* (Fabricius, 1801)

**Tribe Galerucini Latreille, 1802**

*Erynephalamaritima* (LeConte, 1865)

Galerucella (Galerucella) nymphaeae (Linnaeus, 1758)*

*Neogalerucellacalmariensis* (Linnaeus, 1767)†

*Neogalerucellapusilla* (Duftschmid, 1825)†

*Ophraellaconferta* (LeConte, 1865)

*Pyrrhaltaviburni* (Paykull, 1799)†●

*Tricholochmaeacavicollis* (LeConte, 1865)

*Tricholochmaeakalmiae* (Fall, 1924)

*Tricholochmaeatuberculata* (Say, 1824)

*Tricholochmaeavaccinii* (Fall, 1924)

*Trirhabdaborealis* Blake, 1931

*Trirhabdacanadensis* (Kirby, 1837)

*Xanthogalerucaluteola* (O.F. Müller, 1766)†

**Tribe Luperini Gistel, 1848**

*Acalymmavittatum* (Fabricius, 1775)

*Diabroticabarberi* R.F. Smith & Lawrence, 1967

**Subfamily CRYPTOCEPHALINAE Gyllenhal, 1813**

**Tribe Cryptocephalini Gyllenhal, 1813**

Subtribe Cryptocephalina Gyllenhal, 1813

*Cryptocephalusgibbicollisgibbicollis* Haldeman, 1849

*Cryptocephalusnotatus* Fabricius, 1787

*Diachusauratus* (Fabricius, 1801)●

*Diachuscatarius* (Suffrian, 1852)●

*Triachusvacuus* LeConte, 1880

Subtribe Monachulina Leng, 1920

*Lexiphanessaponatus* (Fabricius, 1801)

Subtribe Pachybrachina Chapuis, 1874

**Pachybrachis (Pachybrachis) obsoletus Suffrian, 1852**●

Pachybrachis (Pachybrachis) peccans Suffrian, 1852●

**Tribe Fulcidacini Jakobson, 1924**

*Exemacanadensis* Pierce, 1940

*Neochlamisuscribripennis* (LeConte, 1878)

**Subfamily EUMOLPINAE Hope, 1840**

**Tribe Bromiini Baly, 1865**

*Bromiusobscurus* (Linnaeus, 1758)*●

*Graphopspubescens* (Melsheimer, 1847)

*Xanthoniadecemnotata* (Say, 1824)●

*Xanthoniaserrata* Staines & Weissman, 2001)

**Tribe Eumolpini Hope, 1840**

*Rhabdopteruspraetextus* (Say, 1824)

**Tribe Typophorini Baly, 1865**

*Pariafragariaefragariae* Wilcox, 1954

*Pariathoracica* (Melsheimer, 1847)

**Subfamily SYNETINAE LeConte & Horn, 1883**

*Synetaextorrisborealis* W. J. Brown, 1961●

*Synetaferruginea* (Germar, 1811)●

**Superfamily CURCULIONOIDEA Latreille, 1802**

**Family NEMONYCHIDAE Bedel, 1882**

**Subfamily CIMBERIDINAE Gozis, 1882**

**Tribe Cimberidini Gozis, 1882**

***Cimberiselongata* (LeConte, 1876)**●

***Cimberispallipennis* (Blatchley, 1916)**●

**Family ANTHRIBIDAE Billberg, 1820**

**Subfamily ANTHRIBINAE Billberg, 1820**

**Tribe Anthribini Billberg, 1820**

*Anthribusnebulosus* Forster, 1770†^1^●

**Tribe Trigonorhinini Valentine, 1999**

*Trigonorhinussticticus* (Boheman, 1833)

**Family ATTELABIDAE Billberg, 1820**

**Subfamily RHYNCHITINAE Gistel, 1848**

**Tribe Auletini Desbrochers des Loges, 1908**

Subtribe Auletina Desbrochers des Loges, 1908

Auletobius (Mesauletes) cassandrae (LeConte, 1876)

**Tribe Rhynchitini Gistel, 1848**

***Temnoceruscyanellus* (LeConte, 1876)**●

*Temnocerusperplexus* (Blatchley, 1916)

**Family BRENTIDAE Billberg, 1820**

**Subfamily APIONINAE Schönherr, 1823**

**Supertribe APIONITAE Schönherr, 1823**

**Tribe Apionini Schönherr, 1823**

Subtribe Aplemonina Kissinger, 1968

*Perapioncurtirostre* (Germar, 1817)†

Subtribe Synapiina Alonso-Zarazaga, 1990

Ischnoterapion (Chorapion) virens (Herbst, 1797)†

Subtribe Trichapiina Alonso-Zarazaga, 1990

***Betulapionsimilewalshii* (J.B. Smith, 1884)**●

**Apionini Incertae Sedis**

*Coelocephalapionemaciipes* (Fall, 1898)

**Family DRYOPHTHORIDAE Schönherr, 1825**

**Subfamily DRYOPHTHORINAE Schönherr, 1825**

***Dryophthorusamericanus* Bedel, 1885**●

**Subfamily RHYNCHOPHORINAE Schönherr, 1833**

**Tribe Litosomini Lacordaire, 1865**

*Sitophilusgranarius* (Linnaeus, 1758)†

*Sitophilusoryzae* (Linnaeus, 1763)†

**Tribe Sphenophorini Lacordaire, 1865**

*Sphenophoruscostipennis* Horn, 1873

*Sphenophoruszeae* Walsh, 1867

**Family BRACHYCERIDAE Billberg, 1820**

**Subfamily ERIRHININAE Schönherr, 1825**

**Tribe Erirhinini Schönherr, 1825**

*Notarispuncticollis* (LeConte, 1876)

**Family CURCULIONIDAE Latreille, 1802**

**Subfamily CURCULIONINAE Latreille, 1802**

**Tribe Anthonomini C.G. Thomson, 1859**

Anthonomus (Anthonomus) corvulus LeConte, 1876

Anthonomus (Anthonomus) lecontei Burke, 1975●

Anthonomus (Anthonomus) molochinus Dietz, 1891

Anthonomus (Anthonomus) signatus Say, 1832●

Anthonomus (Cnemocyllus) elongatus LeConte, 1876

**Anthonomus (Tachypterellus) quadrigibbus Say, 1832**●

***Pseudanthonomusrufulus* Dietz, 1891**●

***Pseudanthonomusseriesetosus* Dietz, 1891**●

*Pseudanthonomusvalidus* Dietz, 1891●

**Tribe Ellescini C.G. Thomson, 1859**

Subtribe Dorytomina Bedel, 1886

***Dorytomusparvicollis* Casey, 1892**●

**Tribe Mecinini Gistel, 1848**

*Mecinuspascuorum* (Gyllenhal, 1813)†

*Rhinusaantirrhini* (Paykull, 1800)†

*Rhinusatetra* (Fabricius, 1792)†

**Tribe Piazorhinini Lacordaire, 1863**

*Piazorhinusscutellaris* (Say, 1826)●

**Tribe Rhamphini Rafinesque, 1815**

Subtribe Rhamphina Rafinesque, 1815

*Isochnussequensi* (Stierlin, 1894)†

*Orchestesmixtus* Blatchley, 1916●

*Orchestespallicornis* Say, 1832

*Orchestestestaceus* (O.F. Müller, 1776)*●

***Tachyergesephippiatus* (Say, 1832)**●

*Tachyergesniger* (Horn, 1873)

*Tachyergessalicis* (Linnaeus, 1758)*●

**Tribe Tychiini Gistel, 1848**

Subtribe Tychiina Gistel, 1848

*Tychiusmeliloti* Stephens, 1831†

*Tychiuspicirostris* (Fabricius, 1787)†●

*Tychiusstephensi* Schönherr, 1836†

**Subfamily BARIDINAE Schönherr, 1836**

**Tribe Apostasimerini Schönherr, 1844**

Subtribe Zygobaridina Pierce, 1907

Dirabius (Dirabius) rectirostris (LeConte, 1876)

***Stethobarisovata* (LeConte, 1868)**●

**Subfamily CEUTORHYNCHINAE Gistel, 1848**

**Tribe Ceutorhynchini Gistel, 1848**

*Ceutorhynchuserysimi* (Fabricius, 1787)†●

*Ceutorhynchushamiltoni* Dietz, 1896

*Ceutorhynchusquerceti* (Gyllenhal, 1813)*

*Glocianuspunctiger* (C.R. Sahlberg, 1835)†●

**Tribe Cnemogonini Colonnelli, 1979**

*Acanthoscelidiusacephalus* (Say, 1824)

*Auleutesepilobii* (Paykull, 1800)*●

*Parauleutesnebulosus* (LeConte, 1876)

*Perigasterliturata* (Dietz, 1896)

**Tribe Phytobiini Gistel, 1848**

*Rhinoncusleucostigma* (Marsham, 1802)†

*Rhinoncuslongulus* LeConte, 1876

*Rhinoncuspericarpius* (Linnaeus, 1758)†●

*Rhinoncuspyrrhopus* Boheman 1845†

**Tribe Scleropterini Schultze, 1902**

*Prorutidosomadecipiens* (LeConte, 1876)

**Subfamily CONODERINAE Schönherr, 1833**

**Tribe Lechriopini Lacordaire, 1865**

***Acoptussuturalis* LeConte, 1876**●

*Lechriopsoculatus* (Say, 1824)

**Subfamily COSSONINAE Schönherr, 1825**

**Tribe Rhyncolini Gistel, 1848**

Subtribe Phloeophagina Voss, 1955

***Phloeophagusapionides* Horn, 1873**●

Subtribe Rhyncolina Gistel, 1848

*Carphonotustestaceus* Casey, 1892●

***Himatiumerrans* LeConte, 1876**●

*Rhyncolusbrunneus* Mannerheim, 1843

***Rhyncolusmacrops* Buchanan, 1946**●

**Subfamily CRYPTORHYNCHINAE Schönherr, 1825**

**Tribe Cryptorhynchini Schönherr, 1825**

Subtribe Cryptorhynchina Schönherr, 1825

*Cryptorhynchuslapathi* (Linnaeus, 1758)*●

**Subfamily CYCLOMINAE Schönherr, 1826**

**Tribe Listroderini LeConte, 1876**

*Listronotusalternatus* (Dietz, 1889)

*Listronotusappendiculatus* (Boheman, 1842)

*Listronotusoregonensisoregonensis* (LeConte, 1857)

*Listronotussparsus* (Say, 1832)

**Subfamily ENTIMINAE Schönherr, 1823**

**Tribe Brachyderini Schönherr, 1826**

*Strophosomamelanogrammum* (Forster, 1771)†

**Tribe Cneorhinini Lacordaire, 1863**

*Philopedonplagiatum* (Schaller, 1783)†

**Tribe Geonemini Gistel, 1848**

*Barynotusobscurus* (Fabricius, 1775)†

*Barynotusschoenherri* (Zetterstedt, 1838)†

**Tribe Hormorini Horn, 1876**

*Hormorusundulatus* (Uhler, 1856)

**Tribe Otiorhynchini Schönherr, 1826**

*Otiorhynchusligneus* (Olivier, 1807)†

*Otiorhynchusovatus* (Linnaeus, 1758)†●

*Otiorhynchussingularis* (Linnaeus, 1767)†●

*Otiorhynchussulcatus* (Fabricius, 1775)†

**Tribe Phyllobiini Schönherr, 1826**

*Phyllobiusoblongus* (Linnaeus, 1758)†●

**Tribe Polydrusini Schönherr, 1823**

*Polydrususcervinus* (Linnaeus, 1758)†●

*Polydrususformosus* (Mayer, 1779)†●

*Polydrususimpressifrons* Gyllenhal, 1834†●

**Tribe Sciaphilini Sharp, 1891**

*Barypeithespellucidus* (Boheman, 1834)†●

*Sciaphilusasperatus* (Bonsdorff, 1785)†●

**Tribe Sitonini Gistel, 1848**

*Sitonacylindricollis* Fåhraeus, 1840†

*Sitonahispidulus* (Fabricius, 1777)†●

*Sitonalepidus* Gyllenhal, 1834†

*Sitonalineellus* (Bonsdorff, 1785)*

**Tribe Trachyphloeini Gistel, 1848**

Subtribe Trachyphloeina Gistel, 1848

*Cathormiocerusaristatus* (Gyllenhal, 1827)†

*Romualdiusbifoveolatus* (Beck, 1817)†

**Tribe Tropiphorini Marseul, 1863**

*Phyxelisrigidus* (Say, 1832)

*Tropiphorusterricola* (Newman, 1838)†

**Subfamily HYPERINAE Marseul, 1863**

**Tribe Hyperini Marseul, 1863**

Brachypera (Antidonus) zoilus (Scopoli, 1763)†

*Hyperacastor* (LeConte, 1876)

*Hyperameles* (Fabricius, 1792)†

*Hyperanigrirostris* (Fabricius, 1775)†●

*Hyperapostica* (Gyllenhal, 1813)†

**Subfamily MESOPTILIINAE Lacordaire, 1863**

**Tribe Magdalidini Pascoe, 1870**

***Magdalisalutacea* LeConte, 1878**●

*Magdalisbarbita* (Say, 1832)

***Magdalishispoides* LeConte, 1876**●

*Magdalispiceae* Buchanan, 1934●

**Subfamily MOLYTINAE Schönherr, 1823**

**Tribe Conotrachelini Jekel, 1865**

*Conotrachellusnenuphar* (Herbst, 1797)●

**Tribe Hylobiini Kirby, 1837**

Subtribe Hylobiina Kirby, 1837

*Hylobiuscongener* Dalla Torre, Schenkling & Marshall, 1932●

*Hylobiuspinicola* (Couper, 1864)

**Tribe Pissodini Gistel, 1848**

Subtribe Pissodina Gistel, 1848

***Pissodesaffinis* Randall, 1838**●

*Pissodesfiskei* Hopkins, 1911●

*Pissodesnemorensis* Germar, 1824●

***Pissodesrotundatus* LeConte, 1876**●

***Pissodessimilis* Hopkins, 1911**●

*Pissodesstriatulus* (Fabricius, 1775)●

*Pissodesstrobi* (Peck, 1817)●

**Subfamily SCOLYTINAE Latreille, 1804**

**Tribe Corthylini LeConte, 1876**

Subtribe Corthylina LeConte, 1876

***Gnathotrichusmateriarius* (Fitch, 1858)**●

Subtribe Pityophthorina Eichhoff, 1878

***Conophthorusconiperda* (Schwarz, 1895)**●

***Monarthrummali* (Fitch, 1855)**●

**Pityophthorus (Pityophthorus) balsameus Blackman, 1922**●

**Pityophthorus (Pityophthorus) carinatus carinatus Bright, 1978**●

**Pityophthorus (Pityophthorus) concavus Blackman, 1928**●

Pityophthorus (Pityophthorus) dentifrons Blackman, 1922●

**Pityophthorus (Pityophthorus) opaculus LeConte, 1878**●

**Pityophthorus (Pityophthorus) puberulus (LeConte, 1868)**●

**Pityophthorus (Pityophthorus) ramiperda Swaine, 1917**●

**Tribe Cryphalini Lindemann, 1877**

***Cryphalusruficollisruficollis* Hopkins, 1915**●

**Tribe Crypturgini LeConte, 1876**

*Crypturgusborealis* Swaine, 1917●

*Crypturguspusillus* (Gyllenhal, 1813)†●

**Tribe Dryocoetini Lindemann, 1877**

*Dryocoetesaffaber* (Mannerhiem, 1852)●

*Dryocoetesautographus* (Ratzeburg, 1837)*●

***Dryocoetesbetulae* Hopkins, 1894**●

*Dryocoetescaryi* Hopkins, 1915

***Lymantordecipiens* (LeConte, 1878)**●

**Tribe Hylastini LeConte, 1876**

*Hylastesopacus* Erichson, 1836†●

*Hylastesporculus* Erichson, 1836●

*Hylurgopsrugipennispinifex* (Fitch, 1858)●

*Scierusannectans* LeConte, 1876

**Tribe Hylurgini Gistel, 1848**

*Dendroctonusrufipennis* (Kirby, 1837)●

*Dendroctonussimplex* LeConte, 1868●

**Tribe Ipini Bedel, 1888**

*Ipsborealis* Swaine, 1911●

***Ips grandicollis* (Eichhoff, 1868)**●

***Ips perroti* Swaine, 1915**●

*Ips pini* (Say, 1826)●

*Orthotomicuscaelatus* (Eichhoff, 1868)●

***Orthotomicuslatidens* (LeConte, 1874)**●

***Pityogeneshopkinsi* Swaine, 1915**●

*Pityokteinessparsus* (LeConte, 1868)●

***Phloeotribuspiceae* Swaine, 1911**●

**Tribe Polygraphini Chapuis, 1869**

*Polygraphusrufipennis* (Kirby, 1837)●

**Tribe Scolytini Latreille, 1804**

***Scolytuspiceae* (Swaine, 1910)**●

*Scolytusrugulosus* (P.W.J. Müller, 1818)†

**Tribe Xyleborini LeConte, 1876**

*Anisandrusdispar* (Fabricius, 1792)†●

***Anisandrussayi* (Hopkins, 1910)**●

*Xyleborinusattenuatus* (Blandford, 1894)†●

*Xyleborinussaxeseni* (Ratzeburg, 1837)†●

*Xylosandrusgermanus* (Blandford, 1894)†●

**Tribe Xyloterini LeConte, 1876**

*Trypodendronbetulae* Swaine, 1911

*Trypodendrondomesticum* (Linnaeus, 1758)†

*Trypodendronlineatum* (Olivier, 1795)*●

*Trypodendronretusum* (LeConte, 1868)●

*Xyloterinuspolitus* (Say, 1826)●

## Footnotes

Newfoundland and Labrador are each treated separately under the current heading Distribution in Canada and Alaska.
